# BERT-TFBS: a novel BERT-based model for predicting transcription factor binding sites by transfer learning

**DOI:** 10.1093/bib/bbae195

**Published:** 2024-05-02

**Authors:** Kai Wang, Xuan Zeng, Jingwen Zhou, Fei Liu, Xiaoli Luan, Xinglong Wang

**Affiliations:** Key Laboratory of Advanced Process Control for Light Industry (Ministry of Education), School of Internet of Things Engineering, Jiangnan University, 1800 Lihu Road, Wuxi, Jiangsu 214122, China; Key Laboratory of Advanced Process Control for Light Industry (Ministry of Education), School of Internet of Things Engineering, Jiangnan University, 1800 Lihu Road, Wuxi, Jiangsu 214122, China; Science Center for Future Foods, Jiangnan University, 1800 Lihu Road, Wuxi, Jiangsu 214122, China; Key Laboratory of Industrial Biotechnology, Ministry of Education and School of Biotechnology, Jiangnan University, 1800 Lihu Road, Wuxi, Jiangsu 214122, China; Engineering Research Center of Ministry of Education on Food Synthetic Biotechnology, Jiangnan University, 1800 Lihu Road, Wuxi, Jiangsu 214122, China; Jiangsu Province Engineering Research Center of Food Synthetic Biotechnology, Jiangnan University, Wuxi 214122, China; Key Laboratory of Advanced Process Control for Light Industry (Ministry of Education), School of Internet of Things Engineering, Jiangnan University, 1800 Lihu Road, Wuxi, Jiangsu 214122, China; Key Laboratory of Advanced Process Control for Light Industry (Ministry of Education), School of Internet of Things Engineering, Jiangnan University, 1800 Lihu Road, Wuxi, Jiangsu 214122, China; Science Center for Future Foods, Jiangnan University, 1800 Lihu Road, Wuxi, Jiangsu 214122, China; Key Laboratory of Industrial Biotechnology, Ministry of Education and School of Biotechnology, Jiangnan University, 1800 Lihu Road, Wuxi, Jiangsu 214122, China

**Keywords:** transcription factor binding sites, deep learning, BERT, attention mechanisms, gene regulation, bioinformatics

## Abstract

Transcription factors (TFs) are proteins essential for regulating genetic transcriptions by binding to transcription factor binding sites (TFBSs) in DNA sequences. Accurate predictions of TFBSs can contribute to the design and construction of metabolic regulatory systems based on TFs. Although various deep-learning algorithms have been developed for predicting TFBSs, the prediction performance needs to be improved. This paper proposes a bidirectional encoder representations from transformers (BERT)-based model, called BERT-TFBS, to predict TFBSs solely based on DNA sequences. The model consists of a pre-trained BERT module (DNABERT-2), a convolutional neural network (CNN) module, a convolutional block attention module (CBAM) and an output module. The BERT-TFBS model utilizes the pre-trained DNABERT-2 module to acquire the complex long-term dependencies in DNA sequences through a transfer learning approach, and applies the CNN module and the CBAM to extract high-order local features. The proposed model is trained and tested based on 165 ENCODE ChIP-seq datasets. We conducted experiments with model variants, cross-cell-line validations and comparisons with other models. The experimental results demonstrate the effectiveness and generalization capability of BERT-TFBS in predicting TFBSs, and they show that the proposed model outperforms other deep-learning models. The source code for BERT-TFBS is available at https://github.com/ZX1998-12/BERT-TFBS.

## INTRODUCTION

Transcription factors (TFs) are proteins that play pivotal roles in regulating genetic transcriptions by binding to nucleotide sequences in the DNA upstream regions of genes [1, 2]. These sequences are referred to as transcription factor binding sites (TFBSs), and they are typically short motifs composed of 6–12 base pairs (bp) [3]. Research shows that the bindings of these motifs by TFs are influenced by the sequence contexts [4]. Therefore, accurate predictions of TFBSs from a DNA sequence are essential to investigate the mechanisms and physiological functions of TFs, and can contribute to discovering how motif combinations and their syntactic arrangements affect the bindings of TFs to DNA sequences *in vivo* [5]. This in turn aids in the design and construction of metabolic regulatory systems based on TFs in the fields of metabolic engineering and synthetic biology [6, 7]. In addition, research suggests that variations in TFBSs may be related to certain serious diseases [8]. In practical applications, TFBS prediction methods support drug design and the mutation and synthesis of regulatory elements such as promoters and enhancers, and they can facilitate the development of bioinformatics tools.

With the development of sequencing techniques and high-throughput sequencing technologies, experimental methods such as chromatin immunoprecipitation sequencing (ChIP-seq) [9] and selective microfluidics-based ligand enrichment followed by sequencing (SMiLE-seq) [10] can be implemented to identify the DNA fragments that interact with TFs. However, these methods are costly and rely on experimental conditions. Therefore, it is essential to develop cost-effective and accurate computational methods.

In recent years, machine learning and deep learning have found extensive use in the field of bioinformatics, such as gene expression prediction [11], drug discovery [12] and protein function prediction [13]. For TFBS predictions, many computational prediction methods use traditional machine learning approaches, for instance, support vector machines [14], random forests [15, 16] and hidden Markov models [17]. Moreover, several deep-learning TFBS prediction models have been proposed to improve the prediction performance. For instance, DeepBind [18] and DeepSEA [19] use convolutional neural networks (CNNs) and a one-hot encoding mechanism to extract the features of DNA sequences for TFBS predictions. HOCNN [20] enhances the prediction accuracy using high-order nucleotide encoding and multi-scale convolution layers. AgentBind [5] and FCNA [21] are model interpretation techniques to identify TFBS motifs. However, CNNs have limited capabilities of capturing long-distance dependencies between different positions within a DNA sequence. To address this issue, DanQ [22] and DeepSite [23] combine CNNs with Bi-LSTM networks to learn long-distance dependencies in sequences, significantly improving the TFBS prediction performance. Furthermore, with the development of attention mechanisms [24], models such as DeepGRN [25], SAResNet [26], D-SSCA [27], DSAC [28] and DeepSTF [29] successfully utilize attention modules to improve prediction performance. Visualizations of attention module weights within the model allow for exploring model interpretability.

Recently, pre-training and transfer learning methods with transformer architectures have been applied to natural language processing [30, 31]. For instance, bidirectional encoder representations from transformers (BERT) [32] can effectively learn contextual information by introducing a masking mechanism. The BERT model can be pre-trained with large-scale unlabeled general text data, allowing it to grasp a broad range of features and patterns in textual information. While initially designed for natural language processing tasks, BERT-based models can also be applied to other domains, such as bioinformatics, where DNA and protein sequences can be treated as text data [33, 34].

In this paper, a novel TFBS prediction model, named BERT-TFBS, is proposed. It consists of a pre-trained BERT model, a CNN module, a convolutional block attention module (CBAM) and an output module. TFBS prediction with this model is solely based on DNA sequence information. To validate the effectiveness of BERT-TFBS, we assessed its prediction performance using 165 ChIP-seq datasets based on the method by Zeng *et al*. [35]. The results indicate that BERT-TFBS outperforms existing models in terms of predicting TFBSs.

The main contributions of our study are as follows:

(1) We propose a novel deep-learning model (BERT-TFBS) for predicting TFBSs. It combines a pre-trained BERT model with a CNN module, a CBAM and an output module. Our study represents the pioneering use of transfer learning with a pre-trained model for predicting TFBSs.
(2) An ablation study is conducted by comparing the prediction performance of BERT-TFBS with that of two variant models, in order to show the contributions of the CNN module and the CBAM to BERT-TFBS.
(3) Cross-cell-line validation experiments are conducted to evaluate the generalization capability and robustness of BERT-TFBS in predicting TFBSs.
(4) The prediction performance of the proposed model is better than that of existing models.

## MATERIALS AND METHODS

### Benchmark dataset

As benchmark datasets, we selected 165 ChIP-seq datasets generated by the Encyclopedia of DNA Elements (ENCODE) project [36], encompassing 29 different TFs from 32 various cell lines. Based on the approach of Zeng *et al*. [35], each of the datasets is randomly divided into a training subset (80% of the samples) and a testing subset (the remaining 20% of the samples), where each positive sample is a 101 bp DNA sequence that was experimentally confirmed to contain TFBSs, and each negative sample is the sequence that is obtained from a positive sequence through random permutations while preserving the nucleotide frequencies. The above datasets can be downloaded at http://hgdownload.cse.ucsc.edu/goldenPath/hg19/encodeDCC/wgEncodeAwgTfbsUniform/. Detailed descriptions of the 165 ChIP-seq datasets are given in Supplementary Table S1.

### Model architecture

#### Overall framework

**Figure 1:**
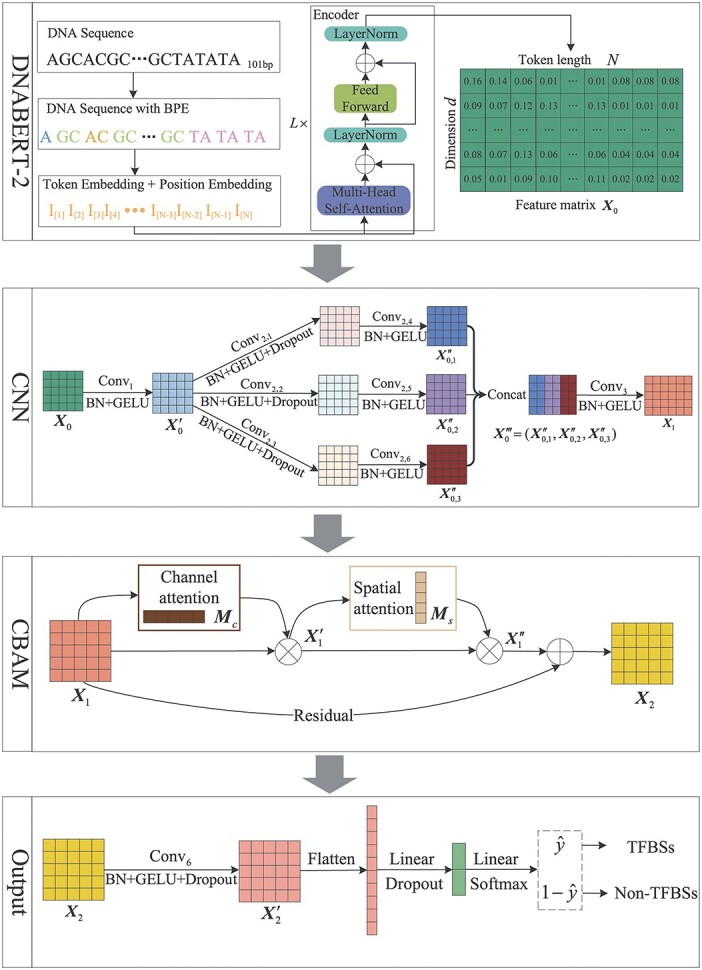
Overall architecture of BERT-TFBS, which consists of a DNABERT-2 module, a CNN module, a CBAM and an output module.

The overall framework of the proposed BERT-TFBS is shown in Figure 1. The model consists of four modules: a pre-trained DNABERT-2 module, a CNN module, the CBAM and an output module. Details of the convolutional operations in BERT-TFBS are provided in Supplementary Table S2.

- DNABERT-2: This module is a pre-trained BERT model to encode DNA sequences and extract long-term dependencies within the sequences.
- CNN module: This module is utilized to extract the high-order local features of the sequence matrices by a series of convolutional layers.
- CBAM: This module is utilized to enhance local features by the spatial and channel attention mechanisms.
- Output module: This module integrates the acquired sequence features and employs a multi-layer perceptron to provide the output result of identifying TFBSs in the DNA sequences.

#### DNABERT-2

BERT is a language model that follows a pre-training and fine-tuning pattern. It begins with pre-training on a vast amount of unsupervised data to capture the fundamental syntax and semantics of DNA sequences. Subsequently, fine-tuning is carried out on task-specific annotated data to enable the model to perform a specific task.

We utilize a pre-trained BERT model called DNABERT-2 [37] as the pre-trained module for the whole architecture. DNABERT-2 applies byte pair encoding (BPE) [38] to encode DNA sequences into token embeddings. Subsequently, attention to linear biases (ALiBi) [39] is applied as positional information for the sequence and added to the token embedding to form the input matrix $\mathbf{X}$. The global feature matrix $\mathbf{X}_{0}$ is obtained by passing through $L$ cascaded encoders, where each encoder consists of a multi-head self-attention mechanism block, a feedforward network and two layer normalizations. For the $l$-th encoder, the multi-head self-attention satisfies 


(1) \begin{align*}& \begin{split} \text{MultiHead}(\mathbf{X}^{(l)})=\mathbf{W}^{O,l}\cdot\text{Concat}(&\mathbf{head}_{1}^{(l)},\ldots, \\ &\mathbf{head}_{i}^{(l)},\ldots,\mathbf{head}_{h}^{(l)}), \end{split}\end{align*}

where $\mathbf{X}^{(l)}$ is the input matrix of the $l$-th encoder, $h$ is the number of self-attention heads, $\mathbf{W}^{O,l}$ is the output transformation matrix and $\mathbf{head}_{i}^{(l)}$ is the output of the $i$-th head. Here, $\mathbf{head}_{i}^{(l)}$ can be expressed as 


(2) \begin{align*}& \mathbf{head}_{i}^{(l)}= \mathbf{W}_{i}^{V,l}\mathbf{X^{(l)}}\cdot\text{Softmax}\left(\frac{(\mathbf{W}_{i}^{K,l}\mathbf{X}^{(l)})^{T}\cdot \mathbf{W}_{i}^{Q,l}\mathbf{X}^{(l)}}{\sqrt{d_{K}}}\right),\end{align*}

where $\mathbf{W}_{i}^{Q,l}$, $\mathbf{W}_{i}^{K,l}$ and $\mathbf{W}_{i}^{V,l}$, respectively, denote the query, key and value transformation matrices for the $i$-th head, and $d_{K}$ denotes the dimensionality of the key matrix.

Then, through the residue connection between $\mathbf{X}^{(l)}$ and $\text{MultiHead}(\mathbf{X}^{(l)})$, and following layer normalization, the feature matrix $\mathbf{Y}^{(l)}$ satisfies 


(3) \begin{align*}& \mathbf{Y}^{(l)}= \text{LayerNorm}(\mathbf{X}^{(l)}+\text{MultiHead}(\mathbf{X}^{(l)})).\end{align*}

Furthermore, passing through a feedforward network generates $\text{FFN}$($\mathbf{Y}^{(l)}$), and the residue connection between $\mathbf{Y}^{(l)}$ and $\text{FFN}$($\mathbf{Y}^{(l)}$) followed by layer normalization implies the output of the $l$-th encoder: 


(4) \begin{align*}& \mathbf{X}^{(l+1)} = \text{LayerNorm}(\mathbf{Y}^{(l)}+\text{FFN}(\mathbf{Y}^{(l)})).\end{align*}

Finally, passing through the cascaded $L$ encoders, the output of DNABERT-2 can be obtained as 


(5) \begin{align*}& \mathbf{X}_{0}= \mathbf{X}^{(L+1)}\in\mathbb R^{d\times N},\end{align*}

where $d$ represents the dimension of the word vector, and $N$ represents the number of tokens.

#### CNN module

The CNN module applies a series of convolutional layers to extract high-order local features [40] from the feature matrix $\mathbf{X}_{0}$, which consists of a beginning convolutional block, a parallel convolutional block and an ending convolutional block. The beginning convolutional block includes a convolutional operation $\text{Conv}_{1}$, a batch normalization (BN) [41] and a Gaussian error linear unit (GELU) activation function [42], which is expressed as 


(6) \begin{align*}& \mathbf{X}^{\prime}_{0}=\text{GELU}(\text{BN}(\text{Conv}_{1}(\mathbf{X}_{0}))).\end{align*}

The parallel convolutional block is divided into three parallel convolutional sub-blocks. Each of the three sub-blocks is composed of two convolutional layers. The first convolutional layer includes a convolutional operation ($\text{Conv}_{2,1}$, $\text{Conv}_{2,2}$ or $\text{Conv}_{2,3}$), a batch normalization, a GELU activation function and a dropout operation. The second convolutional layer includes a convolutional operation ($\text{Conv}_{2,4}$, $\text{Conv}_{2,5}$ or $\text{Conv}_{2,6}$), a batch normalization and a GELU activation function. Technically, each of the three sub-blocks are expressed as 


(7) \begin{align*}& \mathbf{X}^{\prime\prime}_{0,i}=\text{GELU}(\text{BN}(\text{Conv}_{2,i+3}(\text{GELU}(\text{BN}(\text{Conv}_{2,i}(\mathbf{X}^{\prime}_{0})))))),\end{align*}

where $i\in \{1,2,3\}$. Then, the three feature matrices $\mathbf{X}^{\prime\prime}_{0,1}$, $\mathbf{X}^{\prime\prime}_{0,2}$ and $\mathbf{X}^{\prime\prime}_{0,3}$ are concatenated to form 


(8) \begin{align*}& \mathbf{X}^{\prime\prime\prime}_{0}=\text{Concat}(\mathbf{X}^{\prime\prime}_{0,1},\mathbf{X}^{\prime\prime}_{0,2},\mathbf{X}^{\prime\prime}_{0,3}).\end{align*}

The ending convolutional block consists of a convolutional operation $\text{Conv}_{3}$, a batch normalization and a GELU activation function, which can be expressed as 


(9) \begin{align*}& \mathbf{X}_{1}=\text{GELU}(\text{BN}(\text{Conv}_{3}(\mathbf{X}^{\prime\prime\prime}_{0}))).\end{align*}

#### Convolutional block attention module

**Figure 2:**
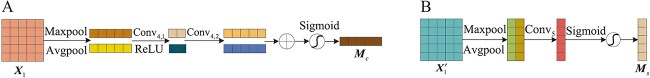
Two sub-modules of the CBAM in BERT-TFBS. (A) The channel attention sub-module. (B) The spatial attention sub-module.

In this module, channel and spatial attention mechanisms [43] are applied to enhance the features obtained from the CNN module. As shown in Figure 2A, the feature matrix $\mathbf{X}_{1}$ is first subjected to the channel attention sub-module to compute channel attention scores. This sub-module conducts separate global max-pooling and global average-pooling operations on each channel of $\mathbf{X}_{1}$. Subsequently, these two channel features are individually fed into two convolutional layers, where the first convolutional layer consists of a convolutional operation $\text{Conv}_{4,1}$ and a ReLU activation function, and the second convolutional layer comprises a convolutional operation $\text{Conv}_{4,2}$. By summing the results of these two channels and following a sigmoid activation function, the channel attention feature $\mathbf{M}_{c}$ is generated. Finally, the feature matrix $\mathbf{X}_{1}$ is subjected to element-wise multiplication (the Hadamard product) with the channel attention feature $\mathbf{M}_{c}$ through broadcasting, resulting in the output feature $\mathbf{X}^{\prime}_{1}$. The above operations can be summarized as 


(10) \begin{align*}& \begin{split} \mathbf{M}_{c} = \text{Sigmoid}(\text{Conv}_{4,2}(\text{ReLU}(\text{Conv}_{4,1}(\text{Avgpool}(\mathbf{X}_{1})))\\ + \text{Conv}_{4,2}(\text{ReLU}(\text{Conv}_{4,1}(\text{Maxpool}(\mathbf{X}_{1})))), \end{split}\end{align*}

and 


(11) \begin{align*}& \mathbf{X}^{\prime}_{1} = \mathbf{M}_{c}\odot \mathbf{X}_{1},\end{align*}

where $\odot $ denotes the element-wise multiplication.

Subsequently, the output feature matrix $\mathbf{X}^{\prime}_{1}$ of the channel attention sub-module is processed through the spatial attention sub-module. As shown in Figure 2B, this sub-module conducts global max-pooling and global average-pooling operations along the channel dimension of $\mathbf{X}^{\prime}_{1}$ to acquire spatial features. Then, these spatial features are subjected to the convolutional operation $\text{Conv}_{5}$ and a sigmoid activation function to generate the spatial attention feature $\mathbf{M}_{s}$. Finally, the feature matrix $\mathbf{X}^{\prime}_{1}$ is subjected to element-wise multiplication (the Hadamard product) with the spatial attention feature $\mathbf{M}_{s}$ through broadcasting, resulting in the output feature $\mathbf{X}^{\prime\prime}_{1}$. The above operations can be summarized as 


(12) \begin{align*}& \mathbf{M}_{s}=\text{Sigmoid}(\text{Conv}_{5}(\text{Avgpool}(\mathbf{X}^{\prime}_{1}), \text{Maxpool}(\mathbf{X}^{\prime}_{1}))),\end{align*}

and 


(13) \begin{align*}& \mathbf{X}^{\prime\prime}_{1} = \mathbf{M}_{s}\odot \mathbf{X}^{\prime}_{1}.\end{align*}

Finally, through the residue connection of $\mathbf{X}_{1}$ and $\mathbf{X}^{\prime\prime}_{1}$, the output $\mathbf{X}_{2}$ of the CBAM is 


(14) \begin{align*}& \mathbf{X}_{2} = \mathbf{X}_{1} + \mathbf{X}^{\prime\prime}_{1}.\end{align*}

#### Output module

The output module initially consolidates the features from the input matrix $\mathbf{X}_{2}$ using a convolutional block. The convolutional block comprises a convolutional operation $\text{Conv}_{6}$, a batch normalization, a GELU activation function and a dropout operation: 


(15) \begin{align*}& \mathbf{X}^{\prime}_{2}=\text{GELU}(\text{BN}(\text{Conv}_{6}(\mathbf{X}_{2}))).\end{align*}

Finally, the prediction output $\hat{y}$ of the overall architecture is obtained by flattening the feature matrix $\mathbf{X}^{\prime}_{2}$ into a column vector and following a multi-layer perceptron. Here, the multi-layer perceptron consists of two layers, where the first layer is a fully connected layer with dropout, and the second layer is a fully connected layer with a softmax activation function. The output $\hat{y}$ is the classification probability, which can predict whether an input DNA sequence contains TFBSs. The above operations can be summarized as 


(16) \begin{align*}& \hat{y}=\text{Softmax}(\text{Linear}(\text{Dropout}(\text{Linear}(\text{Flatten}(\mathbf{X}^{\prime}_{2}))))).\end{align*}

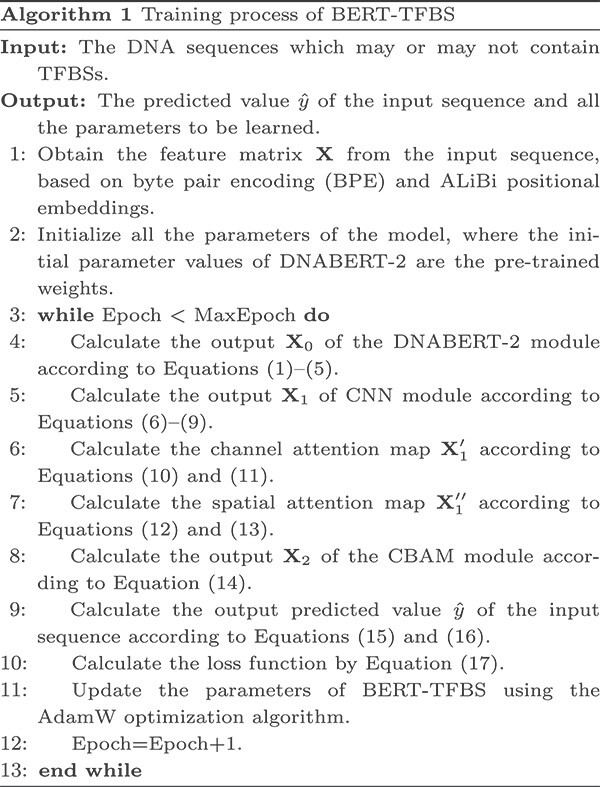


### Model training

To calculate the loss between the true labels and predicted values, the cross-entropy loss function is defined as 


(17) \begin{align*}& \text{Loss}(y,\hat{y})=-\frac{1}{n}\sum_{i=1}^{n}(y_{i}\text{log}(\hat{y}_{i})+(1-y_{i})\text{log}(1-\hat{y}_{i})),\end{align*}

where $y$ represents the true values of the DNA sequences, $\hat{y}$ represents the predicted values of BERT-TFBS and $n$ denotes the batch size of DNA sequences.

Based on the model architecture and the cross-entropy loss function, the training process of BERT-TFBS is summarized in Algorithm 1. The proposed model can be implemented and trained using PyTorch 1.12.0, with a mini-batch size of 32. The values of hyperparameters are given in Supplementary Table S3. In the training process, the AdamW optimization algorithm [44] is utilized to optimize the model parameters based on the descending gradients of the loss function. Additionally, the learning rate of the optimizer is adjusted using warm-up [45] and cosine annealing techniques [46]. The total number of training epochs is set to 15, where the warm-up period has five epochs and the cosine annealing phase has 10 epochs. To prevent instability during fine-tuning of the BERT model, a relatively low learning rate is chosen. During the warm-up phase, the learning rate systematically ascends from a small value to 1.5e-5. During the cosine annealing phase, the learning rate systematically diminishes to 2e-6 according to the cosine function. To avoid over-fitting, we apply dropout operations [47]. Therefore, the dropout rates in the output module are set to 0.4 and 0.5, but since the CNN module has a more complex structure, the dropout rate for the CNN module is set to 0.2 to increase regularization of the model.

### Evaluation metrics

Considering that the TFBS prediction models we investigate are binary classifiers, three basic metrics—accuracy, the ROC-AUC and the PR-AUC—are applied to evaluate the prediction performance.

Accuracy [48] is the proportion of correctly predicted samples, including TFBSs and non-TFBSs, to all the tested samples. It is expressed as 


\begin{align*}& \text{Accuracy}=\frac{\text{TP}+\text{TN}}{\text{TP}+\text{FN}+\text{TN}+\text{FP}}, \nonumber\end{align*}

where TP, FN, TN and FP denote the number of true positives, false negatives, true negatives and false positives, respectively. However, simply using the accuracy metric to evaluate prediction performance may result in biased prediction results when the positive (TFBS) and negative (non-TFBS) sample datasets are unbalanced. Therefore, we need to utilize other metrics, namely the ROC-AUC and PR-AUC, to evaluate the TFBS prediction performance.

The ROC-AUC [49] refers to the area under the receiver operating characteristic (ROC) curve. The higher the ROC-AUC score, the better the classification performance. The ROC curve illustrates the overall performance of a classifier at different thresholds, simultaneously considering the true positive rate (TPR) and the false positive rate (FPR). It is suitable for evaluating the performance of classifiers at different operating points. The TPR and FPR are defined as 


\begin{align*}& \text{TPR}=\frac{\text{TP}}{\text{TP}+\text{FN}} \nonumber \qquad \text{FPR}=\frac{\text{FP}}{\text{FP}+\text{TN}}. \nonumber\end{align*}

The PR-AUC [50] refers to the area under the precision–recall (PR) curve, and the higher the PR-AUC score, the better the classification performance. The PR curve depicts the performance of the classifier in the presence of imbalanced class distributions, measuring the tradeoff between precision and recall. It is more sensitive to imbalanced data and better reflects the performance of a classifier in practical applications. The definitions of precision and recall are as follows: 


\begin{align*}& \text{precision}=\frac{\text{TP}}{\text{TP}+\text{FP}} \nonumber \qquad \text{recall}=\frac{\text{TP}}{\text{TP}+\text{FN}}. \nonumber\end{align*}

In the following investigations, we combine the above three metrics (accuracy, the ROC-AUC and the PR-AUC) to comprehensively evaluate the prediction performance of the trained BERT-TFBS and other related models.

## RESULTS AND DISCUSSION

### Model variants

**Figure 3:**
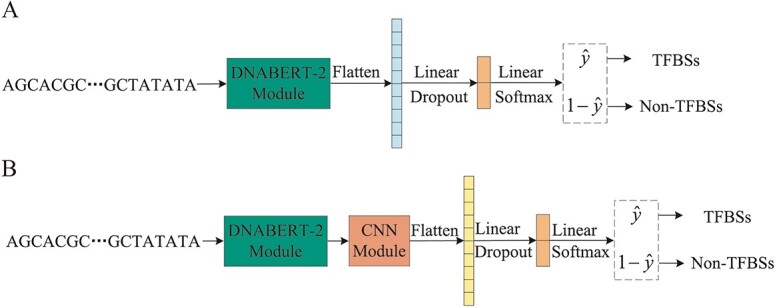
Overall frameworks of two variant models. (A) The variant model BERT-TFBS-v1. (B) The variant model BERT-TFBS-v2.

In the proposed BERT-TFBS model, the CNN module extracts higher order local features from the feature matrix, following the DNABERT-2 module. The CBAM integrates channel attention and spatial attention to enhance the representations of local features. To investigate the contributions of the CNN module and CBAM to the prediction performance of the BERT-TFBS model, we conduct an ablation study, in which we compare the prediction performance of BERT-TFBS and its variant models on 165 ChIP-seq datasets. By removing the CNN module and CBAM in BERT-TFBS, we can construct two variant models, where the frameworks of these two variant models are shown in Figure 3. We call these two variant models BERT-TFBS-v1 and BERT-TFBS-v2, and detailed descriptions of them are presented as follows.

- BERT-TFBS-v1: This variant model of BERT-TFBS is constructed by removing the CNN module, the CBAM and the convolutional layer in the output module. As shown in Figure 3A, BERT-TFBS-v1 consists of a DNABERT-2 module and a multi-layer perceptron.
- BERT-TFBS-v2: This variant model of BERT-TFBS is constructed by removing the CBAM. As shown in Figure 3B, BERT-TFBS-v2 consists of a DNABERT-2 module, a CNN module and an output module.

**Figure 4:**
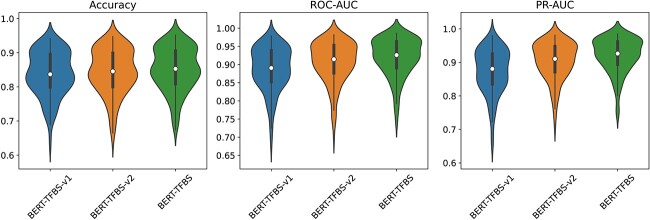
Distributions of accuracy, ROC-AUC and PR-AUC scores for BERT-TFBS and two variant models on 165 ChIP-seq datasets. The white dot inside each violin represents the median, the bold black line inside each violin represents the interquartile range and the two vertical thin black lines inside each violin extend to the minimum and maximum non-outlier values.

These two variant models can be similarly trained and tested on 165 ChIP-seq datasets according to the approach of BERT-TFBS. For each of 165 ChIP-seq datasets, we randomly select 80% of all the sequence samples as the training subset, and the remaining 20% as the testing subset. The distributions results of the accuracy, ROC-AUC and PR-AUC scores for the trained BERT-TFBS, BERT-TFBS-v1 and BERT-TFBS-v2 models on the testing subsets of 165 ChIP-seq datasets are shown in Figure 4, and the corresponding average values are presented in Table 1. More detailed prediction results are given in Supplementary Table S4, showing the accuracy, ROC-AUC and PR-AUC scores for BERT-TFBS and its two variant models on the 165 ChIP-seq datasets.

**Table 1:** Average values of accuracy, ROC-AUC and PR-AUC scores for BERT-TFBS and its two variant models on 165 ChIP-seq datasets.

Model	Accuracy	ROC-AUC	PR-AUC
BERT-TFBS-v1	0.838	0.887	0.872
BERT-TFBS-v2	0.844	0.906	0.904
**BERT-TFBS**	**0.851**	**0.919**	**0.920**

According to the TFBS prediction performance of these models, we can observe that BERT-TFBS outperforms its two variant models. BERT-TFBS-v2 is the variant model without the CBAM, and BERT-TFBS-v1 is the variant model without the CNN module and the convolutional layer in the output module. The results show that the relative improvements of BERT-TFBS over BERT-TFBS-v1 in the average values of accuracy, ROC-AUC and PR-AUC scores are 1.3%, 3.2% and 4.8%, respectively. Moreover, the relative improvements of BERT-TFBS over BERT-TFBS-v2 in the average scores of accuracy, ROC-AUC and PR-AUC are 0.7%, 1.3% and 1.6%, respectively. In addition, compared with BERT-TFBS-v1 and BERT-TFBS-v2, BERT-TFBS provides higher values of medians, upper quartiles, lower quartiles, maximums and minimums of accuracy, ROC-AUC and PR-AUC scores across the 165 ChIP-seq datasets. For instance, the median values of accuracy, ROC-AUC and PR-AUC scores for BERT-TFBS are 0.853, 0.926 and 0.926, respectively, which are 1.6%, 3.6% and 4.6% higher than BERT-TFBS-v1, and 0.7%, 1.1% and 1.6% higher than BERT-TFBS-v2, respectively. The upper quartile (resp. lower quartile) values of accuracy, ROC-AUC and PR-AUC scores for BERT-TFBS are 0.906, 0.963 and 0.960 (resp. 0.809, 0.891 and 0.894), which are 1.1%, 2.5% and 3.5% (resp. 1.0%, 3.8% and 5.8%) higher than BERT-TFBS-v1, and 0.7%, 1.0% and 1.2% (resp. 0.9%, 1.4% and 2.2%) higher than BERT-TFBS-v2, respectively.

Taken together, the experimental results show that applying the CNN module and the convolutional layer in the output module to extract high-order local features considerably enhances the TFBS prediction performance. Moreover, utilizing the CBAM, which enhances local features, can further improve the TFBS prediction performance. The results demonstrate that BERT-TFBS-v1, which only consists of the DNABERT-2 module and the multi-layer perceptron, results in the worst prediction performance, due to the absence of local feature extraction following DNABERT-2. Although BERT-TFBS-v2 utilizes the CNN module and the convolutional layer in the output module followed by the multi-layer perceptron, its prediction ability is still worse than BERT-TFBS, which includes the CBAM. This ablation study illustrates the significance of the channel and spatial attention mechanisms to extract features of DNA sequences.

### Cross-cell-line validation

**Figure 5:**
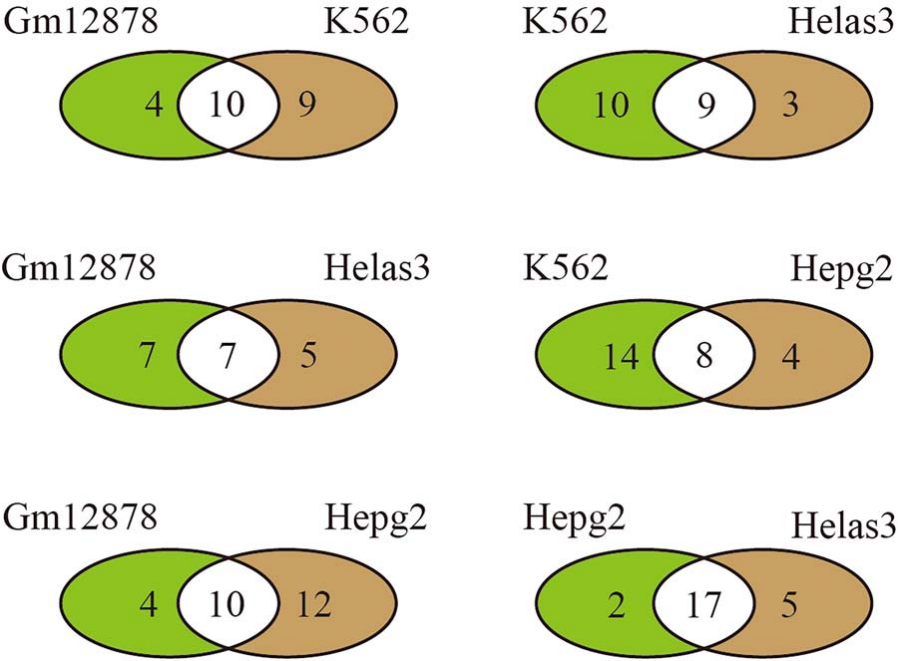
The overlap of TFs across GM12878, K562, Helas3 and Hepg2 cells.

To validate the generalization and robustness capabilities of BERT-TFBS in learning the general patterns of a specific TF, we conduct the model across different cell lines to predict the binding sites of CTCF to DNA sequences in target cell lines. We choose GM12878, Helas3, Hepg2 and K562 cell lines for the cross-cell-line validations. The reason for choosing these four cell lines is that they contain many overlapping TFs, including CTCF, GABP, FOS, P300, JUND and MAX, as shown in Figure 5. In the same cell line, all sequences bound by CTCF are chosen as positive samples, and sequences bound by other TFs are chosen as negative samples. This process results in four trained BERT-TFBS models corresponding to the GM12878, Helas3, Hepg2 and K562 cell lines. Subsequently, these four trained models are used to predict whether the actual binding sites in the four cell lines are sequences bound by CTCF. Supplementary Tables S6–S9 present the accuracy scores of the four trained models corresponding to the GM12878, Helas3, Hepg2 and K562 cell lines, respectively, for predicting TFBSs in the testing samples of the datasets belonging to any of the four cell lines. The testing samples consist of DNA sequences bound by TFs, where sequences bound by CTCF are chosen as positive samples, and sequences bound by other TFs are chosen as negative samples.

**Figure 6:**
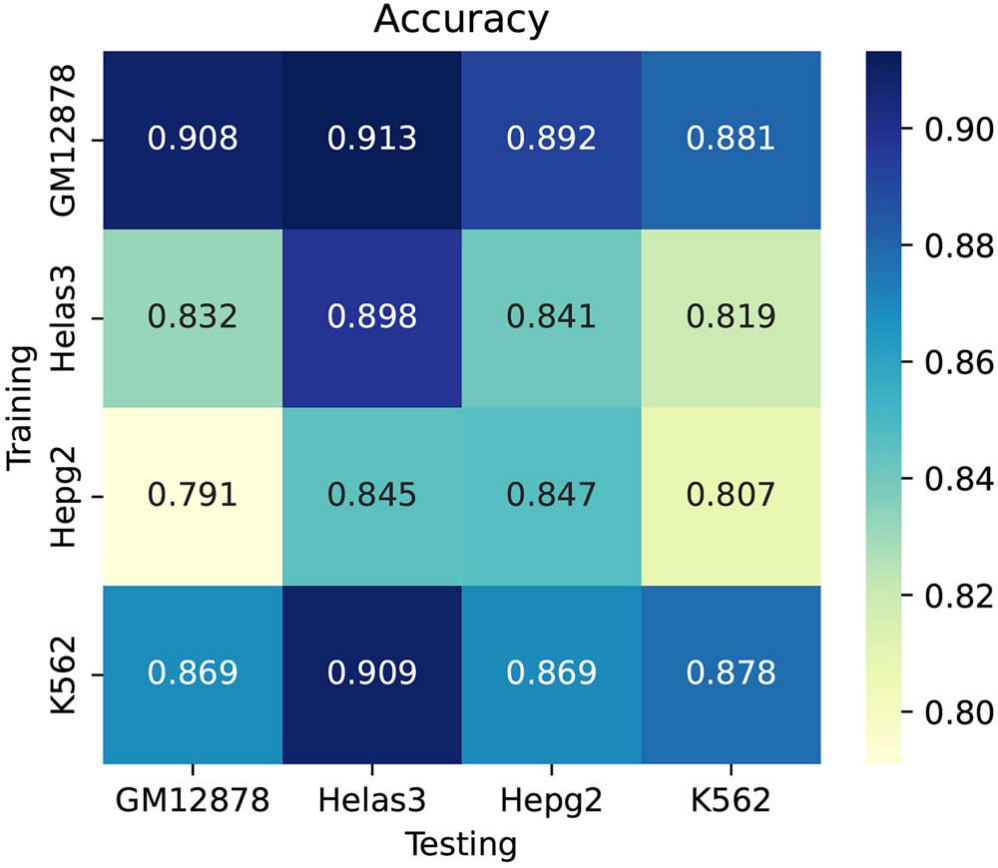
Performance comparisons between cross-cell-line validations and conventional validations of BERT-TFBS for predicting CTCF binding sequences in terms of accuracy metrics.

Figure 6 shows the average values of accuracy scores for the BERT-TFBS models trained on GM12878, Helas3, Hepg2 and K562 cell lines to predict the testing samples of the datasets corresponding to these four cell lines. The diagonal prediction results correspond to the conventional validations when the training and testing samples belong to the same cell line. The non-diagonal prediction results correspond to the cross-cell-line validations when the training and testing samples belong to different cell lines. According to the TFBS prediction performance in Figure 6, the prediction performance of conventional validations is better than most cross-cell-line validations. For instance, considering the BERT-TFBS model trained on the Helas3 cell line, the average accuracy score is 0.898 for the testing subsets corresponding to the Helas3 cell line, which is 6.6%, 5.7% and 7.9% higher than the GM12878, Hepg2 and K562 cell lines, respectively. However, the cross-cell-line predictions still maintain high performance scores, with accuracy scores ranging from 0.791 to 0.909.

**Figure 7:**
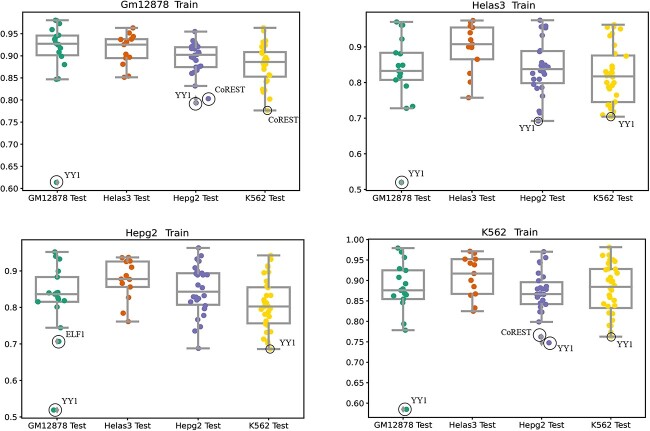
Distributions of accuracy scores of the BERT-TFBS models trained on Helas3, Hepg2, GM12878 and K562 cell lines. The colored dots represent the scores of individual datasets. The gray line inside each box represents the median. The bottom and top gray edges of each box represent the lower and upper quartiles, respectively. The two vertical gray lines outside each box extend to the minimum and maximum values. And the diamond-shaped gray markers indicate outliers.

Figure 7 shows the distributions of accuracy scores for the BERT-TFBS models trained on the GM12878, Helas3, Hepg2 and K562 cell lines to predict the testing samples of the datasets corresponding to these cell lines. Clearly, the conventional validation shows higher values of the median, lower quartile, upper quartile, minimum and maximum accuracy scores. For instance, considering the BERT-TFBS model trained on the Helas3 cell line, the median (resp. lower quartile, upper quartile) values of accuracy scores are 0.833 (resp. 0.807, 0.883), 0.907 (resp. 0.865, 0.954), 0.837 (resp. 0.798, 0.888) and 0.817 (resp. 0.745, 0.876) on the testing subsets corresponding to the GM12878, Helas3, Hepg2 and K562 cell lines, respectively.

However, the BERT-TFBS models trained on GM12878, HepG2 and K562 cell lines exhibit better predictive performance in cross-cell-line validations than conventional validations. According to the work by Schwalie *et al* [51], BERT-TFBS models often predict sequences bound by YY1 as sequences bound by CTCF, potentially attributed to the similarity between CTCF-YY1 co-bound regions and regions bound solely by YY1. Furthermore, according to Figure 7, it is observed that the model also frequently misclassifies sequences bound by CoREST in HepG2 and K562 cell lines, indicating the possible interaction between CoREST and CTCF.

In summary, the results of cross-cell-line validations and conventional validations show that BERT-TFBS provides high performance in the cross-cell-line predictions, although the cross-cell-line prediction performance is lower than the conventional prediction performance. The results demonstrate that TFs functioning within specific cell lines, potentially exhibiting distinct binding patterns and functionalities across diverse cellular contexts, play a more significant role in TFs-DNA identifications compared with those with consistent binding patterns and functionalities across multiple cell lines. Furthermore, the BERT-TFBS model proposed in this paper has perfect generalization and robustness capabilities, and can accurately predict whether the TF binding to a sequence is the specific TF in different cell lines.

### Comparison with other models

Finally, a comprehensive evaluation is conducted to compare the prediction performance of BERT-TFBS with six benchmark models. For these benchmark models, DeepBind [18] utilizes the one-hot encoding method and a CNN architecture, DanQ [22] combines the CNN and Bi-LSTM architectures, DLBSS [52] applies a shared CNN, CRPTS [53] utilizes a CNN–RNN framework and D-SSCA [27] and DSAC [28] incorporate CNNs and attention mechanisms. As above, each of the 165 ChIP-seq datasets is randomly divided into a training subset that contains 80% of the samples and a testing subset that contains the remaining 20%. BERT-TFBS and these benchmark models are individually trained on the training subsets of all datasets, resulting in trained BERT-TFBS models and the six benchmark models corresponding to 165 ChIP-seq datasets. The detailed prediction performance of BERT-TFBS and the benchmark models in terms of accuracy, ROC-AUC and PR-AUC scores on the 165 ChIP-seq datasets is provided in Supplementary Table S10.

The average values of accuracy, ROC-AUC and PR-AUC scores of BERT-TFBS and the benchmark models on the 165 ChIP-seq datasets are compared in Table 2. According to the TFBS prediction performance of these models, the BERT-TFBS outperforms these benchmark models. Specifically, the average accuracy, ROC-AUC and PR-AUC scores of BERT-TFBS are 0.851, 0.919 and 0.920, respectively, which are 3.5%, 3.2% and 2.9% higher than those of DSAC, and are 0.9%, 7.2% and 6.5% higher than those of DanQ, respectively. Here, DSAC and DanQ are the TFBS prediction models with the best and worst performance, respectively, among the six benchmark models.

**Table 2:** Average values of the accuracy, ROC-AUC and PR-AUC scores of BERT-TFBS and benchmark models on the 165 ChIP-seq datasets.

Model	Accuracy	ROC-AUC	PR-AUC
DanQ [22]	0.782	0.849	0.855
DeepBind [18]	0.785	0.853	0.858
CRPTS [53]	0.793	0.862	0.867
DLBSS [52]	0.793	0.865	0.871
D-SSCA [27]	0.793	0.867	0.871
DSAC [28]	0.816	0.887	0.891
**BERT-TFBS**	**0.851**	**0.919**	**0.920**

**Figure 8:**
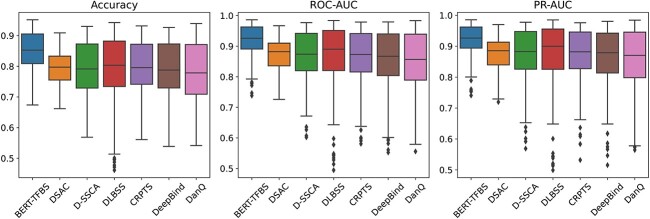
Prediction performance of BERT-TFBS compared with benchmark models on 165 ChIP-seq datasets. The line inside each box represents the median. The bottom and top edges of each box represent the lower and upper quartiles, respectively. The two vertical lines outside each box extend to the minimum and maximum non-outlier values, and the diamond-shaped markers indicate outliers.

Figure 8 presents the distributions of accuracy, ROC-AUC and PR-AUC scores of BERT-TFBS and the six benchmark models on the testing subsets of the 165 ChIP-seq datasets. Clearly, BERT-TFBS surpasses these benchmark models, with higher values of medians, lower quartiles, upper quartiles, maximums and minimums in terms of the accuracy, ROC-AUC and PR-AUC. Specifically, the median values of accuracy, ROC-AUC and PR-AUC scores of BERT-TFBS are 0.853, 0.926 and 0.926, respectively, which are 5.5%, 4.4% and 4.1% higher than those of the best benchmark model, DSAC, and 7.7%, 6.9% and 5.6% higher than the worst benchmark model, DanQ. The upper quartile (resp. lower quartile) values of accuracy, ROC-AUC and PR-AUC scores of BERT-TFBS are 0.906, 0.963 and 0.962 (resp. 0.809, 0.891 and 0.894), respectively, which are 7.2%, 5.3% and 4.9% (resp. 5.4%, 5.5% and 5.4%) higher than those of the best benchmark model, DSAC, and 3.5%, 2.4% and 1.7% (resp. 10.0%, 10.2% and 9.6%) higher than the worst benchmark model, DanQ.

Table 1 and Table 2 show that the two variant models of BERT-TFBS also outperform the six benchmark models in terms of the average accuracy, ROC-AUC and PR-AUC. Taken together, the experimental results demonstrate that BERT-TFBS and its variant models outperform DeepBind and DLBSS, which contain the CNN architectures. This indicates that combining the long-term dependencies and local features in sequences considerably enhances the TFBS prediction performance. BERT-TFBS and its variant models show better prediction performance than DanQ and CRPTS, which utilize the CNN–RNN framework. This demonstrates the superior ability of the multi-head self-attention mechanism in DNABERT-2 to extract long-term dependencies over RNNs. Additionally, BERT-TFBS and its variant models outperform D-SSCA and DSAC, which utilize attention mechanisms. This indicates that the transfer learning approach helps with complex feature extractions in DNA sequences and enhances the generalization capabilities of the models.

In addition, analyzing the data in Supplementary Table S10, we found that models trained using the datasets on FOS, CTCF and CEBPB TFs demonstrate perfect and uniform prediction performance across all cell lines. However, models trained using the datasets on HDAC and EZH2 TFs differed significantly across different cell lines in terms of performance. This suggests that the binding patterns and regulatory roles of HDAC and EZH2 TFs may vary considerably between different cell types. Such variability could be attributed to cell-specific chromatin landscapes, cellular signaling pathways and gene expression profiles, highlighting the complexity of transcriptional regulation in diverse cellular contexts [36, 54].

Furthermore, we can utilize BERT-TFBS models to predict whether a random sequence in specific cell lines contains a TFBS and which type of TFs it is bound by. The sequence is fed into a set of BERT-TFBS models trained on different types of TFs in the specific cell line, and then a multi-classification is conducted based on the outputs of the models to determine which type of TFs the sequence may be bound by.

In summary, the experimental results demonstrate the superiority of the proposed BERT-TFBS over all the existing benchmark models on the 165 ChIP-seq datasets. The results indicate that we can enhance the ability to predict TFBSs by applying pre-trained language models and transfer learning, together with other modules such as the CNN module and the CBAM.

## CONCLUSION

In this paper, we proposed a novel BERT-based model, named BERT-TFBS, to discover the motif combinations of TFBSs from a similar nucleotide background [35]. The proposed model consists of a pre-trained DNABERT-2 module, a CNN module, a CBAM and an output module. Among these modules in BERT-TFBS, the DNABERT-2 module is a pre-trained BERT model that successfully extracts the complex long-term dependencies in the DNA sequences through transfer learning. The CNN module and the CBAM further extract high-order local features following DNABERT-2. BERT-TFBS was trained and tested on 165 ChIP-seq datasets. It was compared with variant models and other existing deep-learning models, and cross-cell-line validation experiments were conducted. The ROC-AUC and PR-AUC metrics of accuracy were applied to evaluate the prediction performance.

By comparing the TFBS prediction performance of BERT-TFBS with its variant models, we found that employing the CNN module and the CBAM enhanced the prediction ability. The cross-cell-line validations showed the generalization capability of BERT-TFBS in predicting TFBSs. In addition, experimental results demonstrated that the proposed BERT-TFBS model provides the best prediction performance compared with other existing deep-learning models. To our knowledge, this study is the first to improve the ability to predict TFBSs based on deep-learning approaches by applying a pre-trained language model and the methodology of transfer learning. In the long term, the investigations of this paper could contribute to studying metabolic regulatory systems based on TFs.

Although we demonstrated that the proposed BERT-TFBS provides remarkable accuracy when predicting TFBSs, further explorations should be conducted in the future. For instance, by combining DNA sequence information with DNA structural characteristics, we might enhance the prediction ability of the model. Additionally, biological experiments could be conducted to validate the prediction results.

**Key Points**

- BERT-TFBS is a novel BERT-based model that predicts TFBSs in DNA sequences based solely on sequence information. This model consists of a pre-trained DNABERT-2 module, a CNN module, a CBAM and an output module.
- It is the first time a pre-trained language model (that is, the DNABERT-2 module in BERT-TFBS) has been used to predict TFBSs in DNA sequences. Transfer learning is applied to fine-tune the parameters in the pre-trained DNABERT-2 to capture the complex long-term dependencies in DNA sequences.
- By comparing BERT-TFBS with its two variant models, ablation experiments demonstrate that utilizing the CBAM and the convolutional layer in the output module further enhances the ability to predict TFBSs.
- Cross-cell-line validation results show that BERT-TFBS has perfect generalization capability for TFBS predictions in different cell lines.
- Based on 165 ChIP-seq datasets, experimental results show that BERT-TFBS outperforms other deep-learning models at predicting TFBSs.

## Supplementary Material

Supplementary-Table_bbae195
