# Supplementary material for: BERT-TFBS: a novel BERT-based model for predicting transcription factor binding sites by transfer learning
Source: Brief Bioinform. 2024 May 2;25(3):bbae195. doi: 10.1093/bib/bbae195 (PMC11066948; doi:10.1093/bib/bbae195)
Supplement: Supplementary-Table_bbae195 [file supplementary-table_bbae195.pdf]

**Supplementary Table S1.** 165 ChIP-seq datasets comprising 29 TFs in our work			
**TFs**	**Datasets**	**Train_number**	**Test_number**
CEBPB	wgEncodeAwgTfbsHaibHepg2Cebpbsc150V0416101UniPk	28491	7123
	wgEncodeAwgTfbsHaibK562Cebpbsc150V0422111UniPk	34747	8687
	wgEncodeAwgTfbsSydhA549CebpbIggrabUniPk	60745	15187
	wgEncodeAwgTfbsSydhH1hescCebpbIggrabUniPk	24472	6118
	wgEncodeAwgTfbsSydhHelas3CebpbIggrabUniPk	94892	23724
	wgEncodeAwgTfbsSydhHepg2CebpbForsklnUniPk	29012	7254
	wgEncodeAwgTfbsSydhHepg2CebpbIggrabUniPk	89115	22279
	wgEncodeAwgTfbsSydhImr90CebpbIggrabUniPk	109712	27428
	wgEncodeAwgTfbsSydhK562CebpbIggrabUniPk	60416	15104
CoREST	wgEncodeAwgTfbsSydhHepg2Corestsc30189IggrabUniPk	8515	2129
	wgEncodeAwgTfbsSydhK562Corestsc30189IggrabUniPk	56211	14053
CTCF	wgEncodeAwgTfbsBroadDnd41CtcfUniPk	77531	19383
	wgEncodeAwgTfbsBroadGm12878CtcfUniPk	69435	17359
	wgEncodeAwgTfbsBroadH1hescCtcfUniPk	103174	25794
	wgEncodeAwgTfbsBroadHelas3CtcfUniPk	81049	20263
	wgEncodeAwgTfbsBroadHepg2CtcfUniPk	72324	18082
	wgEncodeAwgTfbsBroadHmecCtcfUniPk	61801	15451
	wgEncodeAwgTfbsBroadHsmmCtcfUniPk	79036	19760
	wgEncodeAwgTfbsBroadHsmmtCtcfUniPk	74404	18602
	wgEncodeAwgTfbsBroadHuvecCtcfUniPk	58102	14526
	wgEncodeAwgTfbsBroadK562CtcfUniPk	80417	20105
	wgEncodeAwgTfbsBroadNhaCtcfUniPk	59652	14914
	wgEncodeAwgTfbsBroadNhdfadCtcfUniPk	75851	18963
	wgEncodeAwgTfbsBroadNhekCtcfUniPk	72955	18239
	wgEncodeAwgTfbsBroadNhlfCtcfUniPk	61337	15335
	wgEncodeAwgTfbsBroadOsteoblCtcfUniPk	86148	21538
	wgEncodeAwgTfbsUtaGm12878CtcfUniPk	75536	18884
	wgEncodeAwgTfbsUtaH1hescCtcfUniPk	67601	16901
	wgEncodeAwgTfbsUtaHelas3CtcfUniPk	90883	22721
	wgEncodeAwgTfbsUtaK562CtcfUniPk	86808	21702
	wgEncodeAwgTfbsUtaMcf7CtcfUniPk	90049	22513
HaE2F1	wgEncodeAwgTfbsSydhHelas3Hae2f1UniPk	15990	3998
	wgEncodeAwgTfbsSydhMcf7Hae2f1UcdUniPk	30704	7676
EZH2	wgEncodeAwgTfbsBroadDnd41Ezh239875UniPk	2681	671
	wgEncodeAwgTfbsBroadHelas3Ezh239875UniPk	2840	710
	wgEncodeAwgTfbsBroadHepg2Ezh239875UniPk	5235	1309
	wgEncodeAwgTfbsBroadHsmmEzh239875UniPk	2464	616
	wgEncodeAwgTfbsBroadHuvecEzh239875UniPk	6788	1698
	wgEncodeAwgTfbsBroadK562Ezh239875UniPk	2684	672
FOS	wgEncodeAwgTfbsHaibA549Fosl2V0422111Etoh02UniPk	44750	11188
	wgEncodeAwgTfbsHaibH1hescFosl1sc183V0416102UniPk	1758	440
	wgEncodeAwgTfbsHaibHepg2Fosl2V0416101UniPk	39678	9920
	wgEncodeAwgTfbsHaibK562Fosl1sc183V0416101UniPk	17569	4393
	wgEncodeAwgTfbsSydhGm12878CfosUniPk	3488	872
	wgEncodeAwgTfbsSydhHelas3CfosUniPk	14494	3624
	wgEncodeAwgTfbsSydhHuvecCfosUcdUniPk	72961	18241
	wgEncodeAwgTfbsSydhK562CfosUniPk	11968	2992
	wgEncodeAwgTfbsSydhMcf10aesCfosTam112hHvdUniPk	143425	35857
	wgEncodeAwgTfbsSydhMcf10aesCfosTam14hHvdUniPk	134080	33520
	wgEncodeAwgTfbsSydhMcf10aesCfosTamHvdUniPk	109454	27364
	wgEncodeAwgTfbsUchicagoK562EfosUniPk	16120	4030
FOXA1	wgEncodeAwgTfbsHaibA549Foxa1V0416102Dex100nmUniPk	12054	3014
	wgEncodeAwgTfbsHaibEcc1Foxa1sc6553V0416102Dm002p1hUniPk	9283	2321
	wgEncodeAwgTfbsHaibHepg2Foxa1sc101058V0416101UniPk	67550	16888
	wgEncodeAwgTfbsHaibHepg2Foxa1sc6553V0416101UniPk	80224	20056
	wgEncodeAwgTfbsHaibT47dFoxa1sc6553V0416102Dm002p1hUniPk	65257	16315
GABP	wgEncodeAwgTfbsHaibA549GabpV0422111Etoh02UniPk	18315	4579
	wgEncodeAwgTfbsHaibGm12878GabpPcr2xUniPk	10211	2553
	wgEncodeAwgTfbsHaibH1hescGabpPcr1xUniPk	8968	2242
	wgEncodeAwgTfbsHaibHelas3GabpPcr1xUniPk	10494	2624
	wgEncodeAwgTfbsHaibHepg2GabpPcr2xUniPk	15710	3928
	wgEncodeAwgTfbsHaibK562GabpV0416101UniPk	22387	5597
	wgEncodeAwgTfbsHaibK562Gata2sc267Pcr1xUniPk	30081	7521
	wgEncodeAwgTfbsSydhHuvecGata2UcdUniPk	42681	10671

GATA2	wgEncodeAwgTfbsSydhK562Gata2UcdUniPk	16649	4163
	wgEncodeAwgTfbsSydhShsy5yGata2UcdUniPk	55936	13984
	wgEncodeAwgTfbsUchicagoK562Egata2UniPk	18004	4502
GATA3	wgEncodeAwgTfbsHaibT47dGata3sc268V0416102Dm002p1hUniPk	58292	14574
	wgEncodeAwgTfbsSydhMcf7Gata3UcdUniPk	9585	2397
	wgEncodeAwgTfbsSydhShsy5yGata3sc269sc269UcdUniPk	24948	6238
JUN	wgEncodeAwgTfbsSydhHelas3CjunIggrabUniPk	34089	8523
	wgEncodeAwgTfbsSydhHepg2CjunIggrabUniPk	20036	5010
	wgEncodeAwgTfbsSydhHuvecCjunUniPk	46128	11532
	wgEncodeAwgTfbsSydhK562CjunUniPk	15460	3866
JUND	wgEncodeAwgTfbsHaibHepg2JundPcr1xUniPk	34142	8536
	wgEncodeAwgTfbsSydhGm12878JundUniPk	3892	974
	wgEncodeAwgTfbsSydhH1hescJundIggrabUniPk	15019	3755
	wgEncodeAwgTfbsSydhHelas3JundIggrabUniPk	49201	12301
	wgEncodeAwgTfbsSydhHepg2JundIggrabUniPk	50878	12720
	wgEncodeAwgTfbsSydhK562JundIggrabUniPk	62753	15689
	wgEncodeAwgTfbsUchicagoK562EjundUniPk	41771	10443
MAX	wgEncodeAwgTfbsHaibK562MaxV0416102UniPk	70233	17559
	wgEncodeAwgTfbsSydhGm12878MaxIggmusUniPk	19747	4937
	wgEncodeAwgTfbsSydhH1hescMaxUcdUniPk	17553	4389
	wgEncodeAwgTfbsSydhHelas3MaxIggrabUniPk	46209	11553
	wgEncodeAwgTfbsSydhHepg2MaxIggrabUniPk	18774	4694
	wgEncodeAwgTfbsSydhHuvecMaxUniPk	14409	3603
	wgEncodeAwgTfbsSydhK562MaxIggrabUniPk	49299	12325
	wgEncodeAwgTfbsSydhNb4MaxUniPk	54438	13610
MYC	wgEncodeAwgTfbsSydhNb4CmycUniPk	41251	10313
	wgEncodeAwgTfbsUtaGm12878CmycUniPk	5830	1458
	wgEncodeAwgTfbsUtaH1hescCmycUniPk	1939	485
	wgEncodeAwgTfbsUtaHepg2CmycUniPk	7012	1754
Pol2b	wgEncodeAwgTfbsBroadHelas3Pol2bUniPk	4779	1195
	wgEncodeAwgTfbsBroadHuvecPol2bUniPk	8334	2084
	wgEncodeAwgTfbsBroadNhekPol2bUniPk	9046	2262
Sin3A	wgEncodeAwgTfbsHaibA549Sin3ak20V0422111Etoh02UniPk	9516	2380
	wgEncodeAwgTfbsHaibHepg2Sin3ak20Pcr1xUniPk	26051	6513
	wgEncodeAwgTfbsHaibK562Sin3ak20V0416101UniPk	19993	4999
	wgEncodeAwgTfbsHaibPanc1Sin3ak20V0416101UniPk	11289	2823
	wgEncodeAwgTfbsSydhH1hescSin3anb6001263IggrabUniPk	33697	8425
SP1	wgEncodeAwgTfbsHaibH1hescSp1Pcr1xUniPk	23648	5912
	wgEncodeAwgTfbsHaibHepg2Sp1Pcr1xUniPk	39952	9988
SP2	wgEncodeAwgTfbsHaibH1hescSp2V0422111UniPk	3830	958
	wgEncodeAwgTfbsHaibHepg2Sp2V0422111UniPk	4152	1038
SRF	wgEncodeAwgTfbsHaibH1hescSrfPcr1xUniPk	8009	2003
	wgEncodeAwgTfbsHaibHepg2SrfV0416101UniPk	8372	2094
	wgEncodeAwgTfbsHaibK562SrfV0416101UniPk	7414	1854
STAT3	wgEncodeAwgTfbsSydhGm12878Stat3IggmusUniPk	10185	2547
	wgEncodeAwgTfbsSydhHelas3Stat3IggrabUniPk	21563	5391
	wgEncodeAwgTfbsSydhMcf10aesStat3Etoh01bUniPk	70411	17603
	wgEncodeAwgTfbsSydhMcf10aesStat3Etoh01cUniPk	64587	16147
	wgEncodeAwgTfbsSydhMcf10aesStat3Etoh01UniPk	19377	4845
	wgEncodeAwgTfbsSydhMcf10aesStat3Tam112hHvdUniPk	62265	15567
	wgEncodeAwgTfbsSydhMcf10aesStat3TamUniPk	69246	17312
TAF1	wgEncodeAwgTfbsHaibA549Taf1V0422111Etoh02UniPk	15691	3923
	wgEncodeAwgTfbsHaibGm12878Taf1Pcr1xUniPk	22305	5577
	wgEncodeAwgTfbsHaibGm12892Taf1V0416102UniPk	14776	3694
	wgEncodeAwgTfbsHaibHepg2Taf1Pcr2xUniPk	25369	6343
	wgEncodeAwgTfbsHaibK562Taf1V0416101UniPk	23428	5858
	wgEncodeAwgTfbsHaibSknshTaf1V0416101UniPk	21908	5478
TBPI	wgEncodeAwgTfbsSydhGm12878TbpIggmusUniPk	23278	5820
	wgEncodeAwgTfbsSydhHepg2TbpIggrabUniPk	21732	5434
	wgEncodeAwgTfbsSydhK562TbpIggmusUniPk	27345	6837
	wgEncodeAwgTfbsSydhH1hescCtbp2UcdUniPk	11228	2808
TEAD4	wgEncodeAwgTfbsHaibH1hescTead4sc101184V0422111UniPk	31233	7809
	wgEncodeAwgTfbsHaibHepg2Tead4sc101184V0422111UniPk	23852	5964
	wgEncodeAwgTfbsHaibK562Tead4sc101184V0422111UniPk	48372	12094
	wgEncodeAwgTfbsHaibGm12878Yy1sc281Pcr1xUniPk	48142	12036
	wgEncodeAwgTfbsHaibGm12892Yy1V0416101UniPk	24763	6191

YY1	wgEncodeAwgTfbsHaibH1hescYy1sc281V0416102UniPk	28672	7168
	wgEncodeAwgTfbsHaibHct116Yy1sc281V0416101UniPk	20043	5011
	wgEncodeAwgTfbsHaibHepg2Yy1sc281V0416101UniPk	27944	6986
	wgEncodeAwgTfbsHaibK562Yy1V0416101UniPk	19684	4922
	wgEncodeAwgTfbsHaibK562Yy1V0416102UniPk	37296	9324
	wgEncodeAwgTfbsHaibSknshraYy1sc281V0416102UniPk	24422	6106
	wgEncodeAwgTfbsSydhK562Yy1UcdUniPk	7638	1910
	wgEncodeAwgTfbsSydhNt2d1Yy1UcdUniPk	7465	1867
NFKB	wgEncodeAwgTfbsSydhGm10847NfkbTnfaIggrabUniPk	14545	3637
	wgEncodeAwgTfbsSydhGm12878NfkbTnfaIggrabUniPk	26700	6676
	wgEncodeAwgTfbsSydhGm12891NfkbTnfaIggrabUniPk	45032	11258
	wgEncodeAwgTfbsSydhGm12892NfkbTnfaIggrabUniPk	12865	3217
	wgEncodeAwgTfbsSydhGm18526NfkbTnfaIggrabUniPk	4820	1206
	wgEncodeAwgTfbsSydhGm19099NfkbTnfaIggrabUniPk	12190	3048
Pax5	wgEncodeAwgTfbsHaibGm12878Pax5c20Pcr1xUniPk	39841	9961
	wgEncodeAwgTfbsHaibGm12878Pax5n19Pcr1xUniPk	31012	7754
	wgEncodeAwgTfbsHaibGm12891Pax5c20V0416101UniPk	4979	1245
	wgEncodeAwgTfbsHaibGm12892Pax5c20V0416101UniPk	16284	4072
HDAC	wgEncodeAwgTfbsBroadK562Hdac2a300705aUniPk	8380	2096
	wgEncodeAwgTfbsBroadK562Hdac6a301341aUniPk	1779	445
	wgEncodeAwgTfbsHaibH1hescHdac2sc6296V0416102UniPk	8953	2239
	wgEncodeAwgTfbsHaibHepg2Hdac2sc6296V0416101UniPk	29865	7467
	wgEncodeAwgTfbsUchicagoK562Ehdac8UniPk	2734	684
	wgEncodeAwgTfbsHaibK562Hdac2sc6296V0416102UniPk	10539	2635
P300	wgEncodeAwgTfbsHaibGm12878P300Pcr1xUniPk	8052	2014
	wgEncodeAwgTfbsHaibHepg2P300V0416101UniPk	43721	10931
	wgEncodeAwgTfbsHaibSknshraP300V0416102UniPk	73086	18272
	wgEncodeAwgTfbsHaibT47dP300V0416102Dm002p1hUniPk	21969	5493
	wgEncodeAwgTfbsSydhGm12878P300bUniPk	9697	2425
	wgEncodeAwgTfbsSydhHelas3P300sc584sc584IggrabUniPk	40262	10066
	wgEncodeAwgTfbsSydhK562P300IggrabUniPk	40425	10107
ELF1	wgEncodeAwgTfbsHaibA549Elf1V0422111Etoh02UniPk	13468	3368
	wgEncodeAwgTfbsHaibGm12878Elf1sc631V0416101UniPk	35684	8922
	wgEncodeAwgTfbsHaibHepg2Elf1sc631V0416101UniPk	28315	7079
	wgEncodeAwgTfbsHaibK562Elf1sc631V0416102UniPk	43403	10851

Supplementary Table S2. The details of the convolution operations in our work

**Convolution operation**	**Parameter settings**	**Output shape**
**CNN module**		
$Conv_1$	kernel number = M_0, kernel size = (k_0, N), stride = S, padding = P_0	(Batchsize, M_0, d)
$Conv_{2,1}$, $Conv_{2,2}$, $Con_{v2,3}$	kernel number = M_1, kernel size = (k_0, M_0), stride = S, padding = P_0	(Batchsize, M_1, d)
$Conv_{2,4}$	kernel number = M_2, kernel size = (k_0, M_1), stride = S, padding = P_0	(Batchsize, M_2, d)
$Conv_{2,5}$	kernel number = M_2, kernel size = (k_0, M_1), stride = S, dilation = D_0 , padding = D_0*P_0	(Batchsize, M_2, d)
$Conv_{2,6}$	kernel number = M_2, kernel size = (k_0, M_1), stride = S, dilation = D_1 , padding = D_1*P_0	(Batchsize, M_2, d)
$Conv_3$	kernel number = M_3, kernel size = (k_0, 3*M_2), stride = S, padding = P_0	(Batchsize, M_3, d)
**Convolutional block attention module**		
$Conv_{4,1}$	kernel number = M_3 / r, kernel size = (1, M_3), stride = S, padding = P_1	(Batchsize, M_3 / r, 1)
$Conv_{4,2}$	kernel number = M_3, kernel size = (1, M_3 / r), stride = S, padding = P_1	(Batchsize, M_3, 1)
$Conv_5$	kernel number = 1, kernel size = (k_1, 2), stride = S, padding = P_0	(Batchsize, 1, d)
**Output module**		
$Conv_6$	kernel number = M_3, kernel size = (k_0, M_3), stride = S, padding = P_0	(Batchsize, M_3, d)

Supplementary Table S3. The fixed hyperparameters for the BERT-TFBS model.

**Hyperparameters**	**Values**
Word embedding dimension d	768
The number of self attention heads h	3
Number of encoders L	12
Kernel number M_0, M_1, M_2, M_3	60, 30, 60, 180
Kernel size k_0, k_1	3, 7
Stride S	1
Padding size P_0, P_1, P_2	1, 0, 3
Dilation ratio D_0, D_1	2, 4
Dimensionality reduction coefficient r	4
Batchsize	32

Supplementary Table S4. Prediction performances of BERT-TFBS model and its variant models.

Datasets	BERT-TFBS			BERT-TFBS-v1			BERT-TFBS-v2		
	Accuracy	ROC AUC	PR AUC	Accuracy	ROC AUC	PR AUC	Accuracy	ROC AUC	PR AUC
wgEncodeAwgTfbsBroadDnd41CtcfUniPk	0.925605	0.975698	0.977077	0.91864	0.952604	0.940049	0.922664	0.955283	0.958283
wgEncodeAwgTfbsBroadDnd41Ezh239875UniPk	0.71237	0.81264	0.828815	0.710879	0.765925	0.757952	0.704918	0.774225	0.800556
wgEncodeAwgTfbsBroadGm12878CtcfUniPk	0.917334	0.970701	0.972521	0.90996	0.951234	0.948612	0.911976	0.961011	0.960927
wgEncodeAwgTfbsBroadH1hescCtcfUniPk	0.920369	0.970095	0.963106	0.912111	0.944289	0.921454	0.917151	0.953265	0.947871
wgEncodeAwgTfbsBroadHelas3CtcfUniPk	0.904555	0.958292	0.959412	0.897054	0.929885	0.918529	0.899077	0.946095	0.943748
wgEncodeAwgTfbsBroadHelas3Ezh239875UniPk	0.712676	0.792691	0.814406	0.708451	0.740174	0.723257	0.664789	0.745565	0.77357
wgEncodeAwgTfbsBroadHelas3Pol2bUniPk	0.708787	0.792804	0.788981	0.704603	0.759556	0.723736	0.682845	0.761697	0.761427
wgEncodeAwgTfbsBroadHepg2CtcfUniPk	0.930207	0.976886	0.97585	0.923404	0.956161	0.942282	0.926833	0.966203	0.965135
wgEncodeAwgTfbsBroadHepg2Ezh239875UniPk	0.755539	0.827472	0.820838	0.735676	0.794309	0.768844	0.724981	0.803992	0.789823
wgEncodeAwgTfbsBroadHmecCtcfUniPk	0.939163	0.980906	0.981947	0.931525	0.959617	0.950311	0.937091	0.971018	0.974811
wgEncodeAwgTfbsBroadHsmmCtcfUniPk	0.924393	0.971673	0.968	0.917966	0.956117	0.947928	0.9208	0.951458	0.95185
wgEncodeAwgTfbsBroadHsmmEzh239875UniPk	0.720779	0.778484	0.755868	0.733766	0.782176	0.734003	0.685065	0.761747	0.742225
wgEncodeAwgTfbsBroadHsmmtCtcfUniPk	0.919256	0.970613	0.970014	0.91302	0.94962	0.941777	0.917805	0.961263	0.957326
wgEncodeAwgTfbsBroadHuvecCtcfUniPk	0.92985	0.976229	0.978232	0.924136	0.95625	0.948098	0.929162	0.965244	0.963881
wgEncodeAwgTfbsBroadHuvecEzh239875UniPk	0.762073	0.841551	0.848284	0.760306	0.804847	0.793271	0.748528	0.824592	0.817965
wgEncodeAwgTfbsBroadHuvecPol2bUniPk	0.757198	0.835992	0.826777	0.738004	0.795509	0.763018	0.740403	0.815188	0.800507
wgEncodeAwgTfbsBroadK562CtcfUniPk	0.894255	0.954492	0.957885	0.88769	0.930861	0.922648	0.893111	0.940403	0.93764
wgEncodeAwgTfbsBroadK562Ezh239875UniPk	0.688988	0.771991	0.753017	0.688988	0.722204	0.658473	0.690476	0.735363	0.733407
wgEncodeAwgTfbsBroadK562Hdac2a300705aUniPk	0.763359	0.848817	0.849864	0.750954	0.818964	0.803521	0.760019	0.830287	0.828886
wgEncodeAwgTfbsBroadK562Hdac6a301341aUniPk	0.680899	0.738479	0.740701	0.651685	0.701168	0.679517	0.683146	0.747575	0.764387
wgEncodeAwgTfbsBroadNhaCtcfUniPk	0.933351	0.9764	0.97725	0.9245	0.961101	0.956213	0.928121	0.969834	0.967086
wgEncodeAwgTfbsBroadNhdfadCtcfUniPk	0.930022	0.977562	0.977346	0.92016	0.95494	0.941143	0.922059	0.960773	0.955673
wgEncodeAwgTfbsBroadNhekCtcfUniPk	0.913866	0.968616	0.968471	0.908712	0.937813	0.922542	0.911837	0.944136	0.947121
wgEncodeAwgTfbsBroadNhekPol2bUniPk	0.720601	0.80589	0.820574	0.71176	0.764962	0.741224	0.714412	0.790214	0.802459
wgEncodeAwgTfbsBroadNhlfCtcfUniPk	0.918618	0.970683	0.97048	0.913009	0.956272	0.951349	0.919139	0.957925	0.958124
wgEncodeAwgTfbsBroadOsteoblCtcfUniPk	0.906723	0.961251	0.962021	0.898273	0.942373	0.938993	0.903891	0.951343	0.94669
wgEncodeAwgTfbsHaibA549Elf1V0422111Etoh02UniPk	0.842933	0.916547	0.914425	0.832542	0.874647	0.857118	0.832838	0.905495	0.90653
wgEncodeAwgTfbsHaibA549Fosl2V0422111Etoh02UniPk	0.903647	0.959742	0.962276	0.892206	0.942326	0.939004	0.896049	0.954289	0.956127
wgEncodeAwgTfbsHaibA549Foxa1V0416102Dex100nmUniPk	0.830458	0.913468	0.917163	0.830126	0.887775	0.871118	0.824154	0.900101	0.909048
wgEncodeAwgTfbsHaibA549GabpV0422111Etoh02UniPk	0.845163	0.91474	0.920244	0.818301	0.866754	0.845159	0.831186	0.898038	0.900372
wgEncodeAwgTfbsHaibA549Sin3ak20V0422111Etoh02UniPk	0.754622	0.831445	0.840855	0.729412	0.786583	0.756821	0.74958	0.817899	0.814984
wgEncodeAwgTfbsHaibA549Taf1V0422111Etoh02UniPk	0.745093	0.841107	0.850122	0.730818	0.784941	0.75092	0.744073	0.820259	0.810278
wgEncodeAwgTfbsHaibEcc1Foxa1sc6553V0416102Dm002p1hUniPk	0.855235	0.919757	0.922099	0.824645	0.875893	0.84851	0.828522	0.893228	0.878962
wgEncodeAwgTfbsHaibGm12878Elf1sc631V0416101UniPk	0.839946	0.916929	0.914967	0.833782	0.890049	0.877668	0.838937	0.902883	0.892325
wgEncodeAwgTfbsHaibGm12878GabpPcr2xUniPk	0.860165	0.919213	0.926604	0.851156	0.910655	0.912675	0.853114	0.914175	0.918636
wgEncodeAwgTfbsHaibGm12878P300Pcr1xUniPk	0.779047	0.874111	0.883469	0.76862	0.825151	0.809739	0.777557	0.857376	0.861406
wgEncodeAwgTfbsHaibGm12878Pax5c20Pcr1xUniPk	0.825821	0.90651	0.908648	0.814276	0.863122	0.834825	0.816886	0.888937	0.872886
wgEncodeAwgTfbsHaibGm12878Pax5n19Pcr1xUniPk	0.822414	0.905953	0.903428	0.80926	0.860628	0.834752	0.821511	0.890935	0.888006
wgEncodeAwgTfbsHaibGm12878Taf1Pcr1xUniPk	0.802044	0.875397	0.870511	0.788955	0.846303	0.832365	0.793437	0.858328	0.837418
wgEncodeAwgTfbsHaibGm12878Yy1sc281Pcr1xUniPk	0.866982	0.93397	0.936484	0.851363	0.906283	0.895835	0.860003	0.923576	0.923401
wgEncodeAwgTfbsHaibGm12891Pax5c20V0416101UniPk	0.7751	0.851853	0.854835	0.734137	0.784584	0.768631	0.771888	0.849414	0.853176
wgEncodeAwgTfbsHaibGm12892Pax5c20V0416101UniPk	0.792976	0.87776	0.881869	0.788556	0.845558	0.837263	0.784627	0.861437	0.863079
wgEncodeAwgTfbsHaibGm12892Taf1V0416102UniPk	0.78993	0.871945	0.874153	0.77477	0.833311	0.811717	0.786952	0.857023	0.852067
wgEncodeAwgTfbsHaibGm12892Yy1V0416101UniPk	0.860443	0.932004	0.937475	0.84849	0.909911	0.909743	0.850428	0.920258	0.9207
wgEncodeAwgTfbsHaibH1hescFosl1sc183V0416102UniPk	0.784091	0.853051	0.874542	0.736364	0.799438	0.811814	0.754545	0.828022	0.858023
wgEncodeAwgTfbsHaibH1hescGabpPcr1xUniPk	0.849242	0.92547	0.92835	0.837645	0.896931	0.881381	0.843889	0.919085	0.91684
wgEncodeAwgTfbsHaibH1hescHdac2sc6296V0416102UniPk	0.800804	0.871959	0.865771	0.786512	0.849753	0.838863	0.798124	0.865841	0.864357
wgEncodeAwgTfbsHaibH1hescSp1Pcr1xUniPk	0.823579	0.904158	0.909263	0.807679	0.86835	0.861425	0.821042	0.885934	0.884816
wgEncodeAwgTfbsHaibH1hescSp2V0422111UniPk	0.861169	0.935528	0.938152	0.852818	0.928592	0.937024	0.862213	0.926888	0.92121
wgEncodeAwgTfbsHaibH1hescSrfPcr1xUniPk	0.841238	0.917006	0.920797	0.811782	0.87174	0.859932	0.830754	0.908019	0.908958
wgEncodeAwgTfbsHaibH1hescTead4sc101184V0422111UniPk	0.911768	0.965915	0.964972	0.897938	0.948015	0.943949	0.901012	0.959116	0.957305
wgEncodeAwgTfbsHaibH1hescYy1sc281V0416102UniPk	0.868025	0.942082	0.94696	0.855469	0.918098	0.917253	0.860491	0.931307	0.937998
wgEncodeAwgTfbsHaibHct116Yy1sc281V0416101UniPk	0.850928	0.928165	0.934134	0.834165	0.898199	0.890833	0.844342	0.912705	0.917309
wgEncodeAwgTfbsHaibHelas3GabpPcr1xUniPk	0.872713	0.940965	0.945845	0.863948	0.916494	0.912441	0.870808	0.936839	0.941297
wgEncodeAwgTfbsHaibHepg2Cebpbsc150V0416101UniPk	0.925734	0.977058	0.977443	0.896532	0.95348	0.948518	0.911414	0.968673	0.968018
wgEncodeAwgTfbsHaibHepg2Elf1sc631V0416101UniPk	0.887131	0.95635	0.956488	0.872157	0.926125	0.910494	0.87922	0.93836	0.933407
wgEncodeAwgTfbsHaibHepg2Fosl2V0416101UniPk	0.887097	0.949181	0.953942	0.872379	0.915874	0.903426	0.878427	0.938604	0.941641
wgEncodeAwgTfbsHaibHepg2Foxa1sc101058V0416101UniPk	0.903482	0.961759	0.958508	0.891639	0.938555	0.92632	0.898271	0.956655	0.951332
wgEncodeAwgTfbsHaibHepg2Foxa1sc6553V0416101UniPk	0.912096	0.966346	0.962382	0.89634	0.935424	0.919478	0.905215	0.957854	0.954229
wgEncodeAwgTfbsHaibHepg2GabpPcr2xUniPk	0.864817	0.922175	0.925717	0.851578	0.909348	0.904556	0.857179	0.914611	0.91911
wgEncodeAwgTfbsHaibHepg2Hdac2sc6296V0416101UniPk	0.831659	0.902595	0.902116	0.807018	0.859785	0.838264	0.811705	0.887397	0.877838
wgEncodeAwgTfbsHaibHepg2JundPcr1xUniPk	0.863285	0.938747	0.943001	0.856373	0.897274	0.889845	0.860473	0.928374	0.931391
wgEncodeAwgTfbsHaibHepg2P300V0416101UniPk	0.856097	0.928774	0.92771	0.834965	0.899209	0.887426	0.846034	0.916846	0.904767
wgEncodeAwgTfbsHaibHepg2Sin3ak20Pcr1xUniPk	0.795486	0.872153	0.874272	0.77829	0.842014	0.837318	0.791033	0.850897	0.845469
wgEncodeAwgTfbsHaibHepg2Sp1Pcr1xUniPk	0.851422	0.930698	0.934869	0.836604	0.889106	0.876922	0.839608	0.91456	0.91062
wgEncodeAwgTfbsHaibHepg2Sp2V0422111UniPk	0.852601	0.921895	0.926248	0.834297	0.886262	0.883167	0.847784	0.920882	0.924784
wgEncodeAwgTfbsHaibHepg2SrfV0416101UniPk	0.840019	0.920324	0.925232	0.80659	0.873667	0.844661	0.817096	0.889958	0.881216
wgEncodeAwgTfbsHaibHepg2Taf1Pcr2xUniPk	0.798518	0.880408	0.873768	0.786221	0.852564	0.830761	0.791266	0.872228	0.863188
wgEncodeAwgTfbsHaibHepg2Tead4sc101184V0422111UniPk	0.830651	0.913606	0.920998	0.819081	0.867764	0.847649	0.830651	0.900863	0.903159
wgEncodeAwgTfbsHaibHepg2Yy1sc281V0416101UniPk	0.831091	0.910403	0.910041	0.824936	0.880594	0.867061	0.830948	0.894494	0.890624
wgEncodeAwgTfbsHaibK562Cebpbsc150V0422111UniPk	0.895936	0.957432	0.959448	0.878784	0.930594	0.922117	0.883619	0.9469	0.947424
wgEncodeAwgTfbsHaibK562Elf1sc631V0416102UniPk	0.902313	0.960058	0.958854	0.889595	0.92832	0.909816	0.896323	0.949461	0.941951
wgEncodeAwgTfbsHaibK562Fosl1sc183V0416101UniPk	0.921466	0.972276	0.97524	0.90667	0.946902	0.944726	0.918279	0.961867	0.965852
wgEncodeAwgTfbsHaibK562GabpV0416101UniPk	0.863141	0.932178	0.929402	0.8526	0.912877	0.90169	0.854029	0.915522	0.914173
wgEncodeAwgTfbsHaibK562Gata2sc267Pcr1xUniPk	0.89177	0.956611	0.956444	0.865975	0.912668	0.889074	0.878075	0.946991	0.94362
wgEncodeAwgTfbsHaibK562Hdac2sc6296V0416102UniPk	0.793169	0.890635	0.88814	0.780645	0.844727	0.820119	0.798861	0.870598	0.847079
wgEncodeAwgTfbsHaibK562MaxV0416102UniPk	0.841734	0.920317	0.920159	0.837121	0.894553	0.884855	0.838089	0.911888	0.910331
wgEncodeAwgTfbsHaibK562Sin3ak20V0416101UniPk	0.79836	0.880304	0.881595	0.780156	0.835508	0.802949	0.789758	0.864144	0.865791
wgEncodeAwgTfbsHaibK562SrfV0416101UniPk	0.79288	0.892926	0.89637	0.81014	0.86044	0.847278	0.824703	0.885969	0.891774
wgEncodeAwgTfbsHaibK562Taf1V0416101UniPk	0.808638	0.888035	0.891368	0.793786	0.851352	0.838314	0.79942	0.870508	0.870528
wgEncodeAwgTfbsHaibK562Tead4sc101184V0422111UniPk	0.859517	0.930535	0.927033	0.846287	0.897317	0.886702	0.848272	0.921825	0.917429
wgEncodeAwgTfbsHaibK562Yy1V0416101UniPk	0.852093	0.92838	0.925621	0.845794	0.904313	0.887322	0.844982	0.912005	0.906944
wgEncodeAwgTfbsHaibK562Yy1V0416102UniPk	0.864972	0.938105	0.939851	0.85607	0.90586	0.886349	0.860789	0.925845	0.925778
wgEncodeAwgTfbsHaibPanc1Sin3ak20V0416101UniPk	0.760184	0.860587	0.857475	0.761601	0.831417	0.809103	0.767623	0.842754	0.835822
wgEncodeAwgTfbsHaibSknshraP300V0416102UniPk	0.817261	0.905251	0.896469	0.823063	0.87721	0.852159	0.823446	0.896348	0.882881
wgEncodeAwgTfbsHaibSknshraYy1sc281V0416102UniPk	0.842123	0.917852	0.922469	0.83115	0.888739	0.884447	0.834916	0.902926	0.899292
wgEncodeAwgTfbsHaibSknshTaf1V0416101UniPk	0.804491	0.89051	0.894011	0.79226	0.855572	0.82076	0.798284	0.875845	0.862699
wgEncodeAwgTfbsHaibT47dFoxa1sc6553V0416102Dm002p1hUniPk	0.897395	0.95841	0.958164	0.881643	0.923836	0.906057	0.888998	0.948041	0.945021
wgEncodeAwgTfbsHaibT47dGata3sc268V0416102Dm002p1hUniPk	0.834363	0.919172	0.920464	0.817963	0.868883	0.845833	0.828599	0.901601	0.892137
wgEncodeAwgTfbsHaibT47dP300V0416102Dm002p1hUniPk	0.815766	0.894051	0.893907	0.789914	0.844015	0.822551	0.792099	0.870939	0.860408
wgEncodeAwgTfbsSydhA549CebpbIggrabUniPk	0.943241	0.984178	0.982469	0.932311	0.97875	0.980013	0.936525	0.979359	0.975769
wgEncodeAwgTfbsSydhGm10847NfkbTnfaIggrabUniPk	0.819082	0.896726	0.901912	0.80011	0.868383	0.86937	0.804234	0.882642	0.894239

wgEncodeAwgTfbsSydhGm12878CfosUniPk	0.941514	0.966907	0.974225	0.930046	0.963274	0.965484	0.938073	0.969674	0.964832
wgEncodeAwgTfbsSydhGm12878JundUniPk	0.853183	0.930888	0.927301	0.800821	0.856359	0.816065	0.835729	0.909131	0.907376
wgEncodeAwgTfbsSydhGm12878MaxIggmusUniPk	0.802917	0.885254	0.887174	0.792992	0.849502	0.827335	0.793599	0.867532	0.863041
wgEncodeAwgTfbsSydhGm12878NfkbTnfaIggrabUniPk	0.834332	0.91182	0.912305	0.818904	0.865101	0.838057	0.822948	0.888923	0.883432
wgEncodeAwgTfbsSydhGm12878P300bUniPk	0.843711	0.919253	0.921914	0.818969	0.873172	0.83868	0.827629	0.895112	0.883063
wgEncodeAwgTfbsSydhGm12878Stat3IggmusUniPk	0.756969	0.841552	0.838043	0.73616	0.801406	0.769736	0.750687	0.819942	0.802277
wgEncodeAwgTfbsSydhGm12878TbpIggmusUniPk	0.79433	0.868193	0.873572	0.774399	0.824948	0.778342	0.776976	0.852955	0.852895
wgEncodeAwgTfbsSydhGm12891NfkbTnfaIggrabUniPk	0.843045	0.920013	0.92008	0.826079	0.882547	0.862716	0.835584	0.90349	0.895725
wgEncodeAwgTfbsSydhGm12892NfkbTnfaIggrabUniPk	0.829344	0.908848	0.917132	0.804476	0.862705	0.844969	0.819708	0.885669	0.8928
wgEncodeAwgTfbsSydhGm18526NfkbTnfaIggrabUniPk	0.817579	0.888158	0.898821	0.799337	0.850966	0.843247	0.803483	0.874477	0.884593
wgEncodeAwgTfbsSydhGm19099NfkbTnfaIggrabUniPk	0.845472	0.918752	0.921293	0.8271	0.885105	0.869996	0.835958	0.909301	0.907125
wgEncodeAwgTfbsSydhH1hescCebpbIggrabUniPk	0.946878	0.986226	0.985293	0.929225	0.972511	0.971358	0.935273	0.977064	0.9786
wgEncodeAwgTfbsSydhH1hescCtbp2UcdUniPk	0.757479	0.843157	0.846364	0.743234	0.801671	0.76813	0.75	0.820018	0.819141
wgEncodeAwgTfbsSydhH1hescJundIggrabUniPk	0.874567	0.939946	0.946541	0.856991	0.915238	0.916817	0.861252	0.922314	0.933833
wgEncodeAwgTfbsSydhH1hescMaxUcdUniPk	0.857143	0.931214	0.936514	0.851675	0.90502	0.902171	0.852358	0.919363	0.923869
wgEncodeAwgTfbsSydhH1hescSin3anb6001263IggrabUniPk	0.801068	0.883439	0.888122	0.788368	0.847782	0.823106	0.793947	0.873147	0.86729
wgEncodeAwgTfbsSydhHelas3CebpbIggrabUniPk	0.906424	0.962816	0.960849	0.891755	0.93546	0.915358	0.897994	0.954978	0.946427
wgEncodeAwgTfbsSydhHelas3CfosUniPk	0.913907	0.963001	0.965342	0.898731	0.939993	0.932489	0.905905	0.954804	0.958863
wgEncodeAwgTfbsSydhHelas3CjunIggrabUniPk	0.888185	0.954618	0.958243	0.877625	0.933328	0.934097	0.882436	0.945435	0.94726
wgEncodeAwgTfbsSydhHelas3Hae2f1UniPk	0.827164	0.908472	0.909758	0.818659	0.882908	0.863726	0.822661	0.892982	0.886857
wgEncodeAwgTfbsSydhHelas3JundIggrabUniPk	0.919031	0.968686	0.970407	0.907325	0.943092	0.941703	0.915698	0.962457	0.962206
wgEncodeAwgTfbsSydhHelas3MaxIggrabUniPk	0.824634	0.891506	0.877766	0.812516	0.868353	0.849109	0.820739	0.891227	0.880532
wgEncodeAwgTfbsSydhHelas3P300sc584sc584IggrabUniPk	0.856646	0.936603	0.938036	0.831015	0.883116	0.862618	0.846513	0.922034	0.916387
wgEncodeAwgTfbsSydhHelas3Stat3IggrabUniPk	0.815062	0.90588	0.914029	0.803561	0.840645	0.813006	0.818401	0.895808	0.9016
wgEncodeAwgTfbsSydhHepg2CebpbForsklnUniPk	0.916184	0.97152	0.97322	0.887372	0.945424	0.945516	0.895092	0.953496	0.949939
wgEncodeAwgTfbsSydhHepg2CebpbIggrabUniPk	0.951703	0.985224	0.978533	0.94138	0.973937	0.96059	0.947619	0.981641	0.97745
wgEncodeAwgTfbsSydhHepg2CjunIggrabUniPk	0.924351	0.974109	0.977108	0.917964	0.959499	0.960365	0.923752	0.969673	0.974224
wgEncodeAwgTfbsSydhHepg2Corestsc30189IggrabUniPk	0.778769	0.857508	0.866415	0.770784	0.836607	0.822592	0.775012	0.843001	0.844553
wgEncodeAwgTfbsSydhHepg2JundIggrabUniPk	0.945676	0.983202	0.984095	0.938836	0.973119	0.968826	0.943632	0.978965	0.981473
wgEncodeAwgTfbsSydhHepg2MaxIggrabUniPk	0.833617	0.911957	0.913162	0.830209	0.877163	0.856918	0.82467	0.895626	0.89412
wgEncodeAwgTfbsSydhHepg2TbpIggrabUniPk	0.800147	0.877764	0.883525	0.782481	0.842787	0.825122	0.784505	0.864116	0.862289
wgEncodeAwgTfbsSydhHuvecCfosUcdUniPk	0.913217	0.963661	0.963789	0.900773	0.942654	0.934243	0.905268	0.954512	0.95634
wgEncodeAwgTfbsSydhHuvecCjunUniPk	0.922043	0.974037	0.973365	0.906868	0.940478	0.926679	0.916493	0.968099	0.963778
wgEncodeAwgTfbsSydhHuvecGata2UcdUniPk	0.845188	0.918565	0.919897	0.822603	0.868012	0.840795	0.830381	0.906782	0.903976
wgEncodeAwgTfbsSydhHuvecMaxUniPk	0.86539	0.951476	0.952211	0.877324	0.923518	0.916322	0.881488	0.941959	0.944333
wgEncodeAwgTfbsSydhImr90CebpbIggrabUniPk	0.931056	0.976488	0.971654	0.919389	0.956267	0.937308	0.923691	0.971403	0.965741
wgEncodeAwgTfbsSydhK562CebpbIggrabUniPk	0.941472	0.981732	0.979552	0.92651	0.964657	0.957922	0.931541	0.975238	0.971707
wgEncodeAwgTfbsSydhK562CfosUniPk	0.948195	0.975761	0.977324	0.939505	0.965921	0.955779	0.942513	0.974131	0.975036
wgEncodeAwgTfbsSydhK562CjunUniPk	0.922142	0.971903	0.972091	0.898603	0.942788	0.932333	0.912054	0.964626	0.965313
wgEncodeAwgTfbsSydhK562Corestsc30189IggrabUniPk	0.865438	0.937759	0.938856	0.849854	0.908836	0.896731	0.859318	0.921927	0.908474
wgEncodeAwgTfbsSydhK562Gata2UcdUniPk	0.860197	0.941317	0.938789	0.836416	0.886246	0.872117	0.857074	0.929142	0.934467
wgEncodeAwgTfbsSydhK562JundIggrabUniPk	0.89158	0.954805	0.956116	0.878259	0.92634	0.92109	0.889732	0.946193	0.940105
wgEncodeAwgTfbsSydhK562MaxIggrabUniPk	0.839351	0.908172	0.900094	0.828641	0.880613	0.862516	0.836511	0.905228	0.899831
wgEncodeAwgTfbsSydhK562P300IggrabUniPk	0.855941	0.926001	0.920323	0.837143	0.889876	0.864082	0.851489	0.92291	0.918527
wgEncodeAwgTfbsSydhK562TbpIggmusUniPk	0.810443	0.891695	0.901177	0.805909	0.86039	0.847407	0.805031	0.878378	0.879165
wgEncodeAwgTfbsSydhK562Yy1UcdUniPk	0.902618	0.96755	0.968769	0.9	0.959047	0.96621	0.909948	0.95895	0.961203
wgEncodeAwgTfbsSydhMcf10aesCfosTam112hHvdUniPk	0.929303	0.97678	0.974712	0.920211	0.953246	0.931629	0.925454	0.971924	0.96415
wgEncodeAwgTfbsSydhMcf10aesCfosTam14hHvdUniPk	0.919332	0.971512	0.967123	0.909845	0.943961	0.920651	0.915453	0.964627	0.954105
wgEncodeAwgTfbsSydhMcf10aesCfosTamHvdUniPk	0.931772	0.978136	0.975526	0.923001	0.946974	0.929055	0.928008	0.971866	0.963216
wgEncodeAwgTfbsSydhMcf10aesStat3Etoh01bUniPk	0.896495	0.960031	0.958729	0.879452	0.923433	0.911015	0.885588	0.948059	0.939643
wgEncodeAwgTfbsSydhMcf10aesStat3Etoh01cUniPk	0.898619	0.960807	0.960284	0.882021	0.925643	0.906353	0.887347	0.949082	0.940379
wgEncodeAwgTfbsSydhMcf10aesStat3Etoh01UniPk	0.854283	0.933254	0.926565	0.840041	0.890921	0.873477	0.849536	0.920424	0.917412
wgEncodeAwgTfbsSydhMcf10aesStat3Tam112hHvdUniPk	0.895484	0.958425	0.956783	0.87936	0.917342	0.895745	0.890216	0.946921	0.939006
wgEncodeAwgTfbsSydhMcf10aesStat3TamUniPk	0.90342	0.963494	0.961144	0.88765	0.925873	0.908779	0.894755	0.95393	0.944954
wgEncodeAwgTfbsSydhMcf7Gata3UcdUniPk	0.776804	0.864635	0.872642	0.75219	0.813205	0.797363	0.770547	0.84413	0.850749
wgEncodeAwgTfbsSydhMcf7Hae2f1UcdUniPk	0.799114	0.877357	0.880788	0.790255	0.843552	0.829443	0.794424	0.8631	0.85663
wgEncodeAwgTfbsSydhNb4CmycUniPk	0.821681	0.905476	0.910004	0.811403	0.867476	0.854036	0.819354	0.893878	0.888835
wgEncodeAwgTfbsSydhNb4MaxUniPk	0.844159	0.925973	0.920227	0.844893	0.896221	0.880074	0.849008	0.91522	0.906528
wgEncodeAwgTfbsSydhNt2d1Yy1UcdUniPk	0.891805	0.950319	0.958317	0.876808	0.930257	0.934603	0.888591	0.936046	0.931081
wgEncodeAwgTfbsSydhShsy5yGata2UcdUniPk	0.844751	0.923805	0.920898	0.828733	0.880523	0.859229	0.840174	0.913288	0.904429
wgEncodeAwgTfbsSydhShsy5yGata3sc269sc269UcdUniPk	0.821577	0.90323	0.907885	0.799936	0.854816	0.837031	0.805547	0.878519	0.857331
wgEncodeAwgTfbsUchicagoK562EfosUniPk	0.932506	0.976052	0.978727	0.920596	0.959409	0.9594	0.927047	0.970652	0.97458
wgEncodeAwgTfbsUchicagoK562Egata2UniPk	0.803865	0.880355	0.882897	0.786317	0.837554	0.806433	0.794758	0.870904	0.871134
wgEncodeAwgTfbsUchicagoK562Ehdac8UniPk	0.673977	0.746772	0.760763	0.627193	0.675513	0.651376	0.641813	0.697227	0.705226
wgEncodeAwgTfbsUchicagoK562EjundUniPk	0.872067	0.938962	0.943464	0.857799	0.905457	0.898001	0.863449	0.928719	0.932437
wgEncodeAwgTfbsUtaGm12878CmycUniPk	0.753772	0.827633	0.832818	0.738683	0.795629	0.79216	0.731824	0.816594	0.819581
wgEncodeAwgTfbsUtaGm12878CtcfUniPk	0.933383	0.9783	0.976124	0.930311	0.961317	0.952213	0.934548	0.973521	0.971899
wgEncodeAwgTfbsUtaH1hescCmycUniPk	0.696907	0.782481	0.80042	0.703093	0.724917	0.689066	0.723711	0.779114	0.786724
wgEncodeAwgTfbsUtaH1hescCtcfUniPk	0.919413	0.973036	0.972458	0.910834	0.95128	0.941205	0.913141	0.958636	0.957654
wgEncodeAwgTfbsUtaHelas3CtcfUniPk	0.937767	0.979761	0.974369	0.931561	0.960654	0.946269	0.937107	0.967634	0.962432
wgEncodeAwgTfbsUtaHepg2CmycUniPk	0.807868	0.881747	0.881165	0.800456	0.853576	0.827883	0.798746	0.880122	0.878
wgEncodeAwgTfbsUtaK562CtcfUniPk	0.938347	0.981014	0.978712	0.931527	0.961861	0.947994	0.937656	0.972631	0.964485
wgEncodeAwgTfbsUtaMcf7CtcfUniPk	0.905832	0.965185	0.961933	0.900058	0.938899	0.919992	0.904722	0.947563	0.949577
Average	0.851013	0.919289	0.920609	0.837822	0.887371	0.872335	0.84399	0.906176	0.904126

Supplementary Table S5. The cell lines for the 165 ChIP-seq datasets.

Cell lines	TFs	Datasets
HelaS3	CTCF	wgEncodeAwgTfbsBroadHelas3CtcfUniPk
	EZH2	wgEncodeAwgTfbsBroadHelas3Ezh239875UniPk
	Pol2b	wgEncodeAwgTfbsBroadHelas3Pol2bUniPk
	GABP	wgEncodeAwgTfbsHaibHelas3GabpPcr1xUniPk
	CEBPB	wgEncodeAwgTfbsSydhHelas3CebpbIggrabUniPk
	FOS	wgEncodeAwgTfbsSydhHelas3CfosUniPk
	JUN	wgEncodeAwgTfbsSydhHelas3CjunIggrabUniPk
	HaE2F1	wgEncodeAwgTfbsSydhHelas3Hae2f1UniPk
	JUND	wgEncodeAwgTfbsSydhHelas3JundIggrabUniPk
	MAX	wgEncodeAwgTfbsSydhHelas3MaxIggrabUniPk
	P300	wgEncodeAwgTfbsSydhHelas3P300sc584sc584IggrabUniPk
	STAT3	wgEncodeAwgTfbsSydhHelas3Stat3IggrabUniPk
	CTCF	wgEncodeAwgTfbsUtaHelas3CtcfUniPk
HepG2	CTCF	wgEncodeAwgTfbsBroadHepg2CtcfUniPk
	EZH2	wgEncodeAwgTfbsBroadHepg2Ezh239875UniPk
	CEBPB	wgEncodeAwgTfbsHaibHepg2Cebpbsc150V0416101UniPk
	ELF1	wgEncodeAwgTfbsHaibHepg2Elf1sc631V0416101UniPk
	FOS	wgEncodeAwgTfbsHaibHepg2Fosl2V0416101UniPk
	FOXA1	wgEncodeAwgTfbsHaibHepg2Foxa1sc101058V0416101UniPk
	FOXA1	wgEncodeAwgTfbsHaibHepg2Foxa1sc6553V0416101UniPk
	GABP	wgEncodeAwgTfbsHaibHepg2GabpPcr2xUniPk
	HDAC	wgEncodeAwgTfbsHaibHepg2Hdac2sc6296V0416101UniPk
	JUND	wgEncodeAwgTfbsHaibHepg2JundPcr1xUniPk
	P300	wgEncodeAwgTfbsHaibHepg2P300V0416101UniPk
	Sin3A	wgEncodeAwgTfbsHaibHepg2Sin3ak20Pcr1xUniPk
	SP1	wgEncodeAwgTfbsHaibHepg2Sp1Pcr1xUniPk
	SP2	wgEncodeAwgTfbsHaibHepg2Sp2V0422111UniPk
	SRF	wgEncodeAwgTfbsHaibHepg2SrfV0416101UniPk
	TAF1	wgEncodeAwgTfbsHaibHepg2Taf1Pcr2xUniPk
	TEAD4	wgEncodeAwgTfbsHaibHepg2Tead4sc101184V0422111UniPk
	YY1	wgEncodeAwgTfbsHaibHepg2Yy1sc281V0416101UniPk
	CEBPB	wgEncodeAwgTfbsSydhHepg2CebpbForsklnUniPk
	CEBPB	wgEncodeAwgTfbsSydhHepg2CebpbIggrabUniPk
	JUN	wgEncodeAwgTfbsSydhHepg2CjunIggrabUniPk
	CoREST	wgEncodeAwgTfbsSydhHepg2Corestsc30189IggrabUniPk
	JUND	wgEncodeAwgTfbsSydhHepg2JundIggrabUniPk
	MAX	wgEncodeAwgTfbsSydhHepg2MaxIggrabUniPk
	TBPI	wgEncodeAwgTfbsSydhHepg2TbpIggrabUniPk
	MYC	wgEncodeAwgTfbsUtaHepg2CmycUniPk
Gm12878	CTCF	wgEncodeAwgTfbsBroadGm12878CtcfUniPk
	ELF1	wgEncodeAwgTfbsHaibGm12878Elf1sc631V0416101UniPk
	GABP	wgEncodeAwgTfbsHaibGm12878GabpPcr2xUniPk
	P300	wgEncodeAwgTfbsHaibGm12878P300Pcr1xUniPk
	Pax5	wgEncodeAwgTfbsHaibGm12878Pax5c20Pcr1xUniPk
	Pax5	wgEncodeAwgTfbsHaibGm12878Pax5n19Pcr1xUniPk
	TAF1	wgEncodeAwgTfbsHaibGm12878Taf1Pcr1xUniPk
	YY1	wgEncodeAwgTfbsHaibGm12878Yy1sc281Pcr1xUniPk
	FOS	wgEncodeAwgTfbsSydhGm12878CfosUniPk
	JUND	wgEncodeAwgTfbsSydhGm12878JundUniPk
	MAX	wgEncodeAwgTfbsSydhGm12878MaxIggmusUniPk
	NFKB	wgEncodeAwgTfbsSydhGm12878NfkbTnfaIggrabUniPk
	P300	wgEncodeAwgTfbsSydhGm12878P300bUniPk
	STAT3	wgEncodeAwgTfbsSydhGm12878Stat3IggmusUniPk
	TBPI	wgEncodeAwgTfbsSydhGm12878TbpIggmusUniPk
	MYC	wgEncodeAwgTfbsUtaGm12878CmycUniPk
	CTCF	wgEncodeAwgTfbsUtaGm12878CtcfUniPk

	CTCF	wgEncodeAwgTfbsBroadK562CtcfUniPk
	EZH2	wgEncodeAwgTfbsBroadK562Ezh239875UniPk
	HDAC	wgEncodeAwgTfbsBroadK562Hdac2a300705aUniPk
	HDAC	wgEncodeAwgTfbsBroadK562Hdac6a301341aUniPk
	CEBPB	wgEncodeAwgTfbsHaibK562Cebpbsc150V0422111UniPk
	ELF1	wgEncodeAwgTfbsHaibK562Elf1sc631V0416102UniPk
	FOS	wgEncodeAwgTfbsHaibK562Fosl1sc183V0416101UniPk
	GABP	wgEncodeAwgTfbsHaibK562GabpV0416101UniPk
	GATA2	wgEncodeAwgTfbsHaibK562Gata2sc267Pcr1xUniPk
	HDAC	wgEncodeAwgTfbsHaibK562Hdac2sc6296V0416102UniPk
	MAX	wgEncodeAwgTfbsHaibK562MaxV0416102UniPk
	Sin3A	wgEncodeAwgTfbsHaibK562Sin3ak20V0416101UniPk
	SRF	wgEncodeAwgTfbsHaibK562SrfV0416101UniPk
	TAF1	wgEncodeAwgTfbsHaibK562Taf1V0416101UniPk
	TEAD4	wgEncodeAwgTfbsHaibK562Tead4sc101184V0422111UniPk
	YY1	wgEncodeAwgTfbsHaibK562Yy1V0416101UniPk
K562	YY1	wgEncodeAwgTfbsHaibK562Yy1V0416102UniPk
	CEBPB	wgEncodeAwgTfbsSydhK562CebpbIggrabUniPk
	FOS	wgEncodeAwgTfbsSydhK562CfosUniPk
	JUN	wgEncodeAwgTfbsSydhK562CjunUniPk
	CoREST	wgEncodeAwgTfbsSydhK562Corestsc30189IggrabUniPk
	GATA2	wgEncodeAwgTfbsSydhK562Gata2UcdUniPk
	JUND	wgEncodeAwgTfbsSydhK562JundIggrabUniPk
	MAX	wgEncodeAwgTfbsSydhK562MaxIggrabUniPk
	P300	wgEncodeAwgTfbsSydhK562P300IggrabUniPk
	TBPI	wgEncodeAwgTfbsSydhK562TbpIggmusUniPk
	YY1	wgEncodeAwgTfbsSydhK562Yy1UcdUniPk
	FOS	wgEncodeAwgTfbsUchicagoK562EfosUniPk
	GATA2	wgEncodeAwgTfbsUchicagoK562Egata2UniPk
	HDAC	wgEncodeAwgTfbsUchicagoK562Ehdac8UniPk
	JUND	wgEncodeAwgTfbsUchicagoK562EjundUniPk
	CTCF	wgEncodeAwgTfbsUtaK562CtcfUniPk
	ELF1	wgEncodeAwgTfbsHaibA549Elf1V0422111Etoh02UniPk
	FOS	wgEncodeAwgTfbsHaibA549Fosl2V0422111Etoh02UniPk
	FOXA1	wgEncodeAwgTfbsHaibA549Foxa1V0416102Dex100nmUniPk
A549	GABP	wgEncodeAwgTfbsHaibA549GabpV0422111Etoh02UniPk
	Sin3A	wgEncodeAwgTfbsHaibA549Sin3ak20V0422111Etoh02UniPk
	TAF1	wgEncodeAwgTfbsHaibA549Taf1V0422111Etoh02UniPk
	CEBPB	wgEncodeAwgTfbsSydhA549CebpbIggrabUniPk
	CTCF	wgEncodeAwgTfbsBroadH1hescCtcfUniPk
	FOS	wgEncodeAwgTfbsHaibH1hescFosl1sc183V0416102UniPk
	GABP	wgEncodeAwgTfbsHaibH1hescGabpPcr1xUniPk
	HDAC	wgEncodeAwgTfbsHaibH1hescHdac2sc6296V0416102UniPk
	SP1	wgEncodeAwgTfbsHaibH1hescSp1Pcr1xUniPk
	SP2	wgEncodeAwgTfbsHaibH1hescSp2V0422111UniPk
	SRF	wgEncodeAwgTfbsHaibH1hescSrfPcr1xUniPk
	TEAD4	wgEncodeAwgTfbsHaibH1hescTead4sc101184V0422111UniPk
H1hesc	YY1	wgEncodeAwgTfbsHaibH1hescYy1sc281V0416102UniPk
	CEBPB	wgEncodeAwgTfbsSydhH1hescCebpbIggrabUniPk
	TBPI	wgEncodeAwgTfbsSydhH1hescCtbp2UcdUniPk
	JUND	wgEncodeAwgTfbsSydhH1hescJundIggrabUniPk
	MAX	wgEncodeAwgTfbsSydhH1hescMaxUcdUniPk
	Sin3A	wgEncodeAwgTfbsSydhH1hescSin3anb6001263IggrabUniPk
	MYC	wgEncodeAwgTfbsUtaH1hescCmycUniPk
	CTCF	wgEncodeAwgTfbsUtaH1hescCtcfUniPk
IMR90	CEBPB	wgEncodeAwgTfbsSydhImr90CebpbIggrabUniPk
Dnd41	CTCF	wgEncodeAwgTfbsBroadDnd41CtcfUniPk
	EZH2	wgEncodeAwgTfbsBroadDnd41Ezh239875UniPk

Hmec	CTCF	wgEncodeAwgTfbsBroadHmecCtcfUniPk
Hsmm	CTCF	wgEncodeAwgTfbsBroadHsmmCtcfUniPk
	EZH2	wgEncodeAwgTfbsBroadHsmmEzh239875UniPk
	CTCF	wgEncodeAwgTfbsBroadHsmmtCtcfUniPk
Huvec	CTCF	wgEncodeAwgTfbsBroadHuvecCtcfUniPk
	EZH2	wgEncodeAwgTfbsBroadHuvecEzh239875UniPk
	Pol2b	wgEncodeAwgTfbsBroadHuvecPol2bUniPk
	FOS	wgEncodeAwgTfbsSydhHuvecCfosUcdUniPk
	JUN	wgEncodeAwgTfbsSydhHuvecCjunUniPk
	GATA2	wgEncodeAwgTfbsSydhHuvecGata2UcdUniPk
	MAX	wgEncodeAwgTfbsSydhHuvecMaxUniPk
Nha	CTCF	wgEncodeAwgTfbsBroadNhaCtcfUniPk
Nhdfad	CTCF	wgEncodeAwgTfbsBroadNhdfadCtcfUniPk
Nhek	CTCF	wgEncodeAwgTfbsBroadNhekCtcfUniPk
	Pol2b	wgEncodeAwgTfbsBroadNhekPol2bUniPk
Nhlf	CTCF	wgEncodeAwgTfbsBroadNhlfCtcfUniPk
Osteobl	CTCF	wgEncodeAwgTfbsBroadOsteoblCtcfUniPk
Ecc1	FOXA1	wgEncodeAwgTfbsHaibEcc1Foxa1sc6553V0416102Dm002p1hUniPk
Gm12891	Pax5	wgEncodeAwgTfbsHaibGm12891Pax5c20V0416101UniPk
	NFKB	wgEncodeAwgTfbsSydhGm12891NfkbTnfaIggrabUniPk
Gm12892	Pax5	wgEncodeAwgTfbsHaibGm12892Pax5c20V0416101UniPk
	TAF1	wgEncodeAwgTfbsHaibGm12892Taf1V0416102UniPk
	YY1	wgEncodeAwgTfbsHaibGm12892Yy1V0416101UniPk
	NFKB	wgEncodeAwgTfbsSydhGm12892NfkbTnfaIggrabUniPk
Hct116	YY1	wgEncodeAwgTfbsHaibHct116Yy1sc281V0416101UniPk
Panc1	Sin3A	wgEncodeAwgTfbsHaibPanc1Sin3ak20V0416101UniPk
Sknshra	P300	wgEncodeAwgTfbsHaibSknshraP300V0416102UniPk
	YY1	wgEncodeAwgTfbsHaibSknshraYy1sc281V0416102UniPk
T47d	P300	wgEncodeAwgTfbsHaibT47dP300V0416102Dm002p1hUniPk
	FOXA1	wgEncodeAwgTfbsHaibT47dFoxa1sc6553V0416102Dm002p1hUniPk
	GATA3	wgEncodeAwgTfbsHaibT47dGata3sc268V0416102Dm002p1hUniPk
Gm10847	NFKB	wgEncodeAwgTfbsSydhGm10847NfkbTnfaIggrabUniPk
Nb4	MYC	wgEncodeAwgTfbsSydhNb4CmycUniPk
	MAX	wgEncodeAwgTfbsSydhNb4MaxUniPk
Sknsh	TAF1	wgEncodeAwgTfbsHaibSknshTaf1V0416101UniPk
Gm18526	NFKB	wgEncodeAwgTfbsSydhGm18526NfkbTnfaIggrabUniPk
Gm19099	NFKB	wgEncodeAwgTfbsSydhGm19099NfkbTnfaIggrabUniPk
Mcf10aes	FOS	wgEncodeAwgTfbsSydhMcf10aesCfosTam112hHvdUniPk
	FOS	wgEncodeAwgTfbsSydhMcf10aesCfosTam14hHvdUniPk
	FOS	wgEncodeAwgTfbsSydhMcf10aesCfosTamHvdUniPk
	STAT3	wgEncodeAwgTfbsSydhMcf10aesStat3Etoh01bUniPk
	STAT3	wgEncodeAwgTfbsSydhMcf10aesStat3Etoh01cUniPk
	STAT3	wgEncodeAwgTfbsSydhMcf10aesStat3Etoh01UniPk
	STAT3	wgEncodeAwgTfbsSydhMcf10aesStat3Tam112hHvdUniPk
	STAT3	wgEncodeAwgTfbsSydhMcf10aesStat3TamUniPk
Nt2d1	YY1	wgEncodeAwgTfbsSydhNt2d1Yy1UcdUniPk
Shsy5y	GATA2	wgEncodeAwgTfbsSydhShsy5yGata2UcdUniPk
	GATA3	wgEncodeAwgTfbsSydhShsy5yGata3sc269sc269UcdUniPk
Mcf7	GATA3	wgEncodeAwgTfbsSydhMcf7Gata3UcdUniPk
	HaE2F1	wgEncodeAwgTfbsSydhMcf7Hae2f1UcdUniPk
	CTCF	wgEncodeAwgTfbsUtaMcf7CtcfUniPk

Supplementary Table S6. The detailed prediction results of BERT-TFBS trained on the GM12878 cell line		
**GM12878 test set**		
TFs	Name	Accuracy
CTCF	wgEncodeAwgTfbsBroadGm12878CtcfUniPk	0.88014808
ELF1	wgEncodeAwgTfbsHaibGm12878Elf1sc631V0416101UniPk	0.84682861
GABP	wgEncodeAwgTfbsHaibGm12878GabpPcr2xUniPk	0.959247649
P300	wgEncodeAwgTfbsHaibGm12878P300Pcr1xUniPk	0.928928929
Pax5	wgEncodeAwgTfbsHaibGm12878Pax5c20Pcr1xUniPk	0.848070247
Pax5	wgEncodeAwgTfbsHaibGm12878Pax5n19Pcr1xUniPk	0.924794661
TAF1	wgEncodeAwgTfbsHaibGm12878Taf1Pcr1xUniPk	0.946537496
YY1	wgEncodeAwgTfbsHaibGm12878Yy1sc281Pcr1xUniPk	0.613752262
FOS	wgEncodeAwgTfbsSydhGm12878CfosUniPk	0.979118329
JUND	wgEncodeAwgTfbsSydhGm12878JundUniPk	0.980392157
MAX	wgEncodeAwgTfbsSydhGm12878MaxIggmusUniPk	0.898785425
NFKB	wgEncodeAwgTfbsSydhGm12878NfkbTnfaIggrabUniPk	0.943763087
P300	wgEncodeAwgTfbsSydhGm12878P300bUniPk	0.972904318
STAT3	wgEncodeAwgTfbsSydhGm12878Stat3IggmusUniPk	0.917467949
TBPI	wgEncodeAwgTfbsSydhGm12878TbpIggmusUniPk	0.936125654
MYC	wgEncodeAwgTfbsUtaGm12878CmycUniPk	0.937586685
CTCF	wgEncodeAwgTfbsUtaGm12878CtcfUniPk	0.925690021
	average	0.908243621
**Helas3 test set**		
TFs	Name	Accuracy
CTCF	wgEncodeAwgTfbsBroadHelas3CtcfUniPk	0.851421824
EZH2	wgEncodeAwgTfbsBroadHelas3Ezh239875UniPk	0.894878706
Pol2b	wgEncodeAwgTfbsBroadHelas3Pol2bUniPk	0.936877076
GABP	wgEncodeAwgTfbsHaibHelas3GabpPcr1xUniPk	0.96310241
CEBPB	wgEncodeAwgTfbsSydhHelas3CebpbIggrabUniPk	0.880888516
FOS	wgEncodeAwgTfbsSydhHelas3CfosUniPk	0.943584071
JUN	wgEncodeAwgTfbsSydhHelas3CjunIggrabUniPk	0.951753354
HaE2F1	wgEncodeAwgTfbsSydhHelas3Hae2f1UniPk	0.903548226
JUND	wgEncodeAwgTfbsSydhHelas3JundIggrabUniPk	0.896788991
MAX	wgEncodeAwgTfbsSydhHelas3MaxIggrabUniPk	0.854137691
P300	wgEncodeAwgTfbsSydhHelas3P300sc584sc584IggrabUniPk	0.937699681
STAT3	wgEncodeAwgTfbsSydhHelas3Stat3IggrabUniPk	0.931677019
CTCF	wgEncodeAwgTfbsUtaHelas3CtcfUniPk	0.925328396
	average	0.913206612
**Hepg2 test set**		
TFs	Name	Accuracy
CTCF	wgEncodeAwgTfbsBroadHepg2CtcfUniPk	0.920173199
EZH2	wgEncodeAwgTfbsBroadHepg2Ezh239875UniPk	0.934250765
CEBPB	wgEncodeAwgTfbsHaibHepg2Cebpbsc150V0416101UniPk	0.901234568
ELF1	wgEncodeAwgTfbsHaibHepg2Elf1sc631V0416101UniPk	0.875036075
FOS	wgEncodeAwgTfbsHaibHepg2Fosl2V0416101UniPk	0.905918367
FOXA1	wgEncodeAwgTfbsHaibHepg2Foxa1sc101058V0416101UniPk	0.889634865
FOXA1	wgEncodeAwgTfbsHaibHepg2Foxa1sc6553V0416101UniPk	0.897282174
GABP	wgEncodeAwgTfbsHaibHepg2GabpPcr2xUniPk	0.9224846
HDAC	wgEncodeAwgTfbsHaibHepg2Hdac2sc6296V0416101UniPk	0.860160428
JUND	wgEncodeAwgTfbsHaibHepg2JundPcr1xUniPk	0.917530631
P300	wgEncodeAwgTfbsHaibHepg2P300V0416101UniPk	0.91542563
Sin3A	wgEncodeAwgTfbsHaibHepg2Sin3ak20Pcr1xUniPk	0.903206107
SP1	wgEncodeAwgTfbsHaibHepg2Sp1Pcr1xUniPk	0.920653672
SP2	wgEncodeAwgTfbsHaibHepg2Sp2V0422111UniPk	0.862745098
SRF	wgEncodeAwgTfbsHaibHepg2SrfV0416101UniPk	0.876699029
TAF1	wgEncodeAwgTfbsHaibHepg2Taf1Pcr2xUniPk	0.926125555
TEAD4	wgEncodeAwgTfbsHaibHepg2Tead4sc101184V0422111UniPk	0.874285714
YY1	wgEncodeAwgTfbsHaibHepg2Yy1sc281V0416101UniPk	0.793529412
CEBPB	wgEncodeAwgTfbsSydhHepg2CebpbForsklnUniPk	0.867509621

CEBPB	wgEncodeAwgTfbsSydhHepg2CebpbIggrabUniPk	0.908275987
JUN	wgEncodeAwgTfbsSydhHepg2CjunIggrabUniPk	0.95479778
CoREST	wgEncodeAwgTfbsSydhHepg2Corestsc30189IggrabUniPk	0.803044719
JUND	wgEncodeAwgTfbsSydhHepg2JundIggrabUniPk	0.931191938
MAX	wgEncodeAwgTfbsSydhHepg2MaxIggrabUniPk	0.895408163
TBPI	wgEncodeAwgTfbsSydhHepg2TbpIggrabUniPk	0.916360968
MYC	wgEncodeAwgTfbsUtaHepg2CmycUniPk	0.83180778
	average	0.892491263
**K562 test set**		
TFs	Name	Accuracy
CTCF	wgEncodeAwgTfbsBroadK562CtcfUniPk	0.824151091
EZH2	wgEncodeAwgTfbsBroadK562Ezh239875UniPk	0.884615385
HDAC	wgEncodeAwgTfbsBroadK562Hdac2a300705aUniPk	0.881291548
HDAC	wgEncodeAwgTfbsBroadK562Hdac6a301341aUniPk	0.894736842
CEBPB	wgEncodeAwgTfbsHaibK562Cebpbsc150V0422111UniPk	0.884738772
ELF1	wgEncodeAwgTfbsHaibK562Elf1sc631V0416102UniPk	0.869662921
FOS	wgEncodeAwgTfbsHaibK562Fosl1sc183V0416101UniPk	0.920418372
GABP	wgEncodeAwgTfbsHaibK562GabpV0416101UniPk	0.905747947
GATA2	wgEncodeAwgTfbsHaibK562Gata2sc267Pcr1xUniPk	0.887395861
HDAC	wgEncodeAwgTfbsHaibK562Hdac2sc6296V0416102UniPk	0.843725943
MAX	wgEncodeAwgTfbsHaibK562MaxV0416102UniPk	0.81797928
Sin3A	wgEncodeAwgTfbsHaibK562Sin3ak20V0416101UniPk	0.896181384
SRF	wgEncodeAwgTfbsHaibK562SrfV0416101UniPk	0.892189219
TAF1	wgEncodeAwgTfbsHaibK562Taf1V0416101UniPk	0.933150497
TEAD4	wgEncodeAwgTfbsHaibK562Tead4sc101184V0422111UniPk	0.856906808
YY1	wgEncodeAwgTfbsHaibK562Yy1V0416101UniPk	0.802147873
YY1	wgEncodeAwgTfbsHaibK562Yy1V0416102UniPk	0.841680815
CEBPB	wgEncodeAwgTfbsSydhK562CebpbIggrabUniPk	0.891870244
FOS	wgEncodeAwgTfbsSydhK562CfosUniPk	0.963210702
JUN	wgEncodeAwgTfbsSydhK562CjunUniPk	0.923733333
CoREST	wgEncodeAwgTfbsSydhK562Corestsc30189IggrabUniPk	0.776448811
GATA2	wgEncodeAwgTfbsSydhK562Gata2UcdUniPk	0.883341335
JUND	wgEncodeAwgTfbsSydhK562JundIggrabUniPk	0.818773946
MAX	wgEncodeAwgTfbsSydhK562MaxIggrabUniPk	0.82188667
P300	wgEncodeAwgTfbsSydhK562P300IggrabUniPk	0.877733598
TBPI	wgEncodeAwgTfbsSydhK562TbpIggmusUniPk	0.908059354
YY1	wgEncodeAwgTfbsSydhK562Yy1UcdUniPk	0.956340956
FOS	wgEncodeAwgTfbsUchicagoK562EfosUniPk	0.934361014
GATA2	wgEncodeAwgTfbsUchicagoK562Egata2UniPk	0.909576837
HDAC	wgEncodeAwgTfbsUchicagoK562Ehdac8UniPk	0.855855856
JUND	wgEncodeAwgTfbsUchicagoK562EjundUniPk	0.890549195
CTCF	wgEncodeAwgTfbsUtaK562CtcfUniPk	0.930980608
	average	0.880607594

Supplementary Table S7. The detailed prediction results of BERT-TFBS trained on the Helas3 cell line		
**GM12878 test set**		
TFs	Name	Accuracy
CTCF	wgEncodeAwgTfbsBroadGm12878CtcfUniPk	0.921911152
ELF1	wgEncodeAwgTfbsHaibGm12878Elf1sc631V0416101UniPk	0.733018444
GABP	wgEncodeAwgTfbsHaibGm12878GabpPcr2xUniPk	0.88322884
P300	wgEncodeAwgTfbsHaibGm12878P300Pcr1xUniPk	0.817817818
Pax5	wgEncodeAwgTfbsHaibGm12878Pax5c20Pcr1xUniPk	0.727588319
Pax5	wgEncodeAwgTfbsHaibGm12878Pax5n19Pcr1xUniPk	0.790041068
TAF1	wgEncodeAwgTfbsHaibGm12878Taf1Pcr1xUniPk	0.817007535
YY1	wgEncodeAwgTfbsHaibGm12878Yy1sc281Pcr1xUniPk	0.519822339
FOS	wgEncodeAwgTfbsSydhGm12878CfosUniPk	0.969837587
JUND	wgEncodeAwgTfbsSydhGm12878JundUniPk	0.960784314
MAX	wgEncodeAwgTfbsSydhGm12878MaxIggmusUniPk	0.807287449
NFKB	wgEncodeAwgTfbsSydhGm12878NfkbTnfaIggrabUniPk	0.825904876
P300	wgEncodeAwgTfbsSydhGm12878P300bUniPk	0.880609653
STAT3	wgEncodeAwgTfbsSydhGm12878Stat3IggmusUniPk	0.832532051
TBPI	wgEncodeAwgTfbsSydhGm12878TbpIggmusUniPk	0.848516579
MYC	wgEncodeAwgTfbsUtaGm12878CmycUniPk	0.847434119
CTCF	wgEncodeAwgTfbsUtaGm12878CtcfUniPk	0.961146497
	average	0.832028744
**Helas3 test set**		
TFs	Name	Accuracy
CTCF	wgEncodeAwgTfbsBroadHelas3CtcfUniPk	0.899832727
EZH2	wgEncodeAwgTfbsBroadHelas3Ezh239875UniPk	0.757412399
Pol2b	wgEncodeAwgTfbsBroadHelas3Pol2bUniPk	0.825581395
GABP	wgEncodeAwgTfbsHaibHelas3GabpPcr1xUniPk	0.898343373
CEBPB	wgEncodeAwgTfbsSydhHelas3CebpbIggrabUniPk	0.907627829
FOS	wgEncodeAwgTfbsSydhHelas3CfosUniPk	0.968473451
JUN	wgEncodeAwgTfbsSydhHelas3CjunIggrabUniPk	0.973876206
HaE2F1	wgEncodeAwgTfbsSydhHelas3Hae2f1UniPk	0.865067466
JUND	wgEncodeAwgTfbsSydhHelas3JundIggrabUniPk	0.919724771
MAX	wgEncodeAwgTfbsSydhHelas3MaxIggrabUniPk	0.801286509
P300	wgEncodeAwgTfbsSydhHelas3P300sc584sc584IggrabUniPk	0.958266773
STAT3	wgEncodeAwgTfbsSydhHelas3Stat3IggrabUniPk	0.939715016
CTCF	wgEncodeAwgTfbsUtaHelas3CtcfUniPk	0.954333069
	average	0.897656999
**Hepg2 test set**		
TFs	Name	Accuracy
CTCF	wgEncodeAwgTfbsBroadHepg2CtcfUniPk	0.953258577
EZH2	wgEncodeAwgTfbsBroadHepg2Ezh239875UniPk	0.785932722
CEBPB	wgEncodeAwgTfbsHaibHepg2Cebpbsc150V0416101UniPk	0.932098765
ELF1	wgEncodeAwgTfbsHaibHepg2Elf1sc631V0416101UniPk	0.794805195
FOS	wgEncodeAwgTfbsHaibHepg2Fosl2V0416101UniPk	0.886734694
FOXA1	wgEncodeAwgTfbsHaibHepg2Foxa1sc101058V0416101UniPk	0.833333333
FOXA1	wgEncodeAwgTfbsHaibHepg2Foxa1sc6553V0416101UniPk	0.848920863
GABP	wgEncodeAwgTfbsHaibHepg2GabpPcr2xUniPk	0.843429158
HDAC	wgEncodeAwgTfbsHaibHepg2Hdac2sc6296V0416101UniPk	0.78368984
JUND	wgEncodeAwgTfbsHaibHepg2JundPcr1xUniPk	0.888548539
P300	wgEncodeAwgTfbsHaibHepg2P300V0416101UniPk	0.855855856
Sin3A	wgEncodeAwgTfbsHaibHepg2Sin3ak20Pcr1xUniPk	0.809160305
SP1	wgEncodeAwgTfbsHaibHepg2Sp1Pcr1xUniPk	0.847410908
SP2	wgEncodeAwgTfbsHaibHepg2Sp2V0422111UniPk	0.719607843
SRF	wgEncodeAwgTfbsHaibHepg2SrfV0416101UniPk	0.823300971
TAF1	wgEncodeAwgTfbsHaibHepg2Taf1Pcr2xUniPk	0.809131262
TEAD4	wgEncodeAwgTfbsHaibHepg2Tead4sc101184V0422111UniPk	0.842016807
YY1	wgEncodeAwgTfbsHaibHepg2Yy1sc281V0416101UniPk	0.692058824
CEBPB	wgEncodeAwgTfbsSydhHepg2CebpbForsklnUniPk	0.889499725

CEBPB	wgEncodeAwgTfbsSydhHepg2CebpbIggrabUniPk	0.956175299
JUN	wgEncodeAwgTfbsSydhHepg2CjunIggrabUniPk	0.974623315
CoREST	wgEncodeAwgTfbsSydhHepg2Corestsc30189IggrabUniPk	0.714557564
JUND	wgEncodeAwgTfbsSydhHepg2JundIggrabUniPk	0.958431743
MAX	wgEncodeAwgTfbsSydhHepg2MaxIggrabUniPk	0.825680272
TBPI	wgEncodeAwgTfbsSydhHepg2TbpIggrabUniPk	0.829420396
MYC	wgEncodeAwgTfbsUtaHepg2CmycUniPk	0.76201373
	average	0.840757558
**K562 test set**		
TFs	Name	Accuracy
CTCF	wgEncodeAwgTfbsBroadK562CtcfUniPk	0.881103826
EZH2	wgEncodeAwgTfbsBroadK562Ezh239875UniPk	0.733974359
HDAC	wgEncodeAwgTfbsBroadK562Hdac2a300705aUniPk	0.717948718
HDAC	wgEncodeAwgTfbsBroadK562Hdac6a301341aUniPk	0.736842105
CEBPB	wgEncodeAwgTfbsHaibK562Cebpbsc150V0422111UniPk	0.90467461
ELF1	wgEncodeAwgTfbsHaibK562Elf1sc631V0416102UniPk	0.764606742
FOS	wgEncodeAwgTfbsHaibK562Fosl1sc183V0416101UniPk	0.937244202
GABP	wgEncodeAwgTfbsHaibK562GabpV0416101UniPk	0.815423063
GATA2	wgEncodeAwgTfbsHaibK562Gata2sc267Pcr1xUniPk	0.826928245
HDAC	wgEncodeAwgTfbsHaibK562Hdac2sc6296V0416102UniPk	0.743648961
MAX	wgEncodeAwgTfbsHaibK562MaxV0416102UniPk	0.791578913
Sin3A	wgEncodeAwgTfbsHaibK562Sin3ak20V0416101UniPk	0.799920446
SRF	wgEncodeAwgTfbsHaibK562SrfV0416101UniPk	0.839383938
TAF1	wgEncodeAwgTfbsHaibK562Taf1V0416101UniPk	0.846074734
TEAD4	wgEncodeAwgTfbsHaibK562Tead4sc101184V0422111UniPk	0.787012558
YY1	wgEncodeAwgTfbsHaibK562Yy1V0416101UniPk	0.705080545
YY1	wgEncodeAwgTfbsHaibK562Yy1V0416102UniPk	0.709040747
CEBPB	wgEncodeAwgTfbsSydhK562CebpbIggrabUniPk	0.931117341
FOS	wgEncodeAwgTfbsSydhK562CfosUniPk	0.955183946
JUN	wgEncodeAwgTfbsSydhK562CjunUniPk	0.9504
CoREST	wgEncodeAwgTfbsSydhK562Corestsc30189IggrabUniPk	0.704115051
GATA2	wgEncodeAwgTfbsSydhK562Gata2UcdUniPk	0.782045127
JUND	wgEncodeAwgTfbsSydhK562JundIggrabUniPk	0.799489144
MAX	wgEncodeAwgTfbsSydhK562MaxIggrabUniPk	0.74557558
P300	wgEncodeAwgTfbsSydhK562P300IggrabUniPk	0.819681909
TBPI	wgEncodeAwgTfbsSydhK562TbpIggmusUniPk	0.818737271
YY1	wgEncodeAwgTfbsSydhK562Yy1UcdUniPk	0.826403326
FOS	wgEncodeAwgTfbsUchicagoK562EfosUniPk	0.956240676
GATA2	wgEncodeAwgTfbsUchicagoK562Egata2UniPk	0.830289532
HDAC	wgEncodeAwgTfbsUchicagoK562Ehdac8UniPk	0.726726727
JUND	wgEncodeAwgTfbsUchicagoK562EjundUniPk	0.874053949
CTCF	wgEncodeAwgTfbsUtaK562CtcfUniPk	0.962135833
	average	0.819458816

Supplementary Table S8. The detailed prediction results of BERT-TFBS trained on the Hepg2 cell line.		
**GM12878 test set**		
TFs	Name	Accuracy
CTCF	wgEncodeAwgTfbsBroadGm12878CtcfUniPk	0.899352152
ELF1	wgEncodeAwgTfbsHaibGm12878Elf1sc631V0416101UniPk	0.706927575
GABP	wgEncodeAwgTfbsHaibGm12878GabpPcr2xUniPk	0.840909091
P300	wgEncodeAwgTfbsHaibGm12878P300Pcr1xUniPk	0.826826827
Pax5	wgEncodeAwgTfbsHaibGm12878Pax5c20Pcr1xUniPk	0.744333265
Pax5	wgEncodeAwgTfbsHaibGm12878Pax5n19Pcr1xUniPk	0.815451745
TAF1	wgEncodeAwgTfbsHaibGm12878Taf1Pcr1xUniPk	0.81844277
YY1	wgEncodeAwgTfbsHaibGm12878Yy1sc281Pcr1xUniPk	0.518999835
FOS	wgEncodeAwgTfbsSydhGm12878CfosUniPk	0.932714617
JUND	wgEncodeAwgTfbsSydhGm12878JundUniPk	0.952069717
MAX	wgEncodeAwgTfbsSydhGm12878MaxIggmusUniPk	0.801214575
NFKB	wgEncodeAwgTfbsSydhGm12878NfkbTnfaIggrabUniPk	0.836374514
P300	wgEncodeAwgTfbsSydhGm12878P300bUniPk	0.883149873
STAT3	wgEncodeAwgTfbsSydhGm12878Stat3IggmusUniPk	0.83974359
TBPI	wgEncodeAwgTfbsSydhGm12878TbpIggmusUniPk	0.839790576
MYC	wgEncodeAwgTfbsUtaGm12878CmycUniPk	0.822468793
CTCF	wgEncodeAwgTfbsUtaGm12878CtcfUniPk	0.940127389
	average	0.824640994
**Helas3 test set**		
TFs	Name	Accuracy
CTCF	wgEncodeAwgTfbsBroadHelas3CtcfUniPk	0.877791991
EZH2	wgEncodeAwgTfbsBroadHelas3Ezh239875UniPk	0.784366577
Pol2b	wgEncodeAwgTfbsBroadHelas3Pol2bUniPk	0.827242525
GABP	wgEncodeAwgTfbsHaibHelas3GabpPcr1xUniPk	0.865963855
CEBPB	wgEncodeAwgTfbsSydhHelas3CebpbIggrabUniPk	0.876865046
FOS	wgEncodeAwgTfbsSydhHelas3CfosUniPk	0.92699115
JUN	wgEncodeAwgTfbsSydhHelas3CjunIggrabUniPk	0.936926336
HaE2F1	wgEncodeAwgTfbsSydhHelas3Hae2f1UniPk	0.856071964
JUND	wgEncodeAwgTfbsSydhHelas3JundIggrabUniPk	0.887123198
MAX	wgEncodeAwgTfbsSydhHelas3MaxIggrabUniPk	0.761300417
P300	wgEncodeAwgTfbsSydhHelas3P300sc584sc584IggrabUniPk	0.92571885
STAT3	wgEncodeAwgTfbsSydhHelas3Stat3IggrabUniPk	0.91012057
CTCF	wgEncodeAwgTfbsUtaHelas3CtcfUniPk	0.936348409
	average	0.874833145
**Hepg2 test set**		
TFs	Name	Accuracy
CTCF	wgEncodeAwgTfbsBroadHepg2CtcfUniPk	0.932607972
EZH2	wgEncodeAwgTfbsBroadHepg2Ezh239875UniPk	0.796636086
CEBPB	wgEncodeAwgTfbsHaibHepg2Cebpbsc150V0416101UniPk	0.908529742
ELF1	wgEncodeAwgTfbsHaibHepg2Elf1sc631V0416101UniPk	0.778066378
FOS	wgEncodeAwgTfbsHaibHepg2Fosl2V0416101UniPk	0.888979592
FOXA1	wgEncodeAwgTfbsHaibHepg2Foxa1sc101058V0416101UniPk	0.888928151
FOXA1	wgEncodeAwgTfbsHaibHepg2Foxa1sc6553V0416101UniPk	0.900979217
GABP	wgEncodeAwgTfbsHaibHepg2GabpPcr2xUniPk	0.820328542
HDAC	wgEncodeAwgTfbsHaibHepg2Hdac2sc6296V0416101UniPk	0.832352941
JUND	wgEncodeAwgTfbsHaibHepg2JundPcr1xUniPk	0.893967955
P300	wgEncodeAwgTfbsHaibHepg2P300V0416101UniPk	0.892627321
Sin3A	wgEncodeAwgTfbsHaibHepg2Sin3ak20Pcr1xUniPk	0.804885496
SP1	wgEncodeAwgTfbsHaibHepg2Sp1Pcr1xUniPk	0.889348297
SP2	wgEncodeAwgTfbsHaibHepg2Sp2V0422111UniPk	0.766666667
SRF	wgEncodeAwgTfbsHaibHepg2SrfV0416101UniPk	0.829126214
TAF1	wgEncodeAwgTfbsHaibHepg2Taf1Pcr2xUniPk	0.824350032
TEAD4	wgEncodeAwgTfbsHaibHepg2Tead4sc101184V0422111UniPk	0.854117647
YY1	wgEncodeAwgTfbsHaibHepg2Yy1sc281V0416101UniPk	0.687941176
CEBPB	wgEncodeAwgTfbsSydhHepg2CebpbForsklnUniPk	0.861462342

CEBPB	wgEncodeAwgTfbsSydhHepg2CebpbIggrabUniPk	0.926747555
JUN	wgEncodeAwgTfbsSydhHepg2CjunIggrabUniPk	0.963124504
CoREST	wgEncodeAwgTfbsSydhHepg2Corestsc30189IggrabUniPk	0.73549001
JUND	wgEncodeAwgTfbsSydhHepg2JundIggrabUniPk	0.941584003
MAX	wgEncodeAwgTfbsSydhHepg2MaxIggrabUniPk	0.81505102
TBPI	wgEncodeAwgTfbsSydhHepg2TbpIggrabUniPk	0.828319883
MYC	wgEncodeAwgTfbsUtaHepg2CmycUniPk	0.747139588
	average	0.846513782
**K562 test set**		
TFs	Name	Accuracy
CTCF	wgEncodeAwgTfbsBroadK562CtcfUniPk	0.858303161
EZH2	wgEncodeAwgTfbsBroadK562Ezh239875UniPk	0.794871795
HDAC	wgEncodeAwgTfbsBroadK562Hdac2a300705aUniPk	0.75688509
HDAC	wgEncodeAwgTfbsBroadK562Hdac6a301341aUniPk	0.754385965
CEBPB	wgEncodeAwgTfbsHaibK562Cebpbsc150V0422111UniPk	0.871906508
ELF1	wgEncodeAwgTfbsHaibK562Elf1sc631V0416102UniPk	0.73164794
FOS	wgEncodeAwgTfbsHaibK562Fosl1sc183V0416101UniPk	0.894952251
GABP	wgEncodeAwgTfbsHaibK562GabpV0416101UniPk	0.79721528
GATA2	wgEncodeAwgTfbsHaibK562Gata2sc267Pcr1xUniPk	0.821015856
HDAC	wgEncodeAwgTfbsHaibK562Hdac2sc6296V0416102UniPk	0.75134719
MAX	wgEncodeAwgTfbsHaibK562MaxV0416102UniPk	0.713267238
Sin3A	wgEncodeAwgTfbsHaibK562Sin3ak20V0416101UniPk	0.801511535
SRF	wgEncodeAwgTfbsHaibK562SrfV0416101UniPk	0.815181518
TAF1	wgEncodeAwgTfbsHaibK562Taf1V0416101UniPk	0.832704834
TEAD4	wgEncodeAwgTfbsHaibK562Tead4sc101184V0422111UniPk	0.781229346
YY1	wgEncodeAwgTfbsHaibK562Yy1V0416101UniPk	0.686080132
YY1	wgEncodeAwgTfbsHaibK562Yy1V0416102UniPk	0.701400679
CEBPB	wgEncodeAwgTfbsSydhK562CebpbIggrabUniPk	0.896809505
FOS	wgEncodeAwgTfbsSydhK562CfosUniPk	0.931772575
JUN	wgEncodeAwgTfbsSydhK562CjunUniPk	0.910933333
CoREST	wgEncodeAwgTfbsSydhK562Corestsc30189IggrabUniPk	0.707247615
GATA2	wgEncodeAwgTfbsSydhK562Gata2UcdUniPk	0.788286126
JUND	wgEncodeAwgTfbsSydhK562JundIggrabUniPk	0.775095785
MAX	wgEncodeAwgTfbsSydhK562MaxIggrabUniPk	0.733235915
P300	wgEncodeAwgTfbsSydhK562P300IggrabUniPk	0.808946322
TBPI	wgEncodeAwgTfbsSydhK562TbpIggmusUniPk	0.81233634
YY1	wgEncodeAwgTfbsSydhK562Yy1UcdUniPk	0.803534304
FOS	wgEncodeAwgTfbsUchicagoK562EfosUniPk	0.913973148
GATA2	wgEncodeAwgTfbsUchicagoK562Egata2UniPk	0.832962138
HDAC	wgEncodeAwgTfbsUchicagoK562Ehdac8UniPk	0.762762763
JUND	wgEncodeAwgTfbsUchicagoK562EjundUniPk	0.854065593
CTCF	wgEncodeAwgTfbsUtaK562CtcfUniPk	0.94265233
	average	0.807453753

Supplementary Table S9. The detailed prediction results of BERT-TFBS trained on the K562 cell line.		
**GM12878 test set**		
TFs	Name	Accuracy
CTCF	wgEncodeAwgTfbsBroadGm12878CtcfUniPk	0.907565942
ELF1	wgEncodeAwgTfbsHaibGm12878Elf1sc631V0416101UniPk	0.793747188
GABP	wgEncodeAwgTfbsHaibGm12878GabpPcr2xUniPk	0.928683386
P300	wgEncodeAwgTfbsHaibGm12878P300Pcr1xUniPk	0.864864865
Pax5	wgEncodeAwgTfbsHaibGm12878Pax5c20Pcr1xUniPk	0.778435777
Pax5	wgEncodeAwgTfbsHaibGm12878Pax5n19Pcr1xUniPk	0.854466119
TAF1	wgEncodeAwgTfbsHaibGm12878Taf1Pcr1xUniPk	0.875852171
YY1	wgEncodeAwgTfbsHaibGm12878Yy1sc281Pcr1xUniPk	0.584964632
FOS	wgEncodeAwgTfbsSydhGm12878CfosUniPk	0.979118329
JUND	wgEncodeAwgTfbsSydhGm12878JundUniPk	0.969498911
MAX	wgEncodeAwgTfbsSydhGm12878MaxIggmusUniPk	0.845748988
NFKB	wgEncodeAwgTfbsSydhGm12878NfkbTnfaIggrabUniPk	0.868082561
P300	wgEncodeAwgTfbsSydhGm12878P300bUniPk	0.924640135
STAT3	wgEncodeAwgTfbsSydhGm12878Stat3IggmusUniPk	0.862179487
TBPI	wgEncodeAwgTfbsSydhGm12878TbpIggmusUniPk	0.892146597
MYC	wgEncodeAwgTfbsUtaGm12878CmycUniPk	0.879334258
CTCF	wgEncodeAwgTfbsUtaGm12878CtcfUniPk	0.95626327
	average	0.868564272
**Helas3 test set**		
TFs	Name	Accuracy
CTCF	wgEncodeAwgTfbsBroadHelas3CtcfUniPk	0.884974909
EZH2	wgEncodeAwgTfbsBroadHelas3Ezh239875UniPk	0.832884097
Pol2b	wgEncodeAwgTfbsBroadHelas3Pol2bUniPk	0.863787375
GABP	wgEncodeAwgTfbsHaibHelas3GabpPcr1xUniPk	0.938253012
CEBPB	wgEncodeAwgTfbsSydhHelas3CebpbIggrabUniPk	0.900335289
FOS	wgEncodeAwgTfbsSydhHelas3CfosUniPk	0.971238938
JUN	wgEncodeAwgTfbsSydhHelas3CjunIggrabUniPk	0.966815721
HaE2F1	wgEncodeAwgTfbsSydhHelas3Hae2f1UniPk	0.867066467
JUND	wgEncodeAwgTfbsSydhHelas3JundIggrabUniPk	0.916939712
MAX	wgEncodeAwgTfbsSydhHelas3MaxIggrabUniPk	0.824756606
P300	wgEncodeAwgTfbsSydhHelas3P300sc584sc584IggrabUniPk	0.952076677
STAT3	wgEncodeAwgTfbsSydhHelas3Stat3IggrabUniPk	0.942272561
CTCF	wgEncodeAwgTfbsUtaHelas3CtcfUniPk	0.952040906
	average	0.908726329
**Hepg2 test set**		
TFs	Name	Accuracy
CTCF	wgEncodeAwgTfbsBroadHepg2CtcfUniPk	0.943155324
EZH2	wgEncodeAwgTfbsBroadHepg2Ezh239875UniPk	0.822629969
CEBPB	wgEncodeAwgTfbsHaibHepg2Cebpbsc150V0416101UniPk	0.917789001
ELF1	wgEncodeAwgTfbsHaibHepg2Elf1sc631V0416101UniPk	0.851659452
FOS	wgEncodeAwgTfbsHaibHepg2Fosl2V0416101UniPk	0.899183673
FOXA1	wgEncodeAwgTfbsHaibHepg2Foxa1sc101058V0416101UniPk	0.866077739
FOXA1	wgEncodeAwgTfbsHaibHepg2Foxa1sc6553V0416101UniPk	0.878197442
GABP	wgEncodeAwgTfbsHaibHepg2GabpPcr2xUniPk	0.881930185
HDAC	wgEncodeAwgTfbsHaibHepg2Hdac2sc6296V0416101UniPk	0.823262032
JUND	wgEncodeAwgTfbsHaibHepg2JundPcr1xUniPk	0.908576814
P300	wgEncodeAwgTfbsHaibHepg2P300V0416101UniPk	0.885273028
Sin3A	wgEncodeAwgTfbsHaibHepg2Sin3ak20Pcr1xUniPk	0.841526718
SP1	wgEncodeAwgTfbsHaibHepg2Sp1Pcr1xUniPk	0.879110061
SP2	wgEncodeAwgTfbsHaibHepg2Sp2V0422111UniPk	0.835294118
SRF	wgEncodeAwgTfbsHaibHepg2SrfV0416101UniPk	0.866019417
TAF1	wgEncodeAwgTfbsHaibHepg2Taf1Pcr2xUniPk	0.844641725
TEAD4	wgEncodeAwgTfbsHaibHepg2Tead4sc101184V0422111UniPk	0.865882353
YY1	wgEncodeAwgTfbsHaibHepg2Yy1sc281V0416101UniPk	0.747647059
CEBPB	wgEncodeAwgTfbsSydhHepg2CebpbForsklnUniPk	0.879054426

CEBPB	wgEncodeAwgTfbsSydhHepg2CebpbIggrabUniPk	0.94530967
JUN	wgEncodeAwgTfbsSydhHepg2CjunIggrabUniPk	0.970261697
CoREST	wgEncodeAwgTfbsSydhHepg2Corestsc30189IggrabUniPk	0.762131304
JUND	wgEncodeAwgTfbsSydhHepg2JundIggrabUniPk	0.955754999
MAX	wgEncodeAwgTfbsSydhHepg2MaxIggrabUniPk	0.867346939
TBPI	wgEncodeAwgTfbsSydhHepg2TbpIggrabUniPk	0.859134263
MYC	wgEncodeAwgTfbsUtaHepg2CmycUniPk	0.798627002
	average	0.869056785
	**K562 test set**	
TFs	Name	Accuracy
CTCF	wgEncodeAwgTfbsBroadK562CtcfUniPk	0.858596732
EZH2	wgEncodeAwgTfbsBroadK562Ezh239875UniPk	0.820512821
HDAC	wgEncodeAwgTfbsBroadK562Hdac2a300705aUniPk	0.790123457
HDAC	wgEncodeAwgTfbsBroadK562Hdac6a301341aUniPk	0.789473684
CEBPB	wgEncodeAwgTfbsHaibK562Cebpbsc150V0422111UniPk	0.91796517
ELF1	wgEncodeAwgTfbsHaibK562Elf1sc631V0416102UniPk	0.842883895
FOS	wgEncodeAwgTfbsHaibK562Fosl1sc183V0416101UniPk	0.950432015
GABP	wgEncodeAwgTfbsHaibK562GabpV0416101UniPk	0.897893609
GATA2	wgEncodeAwgTfbsHaibK562Gata2sc267Pcr1xUniPk	0.946519753
HDAC	wgEncodeAwgTfbsHaibK562Hdac2sc6296V0416102UniPk	0.838337182
MAX	wgEncodeAwgTfbsHaibK562MaxV0416102UniPk	0.864151753
Sin3A	wgEncodeAwgTfbsHaibK562Sin3ak20V0416101UniPk	0.851233095
SRF	wgEncodeAwgTfbsHaibK562SrfV0416101UniPk	0.887788779
TAF1	wgEncodeAwgTfbsHaibK562Taf1V0416101UniPk	0.897154611
TEAD4	wgEncodeAwgTfbsHaibK562Tead4sc101184V0422111UniPk	0.881031064
YY1	wgEncodeAwgTfbsHaibK562Yy1V0416101UniPk	0.773647253
YY1	wgEncodeAwgTfbsHaibK562Yy1V0416102UniPk	0.818760611
CEBPB	wgEncodeAwgTfbsSydhK562CebpbIggrabUniPk	0.92844747
FOS	wgEncodeAwgTfbsSydhK562CfosUniPk	0.981270903
JUN	wgEncodeAwgTfbsSydhK562CjunUniPk	0.962133333
CoREST	wgEncodeAwgTfbsSydhK562Corestsc30189IggrabUniPk	0.789121458
GATA2	wgEncodeAwgTfbsSydhK562Gata2UcdUniPk	0.926068171
JUND	wgEncodeAwgTfbsSydhK562JundIggrabUniPk	0.836781609
MAX	wgEncodeAwgTfbsSydhK562MaxIggrabUniPk	0.802565352
P300	wgEncodeAwgTfbsSydhK562P300IggrabUniPk	0.901590457
TBPI	wgEncodeAwgTfbsSydhK562TbpIggmusUniPk	0.87634565
YY1	wgEncodeAwgTfbsSydhK562Yy1UcdUniPk	0.941787942
FOS	wgEncodeAwgTfbsUchicagoK562EfosUniPk	0.961213327
GATA2	wgEncodeAwgTfbsUchicagoK562Egata2UniPk	0.927839644
HDAC	wgEncodeAwgTfbsUchicagoK562Ehdac8UniPk	0.762762763
JUND	wgEncodeAwgTfbsUchicagoK562EjundUniPk	0.907432564
CTCF	wgEncodeAwgTfbsUtaK562CtcfUniPk	0.950096498
	average	0.877561332

Supplementary Table S10. Prediction performances of BERT-TFBS and other models on the 165 ChIP-seq datasets.

TFs	Datasets	BERT-TFBS			DeepBind			DanQ			DLBSS			CRPTS			D-SSCA			DSAC		
		Accuracy	ROC AUC	PR AUC	Accuracy	ROC AUC	PR AUC	Accuracy	ROC AUC	PR AUC	Accuracy	ROC AUC	PR AUC	Accuracy	ROC AUC	PR AUC	Accuracy	ROC AUC	PR AUC	Accuracy	ROC AUC	PR AUC
CEBPB	wgEncodeAwgTfbsHaibHepg2Cebpbsc150V0416101UniPk	0.925733539	0.977057894	0.977443323	0.903411484	0.963637	0.965857	0.90285	0.959139	0.962157	0.916047	0.968525	0.970415	0.907623	0.963799	0.965592	0.90299	0.963861	0.965362	0.909085	0.965578	0.965551
	wgEncodeAwgTfbsHaibK562Cebpbsc150V0422111UniPk	0.895936457	0.957432088	0.959448446	0.865431104	0.932552	0.937684	0.857488	0.933315	0.94112	0.882583	0.946604	0.952168	0.86428	0.933759	0.93738	0.86451	0.932003	0.936249			
	wgEncodeAwgTfbsSydhA549CebpbIggrabUniPk	0.94324093	0.984178036	0.982469401	0.915914927	0.970718	0.971065	0.925133	0.974723	0.975977	0.931059	0.979998	0.980597	0.925463	0.975266	0.974613	0.925067	0.975517	0.974745			
	wgEncodeAwgTfbsSydhH1hescCebpbIggrabUniPk	0.946878065	0.986226118	0.98529346	0.921379536	0.97032	0.971211	0.924158	0.968767	0.971392	0.935927	0.978635	0.978587	0.926447	0.972429	0.973819	0.926773	0.973007	0.973029			
	wgEncodeAwgTfbsSydhHelas3CebpbIggrabUniPk	0.906423875	0.962815576	0.960849273	0.857696847	0.930057	0.933546	0.862038	0.933612	0.939015	0.86482	0.946389	0.949183	0.860142	0.936603	0.938482	0.868488	0.939356	0.940686			
	wgEncodeAwgTfbsSydhHepg2CebpbForsklnUniPk	0.916184174	0.971520238	0.973219955	0.868899917	0.937184	0.94121	0.882685	0.94564	0.951396	0.891922	0.959188	0.962445	0.884753	0.946234	0.950003	0.872071	0.948396	0.950437			
	wgEncodeAwgTfbsSydhHepg2CebpbIggrabUniPk	0.951703398	0.985223841	0.978532554	0.926792046	0.975878	0.972939	0.931954	0.98131	0.979728	0.942637	0.983957	0.982849	0.931819	0.979011	0.975761	0.930248	0.97696	0.973792			
	wgEncodeAwgTfbsSydhImr90CebpbIggrabUniPk	0.931055855	0.976487763	0.971653831	0.900977104	0.961092	0.95959	0.904732	0.964932	0.965711	0.910748	0.97183	0.971594	0.901779	0.961879	0.960427	0.904222	0.963924	0.962063			
	wgEncodeAwgTfbsSydhK562CebpbIggrabUniPk	0.941472458	0.981731929	0.979551751	0.908567267	0.965457	0.964928	0.911149	0.969314	0.971564	0.918101	0.973813	0.974934	0.915188	0.969313	0.970086	0.916314	0.966381	0.966325			
CoREST	wgEncodeAwgTfbsSydhHepg2Corestsc30189IggrabUniPk	0.778769375	0.857508266	0.866414595	0.635979333	0.689056	0.675565	0.61954	0.678559	0.65936	0.66698	0.72603	0.717678	0.639737	0.705367	0.70339	0.674495	0.753529	0.746852	0.755452	0.835737	0.839527
	wgEncodeAwgTfbsSydhK562Corestsc30189IggrabUniPk	0.865437985	0.937758724	0.938856027	0.750729382	0.83206	0.841542	0.771793	0.856105	0.864789	0.772362	0.858981	0.864796	0.773714	0.856097	0.861704	0.782039	0.866777	0.868814			
CTCF	wgEncodeAwgTfbsBroadDnd41CtcfUniPk	0.925604912	0.975697602	0.977076661	0.908528092	0.965868	0.970118	0.914822	0.967764	0.973603	0.917608	0.973155	0.976688	0.897126	0.960522	0.964531	0.900738	0.963326	0.967042	0.908346	0.966755	0.969759
	wgEncodeAwgTfbsBroadGm12878CtcfUniPk	0.917333948	0.970700758	0.972521345	0.903565874	0.958244	0.966214	0.887148	0.946912	0.957533	0.900743	0.961081	0.968647	0.895386	0.954172	0.961176	0.895847	0.954828	0.962171			
	wgEncodeAwgTfbsBroadH1hescCtcfUniPk	0.920369078	0.970094814	0.963105715	0.902768086	0.961798	0.965056	0.907692	0.965553	0.96974	0.903427	0.972511	0.974258	0.894976	0.958529	0.961062	0.900675	0.96189	0.965032			
	wgEncodeAwgTfbsBroadHelas3CtcfUniPk	0.9045551	0.95829178	0.959411659	0.877708138	0.942763	0.954147	0.880817	0.94132	0.95351	0.892464	0.951607	0.960867	0.863643	0.935706	0.945741	0.87302	0.942021	0.952384			
	wgEncodeAwgTfbsBroadHepg2CtcfUniPk	0.930206836	0.976886474	0.975849721	0.921579471	0.972385	0.976034	0.917155	0.965121	0.97166	0.921248	0.97241	0.976584	0.906979	0.964343	0.968751	0.910242	0.966504	0.97055			
	wgEncodeAwgTfbsBroadHmecCtcfUniPk	0.939162514	0.98090641	0.981947193	0.925053395	0.97412	0.977957	0.926607	0.972041	0.978196	0.929001	0.977667	0.98129	0.911721	0.967678	0.972065	0.91541	0.970644	0.975013			
	wgEncodeAwgTfbsBroadHsmmCtcfUniPk	0.924392713	0.971672959	0.968000427	0.905212551	0.965507	0.969444	0.920294	0.970854	0.975238	0.922672	0.974848	0.977903	0.895192	0.958662	0.962853	0.898178	0.963555	0.966895			
	wgEncodeAwgTfbsBroadHsmmtCtcfUniPk	0.919255994	0.970613446	0.970014101	0.905924094	0.962145	0.967782	0.91001	0.960943	0.967923	0.907483	0.965505	0.970832	0.89614	0.954873	0.9608	0.899796	0.961232	0.966086			
	wgEncodeAwgTfbsBroadHuvecCtcfUniPk	0.929849924	0.97622877	0.978231758	0.912777089	0.965665	0.971053	0.922002	0.968994	0.975087	0.925306	0.975336	0.979839	0.913259	0.966052	0.971145	0.912708	0.965096	0.969878			
	wgEncodeAwgTfbsBroadK562CtcfUniPk	0.89425516	0.954492403	0.957885105	0.877940811	0.938282	0.951482	0.868192	0.931618	0.946107	0.877742	0.941702	0.953559	0.866302	0.932941	0.945922	0.866352	0.934551	0.946901			
	wgEncodeAwgTfbsBroadNhaCtcfUniPk	0.933351214	0.976399959	0.977250157	0.917661258	0.969005	0.974367	0.919874	0.967265	0.973886	0.928389	0.975596	0.979682	0.910152	0.966125	0.97074	0.915113	0.966897	0.97236			
	wgEncodeAwgTfbsBroadNhdfadCtcfUniPk	0.930021621	0.977562455	0.977345998	0.910404472	0.967442	0.971529	0.920371	0.968838	0.973321	0.924274	0.975609	0.979546	0.890735	0.956153	0.960934	0.90761	0.965445	0.96918			
	wgEncodeAwgTfbsBroadNhekCtcfUniPk	0.913865892	0.968615962	0.96847131	0.89204452	0.952794	0.959988	0.90449	0.95806	0.965944	0.910631	0.96391	0.970135	0.890838	0.953397	0.959886	0.891167	0.954981	0.960731			
	wgEncodeAwgTfbsBroadNhlfCtcfUniPk	0.918617542	0.970682508	0.970479912	0.911118357	0.96499	0.971019	0.902837	0.957896	0.966919	0.91001	0.969146	0.974358	0.89925	0.958888	0.964857	0.901402	0.960697	0.967132			
	wgEncodeAwgTfbsBroadOsteoblCtcfUniPk	0.906723001	0.961251479	0.962020598	0.887408302	0.947745	0.958981	0.891123	0.947277	0.958916	0.89103	0.951915	0.962246	0.872087	0.93857	0.950136	0.883462	0.946121	0.955801			
	wgEncodeAwgTfbsUtaGm12878CtcfUniPk	0.933382758	0.978299721	0.976123674	0.925916119	0.976644	0.978643	0.931159	0.979427	0.981746	0.931794	0.981454	0.983111	0.91649	0.971758	0.97362	0.923215	0.976106	0.97744			
	wgEncodeAwgTfbsUtaH1hescCtcfUniPk	0.919413052	0.973036049	0.972457616	0.900952606	0.964168	0.966341	0.911189	0.968227	0.971054	0.909177	0.970228	0.972064	0.888705	0.954728	0.957453	0.883794	0.956666	0.958917			
	wgEncodeAwgTfbsUtaHelas3CtcfUniPk	0.937766824	0.979761229	0.974368905	0.915408653	0.972549	0.973686	0.939527	0.983539	0.984326	0.933762	0.982495	0.983156	0.907134	0.968834	0.969766	0.91893	0.975045	0.975892			
	wgEncodeAwgTfbsUtaK562CtcfUniPk	0.938346696	0.981014274	0.978712361	0.925859368	0.979261	0.980475	0.937287	0.982081	0.983783	0.92245	0.983733	0.984884	0.902774	0.965367	0.967422	0.906875	0.96748	0.96814			
	wgEncodeAwgTfbsUtaMcf7CtcfUniPk	0.905832186	0.965184947	0.961933176	0.880069293	0.94872	0.952881	0.890641	0.953348	0.958107	0.884822	0.960133	0.962654	0.873362	0.944352	0.947185	0.88571	0.950606	0.953964			
HaE2F1	wgEncodeAwgTfbsSydhHelas3Hae2f1UniPk	0.827163582	0.908472154	0.909757712	0.737618809	0.817548	0.813172	0.729115	0.810262	0.81	0.75938	0.853737	0.846418	0.758629	0.842375	0.837032	0.750125	0.836905	0.835068	0.778233	0.854034	0.853002
	wgEncodeAwgTfbsSydhMcf7Hae2f1UcdUniPk	0.799114122	0.877357215	0.880787988	0.692027097	0.782756	0.781081	0.701407	0.77711	0.774726	0.737363	0.817854	0.821963	0.717692	0.794826	0.791906	0.721209	0.804741	0.805963			
EZH2	wgEncodeAwgTfbsBroadDnd41Ezh239875UniPk	0.712369598	0.81264012	0.828814793	0.637853949	0.674638	0.687455	0.637854	0.693063	0.689426	0.52459	0.6431	0.64831	0.615499	0.682965	0.719811	0.672131	0.740009	0.740645	0.661801	0.725903	0.719539
	wgEncodeAwgTfbsBroadHelas3Ezh239875UniPk	0.712676056	0.792691363	0.814405987	0.556338028	0.595918	0.617167	0.56338	0.57907	0.577814	0.477465	0.514252	0.513424	0.567606	0.625862	0.636577	0.584507	0.626871	0.624001			
	wgEncodeAwgTfbsBroadHepg2Ezh239875UniPk	0.755538579	0.827471578	0.820837925	0.626432391	0.678059	0.66155	0.614209	0.664703	0.637044	0.658518	0.713547	0.705417	0.619557	0.667589	0.662308	0.666157	0.723844	0.71618			
	wgEncodeAwgTfbsBroadHsmmEzh239875UniPk	0.720779221	0.778484417	0.755868322	0.566558442	0.601382	0.586014	0.594156	0.620661	0.595711	0.49513	0.548051	0.520489	0.61526	0.637789	0.614313	0.63474	0.684058	0.678906			
	wgEncodeAwgTfbsBroadHuvecEzh239875UniPk	0.762073027	0.841551377	0.848284439	0.641931684	0.693806	0.679633	0.660188	0.725464	0.717597	0.68669	0.764945	0.758502	0.662544	0.72119	0.714585	0.69258	0.769566	0.779216			
	wgEncodeAwgTfbsBroadK562Ezh239875UniPk	0.688988095	0.771990741	0.753017133	0.538690476	0.55252	0.515467	0.5625	0.622926	0.574508	0.46131	0.546181	0.513367	0.561012	0.580796	0.531921	0.602679	0.635826	0.591978			
FOS	wgEncodeAwgTfbsHaibA549Fosl2V0422111Etoh02UniPk	0.903646764	0.959742396	0.962276319	0.855559528	0.923677	0.93533	0.855828	0.924437	0.936681	0.872721	0.941487	0.949858	0.861548	0.928971	0.939366	0.85547	0.936597	0.945781	0.880434	0.941159	0.947494
	wgEncodeAwgTfbsHaibH1hescFosl1sc183V0416102UniPk	0.784090909	0.853050595	0.874542341	0.613636364	0.661748	0.648445	0.611364	0.666832	0.66811	0.513636	0.528119	0.542964	0.629545	0.682044	0.682033	0.613636	0.742642	0.765794			
	wgEncodeAwgTfbsHaibHepg2Fosl2V0416101UniPk	0.887096774	0.949181051	0.953942221	0.826008065	0.902121	0.913055	0.8125	0.893403	0.905856	0.825302	0.917704	0.927207	0.826915	0.905101	0.916845	0.836391	0.911289	0.920016			
	wgEncodeAwgTfbsHaibK562Fosl1sc183V0416101UniPk	0.921465969	0.972276389	0.975239697	0.894605053	0.948033	0.959381	0.892556	0.945278	0.955986	0.904849	0.960718	0.967443	0.895516	0.951631	0.960168	0.895288	0.951691	0.962062			
	wgEncodeAwgTfbsSydhGm12878CfosUniPk	0.941513761	0.966907103	0.974224541	0.856651376	0.923579	0.928521	0.84289	0.925039	0.938058	0.862385	0.931578	0.942952	0.857798	0.931447	0.935509	0.872706	0.937213	0.947909			
	wgEncodeAwgTfbsSydhHelas3CfosUniPk	0.913907285	0.963000955	0.965342228	0.880518764	0.93083	0.939958	0.874724	0.926532	0.940351	0.892108	0.943459	0.950897	0.889073	0.942785	0.948746	0.878587	0.935084	0.946155			
	wgEncodeAwgTfbsSydhHuvecCfosUcdUniPk	0.913217477	0.963660689	0.96378898	0.88229812	0.944193	0.950654	0.87095	0.939065	0.946552	0.89403	0.954999	0.9598	0.883669	0.945963	0.952273	0.879173	0.946832	0.952044			
	wgEncodeAwgTfbsSydhK562CfosUniPk	0.948195187	0.975761333	0.977323804	0.884692513	0.945102	0.950765	0.88369	0.953201	0.957874	0.908757	0.965479	0.970863	0.89873	0.955809	0.957518	0.891377	0.953176	0.957874			
	wgEncodeAwgTfbsSydhMcf10aesCfosTam112hHvdUniPk	0.929302507	0.976779917	0.974711936	0.896477675	0.958499	0.959206	0.893549	0.959697	0.961939	0.899099	0.967007	0.967192	0.900354	0.961083	0.960411	0.903478	0.963612	0.963799			
	wgEncodeAwgTfbsSydhMcf10aesCfosTam14hHvdUniPk	0.919331742	0.971512308	0.967122813	0.890483294	0.955369	0.955403	0.892243	0.955768	0.958105	0.899582	0.962183	0.96269	0.88929	0.954429	0.95374	0.894779	0.958498	0.959532			
	wgEncodeAwgTfbsSydhMcf10aesCfosTamHvdUniPk	0.931771671	0.978136356	0.975526009	0.907104225	0.964703	0.964652	0.909516	0.965428	0.967114	0.9154	0.972151	0.972152	0.90736	0.965224	0.965379	0.911745	0.967398	0.967895			
	wgEncodeAwgTfbsUchicagoK562EfosUniPk	0.932506203	0.976051972	0.978726502	0.909925558	0.958111	0.966295	0.908933	0.954001	0.962257	0.921588	0.966858	0.972579	0.920844	0.961538	0.969313	0.912159	0.963399	0.969833			
FOXA1	wgEncodeAwgTfbsHaibA549Foxa1V0416102Dex100nmUniPk	0.830457863	0.913467945	0.917163328	0.821831453	0.896814	0.90076	0.771068	0.854724	0.869939	0.831121	0.914501	0.923576	0.811878	0.895838	0.903613	0.798607	0.888423	0.896909	0.854761	0.931408	0.935485
	wgEncodeAwgTfbsHaibEcc1Foxa1sc6553V0416102Dm002p1hUniPk	0.855234813	0.919756815	0.922099384	0.837570013	0.906332	0.915334	0.803533	0.880741	0.891022	0.834985	0.908076	0.916413	0.798363	0.871695	0.878247	0.807841	0.899206	0.905696			
	wgEncodeAwgTfbsHaibHepg2Foxa1sc101058V0416101UniPk	0.903481762	0.961758703	0.958508041	0.864519185	0.93589	0.937871	0.856052	0.930757	0.936128	0.859308	0.946907	0.95043	0.870618	0.939688	0.942943	0.870204	0.93952	0.94228			
	wgEncodeAwgTfbsHaibHepg2Foxa1sc6553V0416101UniPk	0.912096131	0.96634638	0.96238206	0.876146789	0.941413	0.943778	0.870562	0.940064	0.945102	0.880435	0.947757	0.950907	0.871609	0.939821	0.943053	0.876296	0.948431	0.950084			
	wgEncodeAwgTfbsHaibT47dFoxa1sc6553V0416102Dm002p1hUniPk	0.897395035	0.958410107	0.958164274	0.844376341	0.92301	0.926466	0.846215	0.922511	0.927746	0.853448	0.928009	0.932662	0.852835	0.926604	0.930029	0.854428	0.927879	0.932529			
GABP	wgEncodeAwgTfbsHaibA549GabpV0422111Etoh02UniPk	0.845162699	0.914740426	0.920244368	0.768945185	0.836703	0.843331	0.763267	0.832664	0.85266	0.787818	0.876602	0.892183	0.779646	0.847983	0.863707	0.778336	0.850519	0.866844	0.811858	0.891158	0.899919
	wgEncodeAwgTfbsHaibGm12878GabpPcr2xUniPk	0.860164512	0.919212717	0.926603683	0.804151978	0.88183	0.898533	0.794751	0.868067	0.887479	0.830787	0.901089	0.918433	0.817078	0.890317	0.903678	0.798668	0.879238	0.896092			
	wgEncodeAwgTfbsHaibH1hescGabpPcr1xUniPk	0.849241748	0.925469817	0.928350105	0.753345227	0.832197	0.848793	0.73372	0.809285	0.815002	0.772525	0.867321	0.879492	0.753791	0.836525	0.84919	0.755575	0.832667	0.843427			
	wgEncodeAwgTfbsHaibHelas3GabpPcr1xUniPk	0.872713415	0.940964669	0.945844793	0.832317073	0.905937	0.91762	0.814405	0.893597	0.909011	0.838415	0.920224	0.933982	0.839177	0.910817	0.922076	0.830412	0.903335	0.916411			
	wgEncodeAwgTfbsHaibHepg2GabpPcr2xUniPk	0.864816701	0.922174647	0.925717169	0.793533605	0.864568	0.873421	0.775204	0.845968	0.864443	0.803462	0.884169	0.891372	0.803462	0.885688	0.891372	0.797098	0.873565	0.88273			
	wgEncodeAwgTfbsHaibK562GabpV0416101UniPk	0.863140968	0.932177873	0.929401827	0.802215473	0.878163	0.88774	0.772378	0.85409	0.869164	0.805967	0.901147	0.908872	0.810255	0.887686	0.897427	0.775951	0.867904	0.876284			
GATA2	wgEncodeAwgTfbsHaibK562Gata2sc267Pcr1xUniPk	0.891769711	0.956611232	0.956443933	0.8494881	0.925525	0.925511	0.844834	0.921048	0.923269	0.867039	0.94134	0.941577	0.848291	0.922493	0.921385	0.851615	0.927317	0.926803	0.815832	0.893952	0.899001
	wgEncodeAwgTfbsSydhHuvecGata2UcdUniPk	0.845187892	0.918564511	0.919897488	0.799991566	0.874306	0.883368	0.775373	0.859203	0.86906	0.783994	0.889912	0.898606	0.801799	0.885903	0.892774	0.777809	0.865595	0.87168			
	wgEncodeAwgTfbsSydhK562Gata2UcdUniPk	0.860196973	0.941317188	0.938788705	0.834013932	0.906769	0.907356	0.837617	0.910006	0.912527	0.846505	0.928707	0.930601	0.841701	0.908882	0.912245	0.836656	0.909439	0.914959			
	wgEncodeAwgTfbsSydhShsy5yGata2UcdUniPk	0.844751144	0.92380541	0.92089794	0.792763158	0.872118	0.877681	0.793764	0.877667	0.88335	0.834025	0.913023	0.918605	0.802489	0.881694	0.884826	0.80349	0.88756	0.890581			
	wgEncodeAwgTfbsUchicagoK562Egata2UniPk	0.803864949	0.880354512	0.88289706	0.741892492	0.808609	0.823429	0.720791	0.790833	0.810515	0.753443	0.83397	0.843937	0.755664	0.827259	0.838874	0.743892	0.82245	0.83175			
GATA3	wgEncodeAwgTfbsHaibT47dGata3sc268V0416102Dm002p1hUniPk	0.834362563	0.919172485	0.920463917	0.759640456	0.841667	0.846438	0.771099	0.851559	0.858392	0.797242	0.883689	0.889071	0.759572	0.839932	0.843569	0.765473	0.846988	0.850925	0.772668	0.850907	0.853293
	wgEncodeAwgTfbsSydhMcf7Gata3UcdUniPk	0.776804339	0.864634691	0.872642135	0.665415102	0.739815	0.734453	0.648728	0.700496	0.692934	0.697539	0.782048	0.779063	0.700042	0.767829	0.764643	0.696704	0.776349	0.773029			
	wgEncodeAwgTfbsSydhShsy5yGata3sc269sc269UcdUniPk	0.821577429	0.903230285	0.907884772	0.75184354	0.831737	0.839662	0.742065	0.840345	0.848815	0.761622	0.880113	0.88959	0.77605	0.857207	0.858211	0.757454	0.850196	0.853861			
JUN	wgEncodeAwgTfbsSydhHelas3CjunIggrabUniPk	0.881844911	0.954618076	0.958243057	0.864132348	0.93187	0.937846	0.855567	0.925282	0.934376	0.875513	0.941295	0.947104	0.864484	0.931888	0.93856	0.865658	0.934075	0.940337	0.899448	0.957542	0.96076
	wgEncodeAwgTfbsSydhHepg2CjunIggrabUniPk	0.924351297	0.974108632	0.977107654	0.899600798	0.959246	0.964525	0.892814	0.952935	0.961096	0.913174	0.966546	0.971832	0.911776	0.968082	0.972517	0.899002	0.96022	0.965988			
	wgEncodeAwgTfbsSydhHuvecCjunUniPk	0.922043011	0.974036553	0.973364784	0.897502601	0.957514	0.9606	0.894988	0.954562	0.958895	0.907128	0.96949	0.971223	0.897763	0.959096	0.959869	0.896722	0.959956	0.961897			
	wgEncodeAwgTfbsSydhK562CjunUniPk	0.922141749	0.971903432	0.972090938	0.88696327	0.946758	0.949207	0.888515	0.946559	0.9509	0.907656	0.959793	0.962959	0.899638	0.954216	0.95518	0.890067	0.94849	0.953198			
JUND	wgEncodeAwgTfbsHaibHepg2JundPcr1xUniPk	0.863284911	0.938747224	0.943000674	0.793814433	0.876828	0.892538	0.781865	0.879608	0.891917	0.805881	0.892226	0.905133	0.815136	0.898121	0.908402	0.799203	0.889531	0.901589	0.861518	0.930132	0.934126
	wgEncodeAwgTfbsSydhGm12878JundUniPk	0.853182752	0.93088817	0.927301001	0.832648871	0.9123	0.913069	0.834702	0.904287	0.9063	0.471253	0.577742	0.53823	0.836756	0.912562	0.90868	0.829569	0.909715	0.909207			
	wgEncodeAwgTfbsSydhH1hescJundIggrabUniPk	0.874567244	0.939946092	0.94654058	0.803462051	0.887518	0.901487	0.805593	0.878872	0.900085	0.812783	0.892041	0.908579	0.800533	0.887266	0.901373	0.804527	0.885988	0.898538			
	wgEncodeAwgTfbsSydhHelas3JundIggrabUniPk	0.919030973	0.968686136	0.970406644	0.876107634	0.943762	0.950192	0.872124	0.941995	0.950762	0.882936	0.953015	0.958744	0.882042	0.946116	0.952257	0.874644	0.94513	0.950887			
	wgEncodeAwgTfbsSydhHepg2JundIggrabUniPk	0.945676101	0.983202445	0.984095216	0.912106918	0.964564	0.969007	0.921069	0.968808	0.973449	0.930346	0.97497	0.978088	0.912421	0.964869	0.968887	0.92555	0.972585	0.97514			
	wgEncodeAwgTfbsSydhK562JundIggrabUniPk	0.891580088	0.9548048	0.956115607	0.813436165	0.893372	0.902091	0.818726	0.902829	0.914476	0.829307	0.909409	0.919187	0.820065	0.899757	0.910064	0.831602	0.91251	0.920114			

	wgEncodeAwgTfbsUchicagoK562EjundUniPk	0.872067414	0.938961814	0.943463833	0.807526573	0.882891	0.897898	0.796897	0.878048	0.891868	0.82505	0.904876	0.916446	0.8149	0.890456	0.904304	0.80475	0.887962	0.900441			
MAX	wgEncodeAwgTfbsHaibK562MaxV0416102UniPk	0.841733584	0.920316744	0.920159352	0.762685802	0.851146	0.863445	0.755966	0.846959	0.861045	0.783815	0.870423	0.882742	0.763597	0.853247	0.867091	0.784669	0.867553	0.87732	0.797398	0.878464	0.883264
	wgEncodeAwgTfbsSydhGm12878MaxIggmusUniPk	0.802916751	0.885253853	0.887173598	0.703463642	0.775894	0.791818	0.6905	0.757456	0.767962	0.723516	0.802072	0.815984	0.726555	0.798963	0.810846	0.728783	0.800184	0.807474			
	wgEncodeAwgTfbsSydhH1hescMaxUcdUniPk	0.857142857	0.931214165	0.936513874	0.795169743	0.875626	0.888495	0.804056	0.876502	0.895101	0.820916	0.904479	0.916993	0.808157	0.888728	0.900376	0.802005	0.89031	0.902764			
	wgEncodeAwgTfbsSydhHelas3MaxIggrabUniPk	0.824634294	0.891505745	0.87776633	0.733316022	0.810543	0.817058	0.736865	0.816653	0.822043	0.758591	0.840265	0.849437	0.750108	0.835057	0.842719	0.763178	0.842856	0.848264			
	wgEncodeAwgTfbsSydhHepg2MaxIggrabUniPk	0.833617384	0.911956574	0.913162034	0.738176395	0.821677	0.837836	0.740307	0.822814	0.839489	0.768854	0.859568	0.873106	0.75735	0.843612	0.855806	0.757989	0.839139	0.853141			
	wgEncodeAwgTfbsSydhHuvecMaxUniPk	0.865389953	0.95147616	0.952211284	0.815986678	0.891534	0.899045	0.816264	0.897229	0.906224	0.835415	0.912766	0.920642	0.829586	0.911272	0.917218	0.825978	0.905298	0.911832			
	wgEncodeAwgTfbsSydhK562MaxIggrabUniPk	0.839350913	0.908171863	0.900093817	0.749371197	0.833562	0.838645	0.746856	0.827884	0.836263	0.768844	0.860071	0.867634	0.761136	0.84338	0.849487	0.75359	0.843532	0.849903			
	wgEncodeAwgTfbsSydhNb4MaxUniPk	0.844158707	0.925973416	0.920226768	0.785525349	0.865197	0.876381	0.783248	0.867133	0.876155	0.788758	0.870158	0.880478	0.782807	0.864687	0.872231	0.791109	0.877052	0.885775			
MYC	wgEncodeAwgTfbsSydhNb4CmycUniPk	0.821681373	0.90547608	0.910004457	0.742945797	0.826499	0.835669	0.731989	0.813358	0.823457	0.772714	0.854467	0.863806	0.765345	0.845514	0.856355	0.76389	0.844467	0.852797	0.725453	0.794268	0.796251
	wgEncodeAwgTfbsUtaGm12878CmycUniPk	0.753772291	0.827632735	0.832817725	0.657750343	0.716952	0.709506	0.677641	0.72675	0.713131	0.500686	0.534865	0.518031	0.663237	0.725876	0.731122	0.676269	0.750642	0.756693			
	wgEncodeAwgTfbsUtaH1hescCmycUniPk	0.696907216	0.782480784	0.800419837	0.540206186	0.560829	0.546471	0.560825	0.581372	0.589662	0.496907	0.494618	0.499146	0.57732	0.597544	0.583343	0.597938	0.635603	0.63803			
	wgEncodeAwgTfbsUtaHepg2CmycUniPk	0.807867731	0.881747322	0.881165064	0.735461802	0.809311	0.824263	0.722349	0.804417	0.815084	0.734892	0.823611	0.830265	0.741163	0.817318	0.828815	0.742873	0.821679	0.828893			
Pol2b	wgEncodeAwgTfbsBroadHelas3Pol2bUniPk	0.708786611	0.7928042	0.788981053	0.591631799	0.622795	0.617743	0.607531	0.655592	0.66305	0.564017	0.597895	0.60074	0.630962	0.67899	0.687264	0.627615	0.671578	0.652492	0.670603	0.733712	0.72934
	wgEncodeAwgTfbsBroadHuvecPol2bUniPk	0.757197697	0.835991888	0.826776729	0.651631478	0.704886	0.695512	0.635797	0.683818	0.667857	0.670345	0.739363	0.722865	0.646353	0.713505	0.696273	0.678023	0.734519	0.717991			
	wgEncodeAwgTfbsBroadNhekPol2bUniPk	0.720601238	0.805890336	0.820573833	0.610079576	0.661832	0.666408	0.608311	0.65375	0.643445	0.619805	0.664296	0.660052	0.628647	0.674195	0.662675	0.630416	0.687431	0.68301			
Sin3A	wgEncodeAwgTfbsHaibA549Sin3ak20V0422111Etoh02UniPk	0.754621849	0.83144463	0.840854594	0.622268908	0.677551	0.679151	0.631092	0.675276	0.646782	0.647899	0.707523	0.710466	0.64958	0.715048	0.715034	0.672269	0.741115	0.742325	0.730236	0.80292	0.804469
	wgEncodeAwgTfbsHaibHepg2Sin3ak20Pcr1xUniPk	0.795485951	0.87215254	0.874272325	0.701980654	0.776274	0.778769	0.689544	0.759629	0.758773	0.723937	0.799274	0.803023	0.722708	0.793744	0.788335	0.697374	0.791261	0.798099			
	wgEncodeAwgTfbsHaibK562Sin3ak20V0416101UniPk	0.798359672	0.88030434	0.881594626	0.701740348	0.779276	0.776636	0.687137	0.759806	0.758219	0.727946	0.813207	0.82178	0.719344	0.793845	0.7952	0.725945	0.796161	0.795096			
	wgEncodeAwgTfbsHaibPanc1Sin3ak20V0416101UniPk	0.760184201	0.860586697	0.857474877	0.669500531	0.727923	0.72901	0.661707	0.718618	0.699676	0.688983	0.762006	0.760494	0.668438	0.742423	0.72994	0.69288	0.763909	0.75735			
	wgEncodeAwgTfbsSydhH1hescSin3anb6001263IggrabUniPk	0.801068249	0.883438711	0.888122047	0.696735905	0.773661	0.776617	0.696617	0.765883	0.7682	0.72095	0.800778	0.801926	0.718694	0.79022	0.792427	0.712997	0.798309	0.805853			
SP1	wgEncodeAwgTfbsHaibH1hescSp1Pcr1xUniPk	0.823579161	0.9041584	0.909262703	0.701116373	0.771198	0.783144	0.683863	0.763293	0.764848	0.719553	0.835655	0.847321	0.722767	0.798142	0.810897	0.725812	0.80527	0.81576	0.774456	0.866068	0.872036
	wgEncodeAwgTfbsHaibHepg2Sp1Pcr1xUniPk	0.851421706	0.930698047	0.934869384	0.746896276	0.831832	0.843623	0.735783	0.816597	0.831507	0.796456	0.880165	0.890053	0.769824	0.852578	0.861704	0.770925	0.856212	0.867556			
SP2	wgEncodeAwgTfbsHaibH1hescSp2V0422111UniPk	0.861169102	0.935528088	0.938151514	0.735908142	0.802838	0.814567	0.732777	0.794867	0.810008	0.696242	0.779141	0.802981	0.743215	0.832345	0.848064	0.736952	0.834772	0.843473	0.829786	0.905048	0.911603
	wgEncodeAwgTfbsHaibHepg2Sp2V0422111UniPk	0.852601156	0.921895425	0.926247635	0.778420039	0.860354	0.887019	0.786127	0.857186	0.883334	0.796724	0.885974	0.909575	0.774566	0.848726	0.873104	0.770713	0.861965	0.886011			
SRF	wgEncodeAwgTfbsHaibH1hescSrfPcr1xUniPk	0.841238143	0.917006152	0.920797154	0.813779331	0.900167	0.917115	0.82027	0.906792	0.92289	0.84024	0.92439	0.936176	0.825262	0.908258	0.923554	0.827259	0.909517	0.922849	0.824385	0.898864	0.906635
	wgEncodeAwgTfbsHaibHepg2SrfV0416101UniPk	0.840019102	0.920323564	0.925232192	0.797516714	0.877596	0.89486	0.794174	0.875997	0.889333	0.815664	0.894459	0.907732	0.782713	0.865162	0.878681	0.799904	0.891988	0.900402			
	wgEncodeAwgTfbsHaibK562SrfV0416101UniPk	0.792880259	0.892925536	0.896370154	0.758360302	0.830183	0.844739	0.743797	0.823024	0.836958	0.765912	0.847115	0.859225	0.7589	0.836481	0.84776	0.74973	0.829236	0.839952			
STAT3	wgEncodeAwgTfbsSydhGm12878Stat3IggmusUniPk	0.756968983	0.841552489	0.838042882	0.651354535	0.701101	0.692172	0.650962	0.712388	0.688045	0.66274	0.756968	0.752403	0.666667	0.726165	0.727757	0.665096	0.742357	0.732483	0.837067	0.911605	0.913057
	wgEncodeAwgTfbsSydhHelas3Stat3IggrabUniPk	0.815062141	0.905879999	0.914029319	0.792246336	0.866876	0.880175	0.778149	0.853021	0.870428	0.807457	0.892169	0.904952	0.790577	0.867805	0.884171	0.771842	0.855797	0.870567			
	wgEncodeAwgTfbsSydhMcf10aesStat3Etoh01bUniPk	0.896494916	0.960031197	0.958729015	0.866386411	0.939858	0.941544	0.866614	0.939446	0.942352	0.881384	0.95199	0.953759	0.870079	0.942857	0.944356	0.867807	0.945154	0.947301			
	wgEncodeAwgTfbsSydhMcf10aesStat3Etoh01cUniPk	0.898618939	0.960806704	0.960284082	0.867901158	0.94011	0.94212	0.871184	0.943988	0.947233	0.878739	0.953657	0.955939	0.873908	0.944331	0.945369	0.87428	0.944673	0.947212			
	wgEncodeAwgTfbsSydhMcf10aesStat3Etoh01UniPk	0.854282766	0.933254469	0.926565206	0.84127967	0.918221	0.923426	0.842312	0.920963	0.928483	0.862745	0.938936	0.942255	0.849329	0.922805	0.923965	0.847059	0.922259	0.925226			
	wgEncodeAwgTfbsSydhMcf10aesStat3Tam112hHvdUniPk	0.895484037	0.95842514	0.956783108	0.868953556	0.941864	0.943734	0.869045	0.942582	0.945551	0.873579	0.954824	0.956746	0.877626	0.948491	0.94933	0.869917	0.941671	0.942969			
	wgEncodeAwgTfbsSydhMcf10aesStat3TamUniPk	0.903419593	0.963494498	0.96114386	0.868472736	0.941686	0.941549	0.86853	0.947623	0.950458	0.882914	0.960104	0.961422	0.877484	0.949068	0.948673	0.878061	0.949301	0.949597			
TAF1	wgEncodeAwgTfbsHaibA549Taf1V0422111Etoh02UniPk	0.745093041	0.841106784	0.850121898	0.65128728	0.711129	0.717709	0.647464	0.708996	0.708191	0.674484	0.749071	0.755476	0.673209	0.734771	0.743461	0.694112	0.761384	0.766431	0.742684	0.820804	0.825755
	wgEncodeAwgTfbsHaibGm12878Taf1Pcr1xUniPk	0.80204411	0.875397165	0.870511272	0.693024924	0.766744	0.766727	0.682446	0.759539	0.762808	0.7253	0.798823	0.803744	0.716335	0.789136	0.792537	0.716694	0.792697	0.797756			
	wgEncodeAwgTfbsHaibGm12892Taf1V0416102UniPk	0.789929616	0.87194506	0.874153468	0.690850027	0.760556	0.767182	0.663779	0.738657	0.752091	0.719004	0.79825	0.808036	0.720628	0.792505	0.802187	0.711424	0.793871	0.799836			
	wgEncodeAwgTfbsHaibHepg2Taf1Pcr2xUniPk	0.798518051	0.880407802	0.873768443	0.70439855	0.77682	0.777025	0.688475	0.761831	0.767079	0.721268	0.801819	0.807245	0.714173	0.797118	0.798271	0.710074	0.789203	0.793975			
	wgEncodeAwgTfbsHaibK562Taf1V0416101UniPk	0.80863776	0.888035024	0.891367806	0.714066234	0.789785	0.796569	0.701605	0.774341	0.7803	0.728064	0.81616	0.823473	0.730966	0.807314	0.804422	0.721577	0.799086	0.804226			
	wgEncodeAwgTfbsHaibSknshTaf1V0416101UniPk	0.80449069	0.890509728	0.894010857	0.712851406	0.782143	0.783663	0.706645	0.788264	0.783948	0.748813	0.831823	0.833498	0.731289	0.810939	0.805182	0.730376	0.812964	0.809972			
TBPI	wgEncodeAwgTfbsSydhGm12878TbpIggmusUniPk	0.794329897	0.868192817	0.87357172	0.679037801	0.745395	0.744095	0.680412	0.739062	0.727694	0.700344	0.778895	0.77675	0.697938	0.767364	0.770529	0.705326	0.772821	0.7705	0.726919	0.801791	0.803419
	wgEncodeAwgTfbsSydhHepg2TbpIggrabUniPk	0.800147221	0.877763814	0.883524751	0.663231505	0.727534	0.721922	0.668384	0.72828	0.731542	0.702245	0.782828	0.786611	0.689547	0.761253	0.762481	0.698013	0.767655	0.767426			
	wgEncodeAwgTfbsSydhK562TbpIggmusUniPk	0.810443177	0.891694535	0.901176922	0.68597338	0.753417	0.759894	0.669299	0.74402	0.74848	0.710692	0.788851	0.794748	0.711277	0.783585	0.793344	0.68729	0.775586	0.778981			
	wgEncodeAwgTfbsSydhH1hescCtbp2UcdUniPk	0.757478632	0.84315723	0.846364178	0.651353276	0.71432	0.709954	0.66453	0.719404	0.706803	0.686966	0.753455	0.749609	0.682692	0.747613	0.745256	0.690171	0.768799	0.7603			
TEAD4	wgEncodeAwgTfbsHaibH1hescTead4sc101184V0422111UniPk	0.911768472	0.965914819	0.964972462	0.87437572	0.936936	0.941728	0.871558	0.93682	0.942751	0.879242	0.948676	0.952622	0.870918	0.936105	0.939465	0.872839	0.93978	0.944735	0.833114	0.909943	0.913171
	wgEncodeAwgTfbsHaibHepg2Tead4sc101184V0422111UniPk	0.83065057	0.913605742	0.920997894	0.761904762	0.840231	0.849417	0.764085	0.841215	0.850978	0.780181	0.8673	0.878142	0.785379	0.864712	0.872733	0.780349	0.860337	0.864681			
	wgEncodeAwgTfbsHaibK562Tead4sc101184V0422111UniPk	0.859517116	0.930534876	0.927033491	0.774185547	0.854578	0.867858	0.778899	0.857542	0.869237	0.797089	0.8856	0.892571	0.779074	0.875998	0.879475	0.791963	0.874403	0.881321			
YY1	wgEncodeAwgTfbsHaibGm12878Yy1sc281Pcr1xUniPk	0.866982386	0.933970184	0.93648354	0.795613161	0.877764	0.895031	0.801346	0.878961	0.898192	0.803672	0.89294	0.90747	0.812978	0.894954	0.908284	0.807328	0.885497	0.900761	0.82822	0.904634	0.912525
	wgEncodeAwgTfbsHaibGm12892Yy1V0416101UniPk	0.860442578	0.932003765	0.937479406	0.808916169	0.884328	0.896415	0.802294	0.882408	0.898138	0.820061	0.898046	0.912193	0.804393	0.886934	0.898102	0.805201	0.885235	0.898612			
	wgEncodeAwgTfbsHaibH1hescYy1sc281V0416102UniPk	0.868024554	0.942081526	0.946959902	0.812779018	0.885678	0.897603	0.809849	0.884021	0.898686	0.811523	0.898162	0.90677	0.813477	0.889203	0.90042	0.816267	0.895627	0.905194			
	wgEncodeAwgTfbsHaibHct116Yy1sc281V0416101UniPk	0.850927958	0.928165114	0.934133584	0.736379964	0.819441	0.831093	0.739373	0.823927	0.840733	0.777689	0.863144	0.876578	0.756935	0.841168	0.848923	0.761126	0.84317	0.856698			
	wgEncodeAwgTfbsHaibHepg2Yy1sc281V0416101UniPk	0.831090753	0.910403366	0.910040831	0.752361867	0.831728	0.83603	0.74592	0.821681	0.830171	0.76138	0.842161	0.849113	0.755511	0.8411	0.847531	0.762811	0.846778	0.851477			
	wgEncodeAwgTfbsHaibK562Yy1V0416101UniPk	0.852092645	0.92837999	0.925621111	0.784030882	0.86698	0.87249	0.736692	0.842786	0.855649	0.788094	0.869716	0.87748	0.78139	0.862307	0.871426	0.747664	0.850839	0.857348			
	wgEncodeAwgTfbsHaibK562Yy1V0416102UniPk	0.864972115	0.938105382	0.939850856	0.785821536	0.870504	0.882377	0.781424	0.873961	0.8876	0.80266	0.891702	0.902083	0.806521	0.887168	0.898987	0.797083	0.880669	0.89188			
	wgEncodeAwgTfbsHaibSknshraYy1sc281V0416102UniPk	0.842122502	0.917851631	0.922469428	0.764821487	0.852766	0.866497	0.766132	0.851548	0.865386	0.779234	0.868258	0.881003	0.772191	0.852695	0.863933	0.760236	0.848466	0.858404			
	wgEncodeAwgTfbsSydhK562Yy1UcdUniPk	0.902617801	0.967550133	0.968769216	0.894240838	0.949033	0.95628	0.880628	0.932675	0.942838	0.892147	0.950413	0.959515	0.884293	0.941623	0.950571	0.849738	0.930427	0.94291			
	wgEncodeAwgTfbsSydhNt2d1Yy1UcdUniPk	0.891805035	0.950318876	0.95831724	0.853240493	0.926834	0.941872	0.843064	0.913706	0.934675	0.858061	0.934293	0.948618	0.837708	0.915775	0.932512	0.850562	0.921172	0.936723			
NFKB	wgEncodeAwgTfbsSydhGm10847NfkbTnfaIggrabUniPk	0.819081661	0.896726326	0.90191236	0.741820181	0.819613	0.841208	0.734396	0.809472	0.830079	0.765741	0.850481	0.869241	0.750894	0.841189	0.861752	0.748419	0.839084	0.855476	0.791224	0.87257	0.879786
	wgEncodeAwgTfbsSydhGm12878NfkbTnfaIggrabUniPk	0.834331935	0.911820213	0.912305492	0.765877771	0.84888	0.861114	0.744458	0.833226	0.848141	0.790144	0.877662	0.886167	0.768125	0.848608	0.859725	0.761983	0.854309	0.864717			
	wgEncodeAwgTfbsSydhGm12891NfkbTnfaIggrabUniPk	0.843044946	0.920012718	0.920080387	0.765766566	0.848441	0.859578	0.75564	0.841766	0.854748	0.789217	0.880466	0.891855	0.777136	0.858123	0.87026	0.782377	0.866224	0.875978			
	wgEncodeAwgTfbsSydhGm12892NfkbTnfaIggrabUniPk	0.829344109	0.908848314	0.917131766	0.742617345	0.822032	0.837751	0.725521	0.820321	0.839555	0.76562	0.866651	0.878127	0.766553	0.85573	0.868693	0.759714	0.840907	0.847912			
	wgEncodeAwgTfbsSydhGm18526NfkbTnfaIggrabUniPk	0.817578773	0.888158257	0.898821244	0.745439469	0.828479	0.856558	0.716418	0.794501	0.820673	0.750415	0.83862	0.858095	0.735489	0.809557	0.835103	0.778607	0.851584	0.871357			
	wgEncodeAwgTfbsSydhGm19099NfkbTnfaIggrabUniPk	0.845472441	0.918751712	0.921293047	0.774934383	0.858914	0.874258	0.770341	0.852892	0.872623	0.810696	0.89049	0.901144	0.785433	0.874039	0.88703	0.781496	0.872471	0.882593			
Pax5	wgEncodeAwgTfbsHaibGm12878Pax5c20Pcr1xUniPk	0.825820701	0.906510349	0.908647646	0.748418833	0.830351	0.837016	0.769501	0.851324	0.859359	0.758458	0.848171	0.853321	0.74862	0.828451	0.831224	0.755145	0.838073	0.839089	0.780744	0.867077	0.872871
	wgEncodeAwgTfbsHaibGm12878Pax5n19Pcr1xUniPk	0.822414238	0.905953009	0.903427651	0.742326541	0.819147	0.826452	0.733686	0.812376	0.816209	0.741811	0.837431	0.843619	0.742327	0.821673	0.83055	0.728785	0.819279	0.8253			
	wgEncodeAwgTfbsHaibGm12891Pax5c20V0416101UniPk	0.775100402	0.851853142	0.854834788	0.704417671	0.783466	0.783783	0.692369	0.759094	0.768796	0.696386	0.764168	0.780379	0.744578	0.815316	0.827059	0.708434	0.783417	0.787593			
	wgEncodeAwgTfbsHaibGm12892Pax5c20V0416101UniPk	0.792976424	0.877760364	0.881869456	0.666502947	0.748264	0.769797	0.701621	0.76353	0.774267	0.716356	0.786012	0.801008	0.710216	0.784791	0.795998	0.705305	0.776273	0.7889			
HDAC	wgEncodeAwgTfbsBroadK562Hdac2a300705aUniPk	0.763358779	0.848817104	0.849863813	0.638835878	0.697005	0.721901	0.62834	0.68363	0.692834	0.65792	0.743933	0.740239	0.660305	0.721264	0.721345	0.677004	0.75216	0.759356	0.698092	0.76683	0.77203
	wgEncodeAwgTfbsBroadK562Hdac6a301341aUniPk	0.680898876	0.738479263	0.740701347	0.575280899	0.592065	0.574748	0.541573	0.555865	0.572983	0.48764	0.542465	0.544736	0.577528	0.601019	0.608869	0.586517	0.607082	0.601533			
	wgEncodeAwgTfbsHaibH1hescHdac2sc6296V0416102UniPk	0.80080393	0.871959131	0.865770562	0.678874498	0.739099	0.743128	0.678874	0.746954	0.74062	0.690933	0.76486	0.759754	0.69138	0.755783	0.746509	0.700759	0.77547	0.76625			
	wgEncodeAwgTfbsHaibHepg2Hdac2sc6296V0416101UniPk	0.831659301	0.902594917	0.902115937	0.728404982	0.807514	0.816323	0.72653	0.799175	0.802773	0.73403	0.819425	0.822971	0.756127	0.83616	0.840215	0.7501	0.831904	0.836325			
	wgEncodeAwgTfbsUchicagoK562Ehdac8UniPk	0.673976608	0.746772413	0.760763158	0.548245614	0.581855	0.566845	0.554094	0.585825	0.564367	0.545322	0.542474	0.55352	0.565789	0.590539	0.583695	0.568713	0.602106	0.568964			
	wgEncodeAwgTfbsHaibK562Hdac2sc6296V0416102UniPk	0.79316888	0.8906353	0.888140248	0.709677419	0.785746	0.790595	0.691082	0.766205	0.761275	0.694877	0.780144	0.780837	0.73093	0.810208	0.811474	0.713472	0.78774	0.782113			
P300	wgEncodeAwgTfbsHaibGm12878P300Pcr1xUniPk	0.779046673	0.874111057	0.883468544	0.686196624	0.744914	0.740884	0.671797	0.736084	0.725762	0.723436	0.796017	0.801014	0.725422	0.784309	0.787318	0.713009	0.788861	0.799422	0.796587	0.881858	0.885349
	wgEncodeAwgTfbsHaibHepg2P300V0416101UniPk	0.856097338	0.928774351	0.927710263	0.774403074	0.857849	0.860636	0.768182	0.847452	0.854772	0.791785	0.890821	0.892988	0.783185	0.867215	0.871556	0.788857	0.874117	0.876907			
	wgEncodeAwgTfbsHaibSknshraP300V0416102UniPk	0.817261384	0.905250531	0.896468992	0.765926007	0.846216	0.849767	0.765324	0.845912	0.848569	0.786121	0.86664	0.8704	0.763846	0.848464	0.853329	0.783056	0.865737	0.868681			
	wgEncodeAwgTfbsHaibT47dP300V0416102Dm002p1hUniPk	0.81576552	0.894050819	0.893907381	0.740943018	0.817569	0.82674	0.705807	0.788734	0.796874	0.759148	0.835381	0.83393	0.74504	0.826061	0.826972	0.723466	0.822583	0.827244			
	wgEncodeAwgTfbsSydhGm12878P300bUniPk	0.84371134	0.919253399	0.921913795	0.774020619	0.853616	0.86182	0.739794	0.819941	0.81805	0.795876	0.879413	0.878567	0.775258	0.863919	0.869601	0.778969	0.857759	0.856734			
	wgEncodeAwgTfbsSydhHelas3P300sc584sc584IggrabUniPk	0.856646136	0.936602977	0.938036424	0.8117425	0.888425	0.893967	0.80151	0.882887	0.891246	0.837671	0.912587	0.917579	0.821876	0.900176	0.90399	0.820385	0.897637	0.90261			
	wgEncodeAwgTfbsSydhK562P300IggrabUniPk	0.855941427	0.926000761	0.9203229	0.781438607	0.858402	0.867197	0.774612	0.85589	0.864123	0.792718	0.883984	0.891327	0.77847	0.860573	0.865198	0.778371	0.866806	0.874097			
ELF1	wgEncodeAwgTfbsHaibA549Elf1V0422111Etoh02UniPk	0.842933492	0.916546617	0.914424558	0.779691211	0.853857	0.868329	0.763658	0.841025	0.858287	0.809679	0.87949	0.894214	0.791271	0.864078	0.87601	0.762767	0.851149	0.85834	0.828586	0.902614	0.907778
	wgEncodeAwgTfbsHaibGm12878Elf1sc631V0416101UniPk	0.8399462	0.916929125	0.91496736	0.779533737	0.856984	0.864714	0.76048	0.841566	0.852014	0.78581	0.866866	0.873539	0.778861	0.860651	0.867657	0.767093	0.859072	0.86993			

ELF1	wgEncodeAwgTfbsHaibHepg2Elf1sc631V0416101UniPk	0.887130951	0.956350404	0.956488411	0.837971465	0.909427	0.915837	0.832603	0.902054	0.91142	0.858313	0.929141	0.933358	0.847577	0.920107	0.922932	0.840514	0.912319	0.919378	[illegible]	[illegible]	[illegible]
	wgEncodeAwgTfbsHaibK562Elf1sc631V0416102UniPk	0.902313151	0.960058348	0.958854196	0.857893282	0.928098	0.929903	0.853193	0.929752	0.932967	0.869782	0.939825	0.942064	0.867754	0.934271	0.935019	0.841489	0.931805	0.932494			
	Average	0.851012688	0.919288503	0.920608586	0.785676121	0.853263	0.858752	0.782005	0.849828	0.855549	0.793211	0.865876	0.871024	0.793244	0.862673	0.867625	0.793808	0.867094	0.871677	0.797413	0.871149	0.874717
